# Early community reactions and acceptance of the Sliding-Scale Community Based Health Insurance Scheme in Ethiopia: Qualitative Findings from the treatment arm

**DOI:** 10.1371/journal.pone.0353876

**Published:** 2026-07-22

**Authors:** Zewdie Birhanu, Beshea Gelana, Nimona Berhanu, Shabu Abdulbari, Bikiltu Abdissa, Jemal Abafita, Badassa Wolteji Chala, Morankar Sudhakar, Gudeta Abebe, Yamirot Andualem, Haimanot Hailu, Dinka Irena, Hasen Haji, Tigist Teferi, Natalia Cantet, Maria Laura Alzua

**Affiliations:** 1 Department of Health, Behavior and Society, Faculty of Public Health, Jimma University, Jimma, Ethiopia; 2 Department of Health Policy and Management, Faculty of Public health, Jimma University, Jimma, Ethiopia; 3 Faculty of Health Sciences, School of Pharmacy, Jimma University, Jimma, Ethiopia; 4 Department of Economics, College of Business and Economics, Jimma University, Jimma, Ethiopia; 5 Department of Economics, Ghent University, Ghent, Belgium; 6 Department of Statistics, College of Natural Science, Jimma University, Jimma, Ethiopia; 7 Department of Economics, College of Business and Economics, Ambo University, Ambo, Ethiopia; 8 Ethiopian Health Insurance Service, Addis Ababa, Ethiopia; 9 Oromia Regional Health Bureau, Finfine, Ethiopia; 10 Center for Disability Health and Wellness, University of Michigan, Ann Arbor, United States of America; 11 CEDLAS-FCE- Universidad Nacional de La Plata, La Plata, Argentina and Partnership for Economic Policy (PEP), Nairobi, Kenya; Aix-Marseille Universite, FRANCE

## Abstract

**Background:**

Ethiopia’s Community-Based Health Insurance (CBHI) launched in 2011, has significantly improved access to healthcare. However, the program has faced several challenges, including concerns about equity, financial sustainability, and limited resource mobilization stemming from its flat-rate premium contribution system. To address these issues, a new sliding-scale premium scheme—based on households’ socio-economic status—has been piloted in 13 districts across Oromia and Sidama regions in Ethiopia. However, empirical evidence on community responses to sliding-scale CBHI models remains unexplored.

**Objective:**

To generate early empirical evidence on community responses to Ethiopia’s new sliding-scale CBHI policy (socio-economic-based premium differentiation), focusing on acceptability, perceived concerns, and community perceptions of relevance, appropriateness, affordability, equity and fairness.

**Methods:**

The study was conducted between March and May 2024 in 13 pilot districts across Oromia and Sidama regions where the sliding-scale CBHI pilot was implemented. Using a qualitative design, data were collected through 10 focus group discussions and 16 key informant interviews with community members, local leaders, and health officials, who were purposely selected to capture diverse perspectives. Discussions explored community acceptance, affordability, and experiences with the new scheme. Transcripts were translated and analyzed thematically using ATLAS.ti 7.5, following open coding and triangulation to identify key themes on barriers, facilitators, and overall community response.

**Results:**

The study found generally positive community reception of the sliding-scale CBHI scheme, which was widely perceived as acceptable, more equitable, inclusive, and better aligned with households’ economic capacity. However, this acceptance was conditional, as it depended on sustained improvements in service quality, consistent availability of medicines, and transparent management of scheme funds. Moreover, operational challenges were also reported, including household misclassification, perceived favoritism, inconsistent application of eligibility criteria, and inadequate communication of the scheme’s procedures and decisions. While stakeholders viewed the reform as a promising step toward a more equitable and inclusive system, sustaining support will require strengthened trust and consistent service delivery.

**Conclusion:**

The early implementation of Ethiopia’s Sliding-scale CBHI premium system was generally well received by community members and implementers, who viewed it as fair, contextually appropriate, and aligned with households’ ability to pay. The participatory, asset-based classification process enhanced legitimacy, ownership, and trust. However, operational and ethical challenges such as misclassification, favoritism, inconsistent criteria, and limited communication were reported. Participants highlighted the need for improved transparency, grievance handling mechanisms, and consideration of broader livelihood factors to sustain trust and ensure long-term acceptance and equity in the scheme.

## 1. Background

Since the enactment of its national health policy in 1993, the Ethiopian government has emphasized the importance of health financing reforms as a central strategy to improve population health outcomes, reduce financial hardship, and enhance equitable access to healthcare services. Among the key milestones in this trajectory is the establishment of the Community-Based Health Insurance (CBHI) program in 2011 [1–3]. Initially piloted in 11 districts, CBHI aimed to expand financial protection for informal rural communities facing barriers such as out-of-pocket costs, geographic isolation, and limited awareness of health insurance. It was also designed to improve access to essential healthcare, mobilize domestic health resources, promote equity in financing, and strengthen community ownership and participation in health system management. By 2022, the CBHI had been scaled up to cover 921 of 993 targeted districts, reaching approximately 8.9 million households, or around 40 million individuals, corresponding to 56% of the eligible population [4]. This rapid expansion reflects both the government’s commitment to universal health coverage (UHC) and the perceived utility of CBHI in reducing financial barriers to care.

Despite these achievements, the program has faced significant challenges that limit its effectiveness and equitable impact. Enrollment in CBHI remains highly uneven across different regions, ranging from 76% in Addis Ababa to only 27% in Sidama Regional State, reflecting persistent disparities in awareness, willingness to pay, trust in health institutions, and socio-economic vulnerabilities among communities [4]. One of the key structural limitations of the program is its reliance on a uniform flat-rate premium, which requires all households to contribute the same amount regardless of their income level. While administratively simple, this flat-rate approach raises concerns regarding affordability for poorer households, fairness in the distribution of financial burdens, and the overall sustainability of the scheme [5]. Empirical evidence from Ethiopia indicates that CBHI membership positively influences health service utilization and reduces catastrophic health expenditures, demonstrating the program’s potential to enhance financial protection and access to care [6–8]. However, the flat-rate premium may inadvertently exclude the most vulnerable populations, erode community perceptions of fairness, and limit resource mobilization, thus constraining the program’s ability to achieve its equity and sustainability objectives [5,9].

To address these limitations, Ethiopia’s Health Insurance Services (EHIS) proposed a shift toward a sliding-scale premium based on household socio-economic status to improve affordability, equity, and resource mobilization [5,9]. A pilot scheme has since been implemented in two regions of Ethiopia—Oromia and Sidama—to assess its feasibility and acceptability [10]. Limited evidence from countries such as Rwanda offers some lessons for implementing socio-economic–based premium systems. Rwanda’s experience, where CBHI coverage exceeds 80%, suggests that differentiated premium models can help reduce out-of-pocket expenditures, improve service utilization, and enhance equity in access to healthcare. The evidence also indicates that premium reform alone is insufficient. Successful implementation requires strong state coordination, effective governance, and sustained political commitment, as voluntary and purely community-managed CBHI models often face structural challenges in achieving universal health coverage without robust institutional support [11–15]. These findings underscore the potential of sliding-scale CBHI to enhance both financial protection and equity in low-resource settings.

Nonetheless, differentiated premiums represent a new and unfamiliar policy for many contexts including Ethiopian communities, raising potential challenges related to acceptance, trust, and willingness to participate. Households accustomed to flat-rate contributions may perceive the approach as complex, unfair, or ambiguous if its rationale, eligibility, and benefits are not clearly communicated. In addition, socio-cultural factors, local economic conditions, and prior experiences with health programs may shape community responses. Without careful attention to these perceptions, the pilot risks low enrollment, resistance, and unintended inequities, potentially undermining its goals of equitable access and financial protection.

Understanding community acceptance and early reactions is essential for several reasons. First, it provides timely insights into facilitators and barriers to enrollment, enabling adjustments in design, communication, and implementation. Second, it helps identify unintended consequences, such as exclusion of poorer households, resistance from community leaders, or misperceptions about fairness, which may undermine equity and sustainability. Third, it provides complementary measures, including awareness campaigns, targeted financial support, and participatory monitoring. Finally, early engagement fosters trust, legitimacy, and community ownership, which are critical for sustained participation and successful scale-up.

To address this evidence gap, the present study employs a qualitative approach using Focus Group discussions and key informant interviews across 13 pilot districts to explore early perception of the reform. Specifically, the study examines community members’ acceptance of the program, perceived affordability of the sliding-scale premiums, and perceptions of equity and fairness in comparison with the previous flat-rate model. By capturing these early experiences and reactions, the study provides a nuanced understanding of the social, economic, and behavioral factors influencing the feasibility of this policy intervention. This understanding is critical not only for improving adoption and deep acceptance of the sliding-scale CBHI but also for enhancing implementation quality, anticipating potential challenges, and complementing quantitative measures of health service utilization, financial protection, and equity outcomes.

Ultimately, the study highlights the indispensable role of early-stage community engagement in health policy reform. Evidence from early implementation phases can guide program adaptation, foster community trust, and ensure that reform is both technically sound and socially acceptable. In the Ethiopian context, where socio-economic disparities, cultural diversity, and historical experiences with health interventions shape community behavior, systematically understanding and addressing community perspectives at the outset is a prerequisite for achieving equitable and sustainable health financing reforms. By integrating these insights into policy design and implementation, the Ethiopian CBHI program can enhance financial protection, expand access to essential healthcare, and move closer to the overarching goal of universal health coverage.

## 2. Methods

### 2.1. Study design

This study employed a qualitative design embedded within a broader cluster randomized controlled trial (RCT) evaluating the acceptance and impact of a sliding-scale payment policy for community-based health insurance (CBHI) in Ethiopia. The trial was registered in the American Economic Association’s Registry for Randomized Controlled Trials under the title Acceptance and Impact of Introducing Sliding-Scale Payment Policy into CBHI in Ethiopia (RCT ID: AEARCTR-0011191). The qualitative component was conducted during the baseline phase immediately following program enrollment and focused exclusively on intervention sites. Guided by a phenomenological approach, the study explored community perceptions, experiences, and reactions toward the newly introduced sliding-scale CBHI scheme. It also examined beneficiaries’ and healthcare providers’ views regarding the sliding-scale and flat-rate premium systems, including related issues of affordability, fairness, trust, and healthcare service delivery. While the broader RCT quantifies the intervention’s effects on outcomes such as healthcare utilization and financial risk protection, the qualitative component was designed to provide contextual understanding of how and why the intervention may influence participation and user experiences across different study settings. Specifically, the qualitative findings help interpret trial outcomes by examining how perceptions of fairness, affordability, transparency, trust, and service quality shape enrollment decisions, acceptability, and sustained participation. In this way, the qualitative study complements the RCT by providing contextual depth and identifying implementation-related factors that may influence the effectiveness and sustainability of the intervention.

### 2.2. Selection of study setting

The selection of study sites was embedded within the design of the parent cluster randomized controlled trial, rather than determined solely for the qualitative component. Districts were first assessed on key contextual characteristics and important potential confounding variables, including demographic, geographic, and health system factors, to ensure reasonable comparability across clusters. Following this balancing process, districts were randomly assigned to treatment and control arms, thereby minimizing selection bias and reducing the influence of pre-existing differences between sites. The current qualitative study was conducted in districts allocated to the treatment arm, where the sliding-scale CBHI reform was implemented. Accordingly, the study was conducted in 13 intervention clusters (rural districts and towns) where the sliding-scale CBHI initiative was implemented across the Oromia and Sidama regional states of Ethiopia. The sites included ten rural districts- seven in Oromia (Dawo, Goro Gutu, Kimbibit, Elu, Adaba, Burka Dhintu and Dadar) and three in Sidama (Aleta Wondo, Chuko and Darra Kebado) and 3 urban settings (Ambo and Asella in Oromia and Wondogenet in Sidama). Oromia is the largest and most populous region in Ethiopia, with a population of more than forty million according to the 2023 projection. The region also accounts for the largest share of healthcare expenditure in the country and has more public health facilities than any other region. Sidama is one of the newly formed regions in Ethiopia, established in 2020, and is located in the southern part of the country. The study was conducted between March and May 2024.

### 2.3. Program enrollment

Enrollment occurred between December 2023, and March, 2024. Households in the treatment clusters were enrolled under the sliding-scale CBHI policy, which introduced differentiated premium contributions based on household economic status. Using locally defined socio-economic criteria, households were categorized into three groups—poorest/indigent, middle, and upper. In rural areas, classification drew on diverse indicators such as ownership of farmland size and productivity, housing quality, livestock ownership, income from rentals or crop sharing, perennial crops and marketable garden products, and income from skilled or casual labor. In urban areas, the criteria included the type, size, and ownership of housing, business type and tax category, income from small-scale activities or employment, rental income, urban agriculture, and other local income sources. Accordingly, the low-income category comprised households considered “very poor” or unable to pay, representing 24% of rural and 16% of urban households, with proportions varying by villages depending on local livelihood and productivity and income patterns. The *middle* category included 46% of rural and 64% of urban households, with the rural proportion subject to adjustment during the classification process. The *upper* category constituted 30% of rural and 20% of urban households. These criteria provided a flexible but standardized framework for determining household premium levels.

The classification process was conducted by community-based committees established at the kebele (village) level, ensuring transparency and contextual relevance. Government subsidies were provided to fully or partially cover premiums for the poorest households. Upon enrollment, each household received a membership identification card valid for one year, granting access to health services within their respective administrative units.

### 2.4. Sampling and data collection methods

The study population comprised community members and key stakeholders from districts implementing the sliding-scale CBHI pilot in Oromia and Sidama regions, Ethiopia. A purposive sampling strategy was used to ensure inclusion of diverse perspectives immediately after enrollment in the new policy initiative.

Participants for focus group discussions (FGDs) were purposively selected from CBHI members and non-member households to ensure variation in socio-economic status (low, middle, and high categories under the sliding-scale framework), age, education, religion, and occupation. Separate FGDs were conducted for men and women to capture gender-specific perspectives. Each FGD was conducted in the community setting. The qualitative component of the study aimed to uncover diversity of perspectives across intervention settings regarding the new reform rather than to generate equal numbers of interviews per district. Key informants were purposively selected based on their involvement in the implementation of newly introduced premium system and based on their ability to provide information-rich perspectives. Sampling continued until sufficient thematic coverage was achieved across stakeholder categories and contexts. Accordingly, a total of 16 Key Informant Interviews (KIIs) (including district or town health offices (2), CBHI focal persons at district level (4), PHCU directors (3), Health Extension Workers (HEWs) (3), village (kebele) leader (2), community leader (1) and 10 FGDs (6 men and 4 women) were conducted across Oromia and Sidama regions. In Oromia, 10 KIIs and 7 FGDs were conducted—comprising four men’s FGDs and three women’s FGDs while in Sidama, 6 KIIs and 3 FGDs were carried out, including two men’s FGDs and one women’s FGD (Table 1). Fewer women only FGDs reflected contextual realities rather than lower priority of women’s perspectives. In many rural Ethiopian settings, men more often hold formal community leadership roles and participate in public discussions related to local governance including CBHI activities and enrollment decisions. Women’s participation can be constrained by domestic responsibilities, time limitations, and gender norms. To ensure meaningful inclusion, separate women-only FGDs were undergone to reduce power imbalances and create a comfortable space for open discussion. Thus, although smaller in number, the women FGDs were designed to capture rich and authentic perspectives. Data saturation was used as the guiding principle for determining the number of FGDs and KIIs.

**Table 1:** Distribution of qualitative data collection activities by region.

Region	Number of sites	KIIs	FGDs – Men	FGDs – Women	Total Data Collection Events
Oromia	9	10	4	3	17
Sidama	4	6	2	1	9
**Total**	**13**	**16**	**6**	**4**	**26**

Each FGD included 8–12 participants and lasted 60–90 minutes while KIIs ranged from 40 to 72 minutes. Among the FGD participants specifically, 16 participants were from the high socioeconomic category, 40 from the middle category, and 20 from the low category. Participants represented a wide range of ages, educational backgrounds, and religious affiliations. Occupations varied, including farmers, small business owners, daily laborers, and other community roles. Socioeconomic status, as stratified by the sliding-scale CBHI framework, included individuals from low-, middle-, and upper-income households, as well as non-members.

KIIs were conducted with individuals holding various positions in the health system and community governance. These included district and town health office heads, directors of primary health care units, CBHI focal persons, health extension workers, village leaders, EHIS branch managers, and non-CBHI members. Participants were selected purposely to ensure representation of relevant stakeholders involved in the implementation and management of CBHI, providing comprehensive insights into community perceptions and operational experiences.

All discussions and interviews were conducted in participants’ preferred languages by experienced and trained facilitators, interviewers, and note-takers, with minimum of master level educational qualifications in public health. Senior researchers (PI) made close oversight of the data collection process and ensured the quality of the data. Interviews and discussions were audio recorded using a digital voice recorder and conducted in participants’ preferred languages by experienced facilitators, interviewers, and note-takers, with oversight from qualitative researchers to ensure data quality.

Instruments and phenomena of interest: pre-tested, open-ended guides (S1 Appendix) were used to collect qualitative data from FGDs and KIIs, exploring perceptions of the sliding-scale CBHI policy initiative, including its benefits, implementation challenges, gaps, and factors influencing enrollment and participation. The instrument focused on acceptance and uptake, satisfaction with the scheme, concerns and gaps, barriers and facilitators, and recommendations for improvement. Acceptance and uptake were explored through perceptions of the program’s appeal, fairness, equity, inclusiveness, household stratification, and benefits compared with the previous flat-rate system, including its potential to enhance financial viability and service quality. Concerns and gaps focused on issues such as household stratification, policy awareness, and community engagement, while barriers and facilitators considered the stratification process, communication, community acceptance, and service-related challenges. Participants were also asked to provide suggestions for improving the policy at both local and district levels.

### 2.5. Data analysis

FGDs and KIIs were transcribed verbatim and translated into English for analysis using ATLAS.ti 7.5, following an open-coding approach. Details of the codes and quotations underlying this analysis are provided in S2 Appendix -Codes with Quotations. Two independent coders (NB and BG) iteratively reviewed and coded the transcripts, while a third coder (ZB) verified the emerging codes to ensure consistency and reliability. Through an interactive process of initial coding, discussion, and refinement, the broader research team reached consensus on a coding framework, which was then used to organize codes into sub-themes and broader themes through an inductive approach. Coding agreement was achieved through repeated consensus discussions and refinement of the codebook among the research team. Key findings were presented narratively under each theme and sub-theme, with representative participant quotes used to illustrate and support the results. Triangulation was conducted by systematically comparing findings across FGDs and KIIs, as well as across different respondent groups (community members and key stakeholders) and regions (Oromia and Sidama), to identify converging and diverging patterns. This multi-source comparison strengthened the credibility, dependability, and depth of interpretation. Findings are presented narratively under each theme and sub-theme, supported by representative participant quotations.

### 2.6. Data quality control measures

To minimize potential response and social desirability biases, several strategies were employed throughout the data collection process. FGDs were conducted separately for men and women to create more comfortable environments for open opinion sharing and to capture gender-specific dimensions or perspectives. FGDs and interviews were facilitated by trained independent data collectors who were not directly involved in local CBHI implementation and who have extensive experience in facilitating FGDs, helping to reduce courtesy bias toward program authorities. Skilled probing techniques and neutral questioning were used to encourage honest, detailed responses and to explore both positive and negative opinions. Data collection sites were selected to ensure privacy, convenience, and neutrality, allowing participants to speak freely without fear of being overheard by local officials. A detailed informed consent process emphasized voluntary participation, confidentiality, and the right to decline any question or withdraw at any time. Finally, triangulation across focus group discussions, key informant interviews, field notes, and multiple respondent categories was used to validate emerging themes and strengthen the credibility of findings. The analysis was supported by qualitative software, with multiple coders involved to ensure rigor, transparency, and consistency. Key findings were also presented to stakeholders and representative community members for validation, ensuring that the results accurately reflected the perspectives of the study population. The Consolidated Criteria for Reporting Qualitative Research (COREQ) guideline [16] was followed throughout the study design, data analysis, and reporting of the manuscript (S3 Appendix).

### 2.7. Ethical considerations

Ethical approval for the study was obtained from the Institutional Review Board (IRB) of Jimma University (Reference No. JUIH/IRB/2023). Permission letters were secured from the relevant regional, zonal, district, and community authorities prior to data collection. The purpose and objectives of the study, as well as the nature of participation, were clearly explained to all respondents. Written informed consent was obtained from each participant before enrollment. Participants were assured of the confidentiality and anonymity of the information provided, and all data were handled with strict confidentiality throughout the study. No individuals other than the participants and the researchers/facilitators were present during the discussions and interviews.

### 2.8. Research team and reflexivity

The study was conducted by a multidisciplinary research team (six females and ten males) with complementary expertise in qualitative and quantitative research, public health, health systems and policy, economics, statistics, and pharmacy. The broader team included researchers from public health and health systems backgrounds (ZB, BG, NB, MS), economics and statistics backgrounds (ShA, JAb, BW, BA, NC, and ML), as well as health system practitioners and managers (GA, YD, HHa, DI, HH, TF, and HHu). This multidisciplinary composition contributed diverse methodological and disciplinary perspectives throughout the study process.

The study was led by Prof. Zewdie Birhanu, Principal Investigator of the project and senior faculty member at Jimma University. He is an experienced qualitative researcher with extensive expertise in health policy and systems research, public health, and health behavior research, and has authored more than 160 peer-reviewed publications. The two coauthors most closely engaged in the qualitative data analysis and interpretation were Mr. Beshea Gelana and Mr. Nimona Berhanu. Mr. Beshea Gelana is a faculty member with experience in health systems and policy research and mixed-methods public health research, and is currently pursuing a PhD in Health Systems and Policy at Jimma University. Mr. Nimona Berhanu is also a faculty member at Jimma University with a pharmacy background and research experience in health systems, public health, and health policy, and is currently undertaking PhD training in Health Systems and Policy. Four members of the research team (BG, NB, BA, and ShA) directly involved in conduct of interviews and discussions. However, remained aware of their potential influence on the data collection process and continuously reflected on how their professional backgrounds, assumptions, and interactions with participants might shape the responses and interpretation of findings.

The qualitative interviews were conducted by trained researchers experienced in qualitative data collection and familiar with the local context and language. Interviews were carried out using semi-structured interview guides in private settings to facilitate open discussion, participant comfort, and confidentiality.

The research team recognized that their professional and disciplinary backgrounds could shape the interpretation of participants’ accounts. To enhance reflexivity and minimize potential bias, regular debriefing sessions were conducted throughout data collection and analysis to reflect on assumptions, discuss emerging findings, and consider alternative interpretations. The diversity of perspectives within the team helped challenge individual assumptions and strengthened the rigor, credibility, and trustworthiness of the analysis.

## 3. Results

### 3.1. Background characteristics of the FGD participants and Key informants

A total of ten FGDs were conducted across the two study regions—seven in Oromia and three in Sidama. Each group consisted of six to twelve participants, yielding a total of 100 participants. Better representation was ensured by conducting four FGDs with female groups and six with male groups. The demographic profile of the participants was diverse. The average age was 40.6 years, with most participants (64%) falling within the 26–45-year age range. In terms of educational attainment, primary education was the most common (62%), followed by those with no formal education (21%) and a small proportion with secondary or higher education.

Participants represented various religious backgrounds, with 37% identifying as Orthodox Christian, 36% as Protestant, and 27% as Muslim. The majority (70%) were farmers, followed by small business owners or merchants (17%) and daily laborers (10%). Based on the sliding-scale classification used in the study, nearly half (49%) of the participants were from middle-income households. Most participants were also active members of the Community-Based Health Insurance (CBHI) scheme, with 86% reporting renewal status and 2% as new members, while 12% were non-members. In addition to the FGDs, 16 key informant interviews (KIIs) were conducted with a range of stakeholders’ involved in or knowledgeable about CBHI implementation. These included district and town health office heads, primary health care unit directors, CBHI focal persons, health extension workers, kebele or village leaders, and non-CBHI members. The informants represented intervention sites, providing valuable perspectives from multiple levels of the health system and community structure.

### 3.2. Community’s positive reaction and perceptions towards socio-economic based premium contribution scheme

This qualitative study uncovered that the community positively reacted to the new premium contribution approach-recognized and accepted the economically-based premium contribution scheme, known as the sliding-scale scheme, as a fair and equitable policy initiative by the government. Even though there were some concerns, both communities and stakeholders positively reacted and accepted the new scheme, seeing it as a crucial step toward creating a more equitable and inclusive CBHI system. The following reasons and justifications were commonly cited for its positive acceptance and reactions of the new scheme.

#### 3.2.1. Equity and Fairness in citizen’s contributions.

Many respondents viewed the sliding-scale scheme as a major improvement over previous premium contribution practices, where all households contributed a flat rate premium regardless of their economic status and the new scheme was perceived to be fair and aimed for equity in contribution to the scheme. Participants (communities) praised the new scheme for addressing long-standing concerns about inequity in contribution. Many participants perceived that ensures that the financial burden is distributed more fairly, particularly benefiting poorer families, where it is described as “Pro-Poor Policy initiative.

The recurring views reflected that new scheme has effectively addressed community concerns about the previous policy, where everyone contributed an equal amount, regardless of their economic status. One key informant noted, “*I think people appreciate this economic-based health insurance because it considers whether a person has wealth or not. It’s very good because contributions are based on people’s economic capacity. I believe people will be more willing to pay because it reflects their economic ability”* **[KII-Health Extension Worker].** Another respondent shared, *“It was never fair for the highest-income and lowest-income people to pay the same amount. This system of collecting membership contributions according to their wealth status is also acceptable to the community”* **[KII-District level CBHI focal].**

In view of pro-poor idea, the perspectives of participants suggested that the household’s stratification into different socio-economic strata within the new scheme was particularly viewed as beneficial for poor or indigent households, who previously struggled with flat-rate contributions. The approach was appreciated for properly recognizing and supporting the poorest segment of the society, distinguishing it from the previous approach, which failed to adequately target those who were indeed, considering the new initiative as a positive step by the government. A FGD participant described the appropriateness of the initiative as: *“The government has done a great thing. We are very happy that the poor can get health services though after contributing what they can afford* **[FGD-Men].**

In view of pro-poor perspectives reflected by the participants, the new scheme also appreciated for its inclusiveness nature-as it includes and engages households of all categories. Many participants appreciated that the new scheme promotes inclusivity by allowing broad participation across the community groups, enabling everyone to contribute according to their economic capacity. A key informant stated, *“This new scheme fosters inclusiveness. It makes a balanced contribution and allows everyone to participate.”* **[KII-PHCU Head].**

#### 3.2.2. Alignment with CBHI core principles: Equity, solidarity, and mutual support.

Regarding the alignment with the core idea of CBHI and the promotion of mutual support and solidarity, there was a common consensus among the interviewed stakeholders and community members that the new scheme aligns with the fundamental principles of CBHI—commonly expressed as “contribute according to ability and receive services and care according to need.” A Key informant noted that*, “The new scheme allows the community to contribute according to their capacity and gets treatment service according to their needs. If the poorest, the middle class, and the better off class contribute equally, we can’t say that we have proven justice*.” **[KII-CBHI Branch Manager].** In the view of many participants, both community members and key informants, the new scheme was described as best fitting the concept of “community solidarity in social health insurance” or “mutual aid within a contributory health system.”

Participants’ view reflected the understanding and practice of solidarity or mutual support, where community members recognize the importance of collectively supporting those in need, with contributions tailored to each household’s capacity. The Community realized that insurance is all about helping each other, especially helping those who have some deficit to help themselves and such a helping process is appropriate when the contribution considers the households capacity to contribute. A FGD participant mentioned that *“In our community better off households are let to pay higher premium. Even though all human beings are born at 9 months, economically everyone is not in equal status in possessing wealth. So, the classification into different categories: lower, middle, and higher is based on household wealth or income. This type of classification is very important as health insurance is also based on the idea of supporting each other”* **[FGD-Men]**.

#### 3.2.3. Improved financial viability and resource generation.

Key informants across study districts consistently appreciated the relevance of the new approach for its potential to mobilize better resources from the community through member’s contributions. In their view, the sliding-scale has significantly enhanced the scheme’s ability to mobilize resources, resulting in higher revenue collection compared to the flat-rate system. The head of the district health office described the situation as, “*Last year, when the scheme was a flat scale approach, we collected 13 million Birr (240 million USD) from members. However, this year, through this new sliding-scale approach, we collected more than 31 million Birr (553million USD.”* **[KII-District CBHI focal person].** Another CBHI manager at a district level explained*,* “*Last year, we collected around 5.5 million Birr using a flat scale. But this year to up to now, we collected around 11.1 million Birr following the implementation of a sliding-scale scheme. This means that the amount of premium collected this year-following the implementation of sliding-scale scheme- is more than two times higher than the amount collected in previous year (under flat scale scheme* [KII-**Head of District Health Office].**

The positive reaction and early acceptance of this scheme by the stakeholders were not merely evaluated in terms of the quantity of money mobilized; rather they believed that the new scheme enabled them to generate more financial resources for healthcare in an equitable and fair way. A CBHI focal person stated that reflected in as, “*In previous years, it was unfair that individuals from all economic backgrounds, including the rich, middle class, and poor, were required to pay the same amount. Now, by aligning contributions with each person’s wealth status, we have successfully raised sufficient funds this year and were able to pay last year’s outstanding debts to health facilities*.” **[KII-District CBHI focal person].**

Across the scheme, CBHI managers explained that the new scheme enabled them to recover the financial deficiencies their scheme had faced during the previous years*.* A respondent explained that the sliding-scale approach has helped recover the financial system of the health facilities, allowing them to clear previous debts and avoid bankruptcy. A district level CBHI focal person said: “*The sliding-scale is an opportunity for our district; it helps us overcome the bankruptcy we experienced previous year, when our scheme was on the verge of collapsing and ceasing activities*.” S4 Appendix provides details on the substantial increase in resource-premium contributions mobilized by members immediately after the initial implementation of the new scheme.

#### 3.2.4. Perceived contribution to service improvement.

Many participants, especially KIIs participants, reflected an optimistic view that the new scheme had the potential to support reliable health service provisions to its members, as it increased financial resources which will lead to better service provision in health facilities, including improved availability of medicines and overall service quality. *“Now, our four health centers will be able to provide adequate services with sufficient medicines available for our community.”* **[KII-District CBHI focal person].** Another health manager expressed, “*The sliding-scale also helps to solve the problem of drug and laboratory accesses. This year, we collected the premium two times higher than that of the previous year. This will enable us to solve the shortage of supplies.”* **[KII-Head of District Health Office].**

Additionally, the new premium contribution was perceived as aligned to market and level of inflation Ethiopia was facing: There was broad recognition that the price of healthcare services, especially medicines, has significantly increased, making the new premium payment increment necessary and acceptable. A key informant: One participant said*: “Increasing the amount of payment is acceptable and important for our community and health facilities because we know that the price of medicine has been increasing over time.”* **[KII-Kebele Leader].** Moreover, there was growing recognition that, due to the increased cost of healthcare supplies, the previous flat rate premium contribution was insufficient to cover healthcare expenses, and the new premium was, therefore, perceived as reasonable to withstand challenges related to increased cost of delivery service. In support of this idea, one participant said; *“Compared to the current inflation and the cost of services provided, the amount of money collected from insured members was not enough to cover health services costs. However, this year we were able to collect more money due to the sliding-scale, and I hope we will withstand the impact of inflation on service availability”* **[KII-Town Health Office Head].**

#### 3.2.5. Perceived affordability of the new CBHI premium contribution.

Overall, most study participants perceived the new community-based health insurance (CBHI) premium contribution as fair, affordable, and justified, recognizing that it considered households’ economic capacity and was necessary to sustain quality healthcare. The community expressed readiness to pay the increased premium if accompanied by improved service availability. A district level CBHI focal person said*: “Our community has no complaints regarding this year’s contribution amount. All they need is adequate service; they are ready to pay even more if the services they need are always available.* Table 2 presents the key dimensions of premium affordability accompanied by relevant supporting quotations.

**Table 2:** Key positive perceptions of the new CBHI premium contribution.

Key Theme	Brief Description	Supporting Quotation(s)
1. Affordable and Reasonable Compared to Rising Healthcare Costs	Participants acknowledged the premium increase as reasonable and proportionate to the rising cost of healthcare and medicines.	*• If we compare it with the services they receive, the membership contribution is not enough and should increase… the cost of medicines is increasing.”* **[KII-PHCU Head]** *• “If we pay the same amount each year while the price of medicine increases, how can the government provide those medicines for us?”* **[FGD-Men]**
2. Justifiable Increase Compared to Previous Low Premiums	The former flat-rate premium was viewed as inadequate to cover household health needs. The new contribution was seen as necessary and fair.	• *“Do you think 410 birr can purchase medicine, laboratory services, and other inputs for an entire family for one year? The previous contribution was too low.”* **[KII-District CBHI focal Person]**
3. Affordable Compared to Private Health Costs	The CBHI payments were viewed as far cheaper than private clinic fees, reinforcing perceptions of value for money.	*• We all know how much we pay at private clinics—up to 3,000 birr for one laboratory test. The problem is when we pay for insurance but still buy medicine outside.”* **[FGD-Men]**
4. Low-Cost Relative to Benefits Received	The premium was considered small compared to the service benefits received, particularly for low-income households.	• *Insurance service benefits us, especially poor individuals… the payment for health insurance membership is very low compared to the advantages we get.”* **[FGD-Women}**

#### 3.2.6. Perceived relevance and community acceptance of socio-economic stratification.

Across all study sites, both key informants and community participants generally perceived the household stratification system—classifying households into indigent, middle, and better-off categories—as relevant, contextually appropriate, and broadly fair. The approach was seen as reflecting local socio-economic realities and aligning with community norms of reciprocity and shared understanding of wealth. In many cases, participants emphasized that the process was implemented through community-based structures that enhanced transparency and acceptance. As one key informant stated, *“We have identified households to high, medium, and indigent categories with fairness”* [KII-District CBHI focal person]. Similarly, a male FGD participant noted, *“Our community thinks that is fair and equitable… They assessed how much asset wealth each household owns. That evaluation was appealing and nice”* [**FGD-Men**].

A key reason for this acceptance was the use of locally grounded, asset-based criteria that reflected how communities themselves define wealth and economic capacity. In rural settings, this included land size and productivity, livestock ownership, crop production, and housing conditions. One key informant explained, *“In rural areas, we assess locally known products such as teff, oxen, cows, tractors, trees, and land area”* [KII-District CBHI focal person], while another added, *“We used ownership such as land and livestock, and we also counted their cattle and estimated their annual grain production”* [KII-**village leader**]. In urban areas, participants highlighted housing quality, business ownership, shops, and vehicles as appropriate indicators, with one respondent noting, *“At the urban level, we divided households into three categories according to their housing, cars, shops, and other assets”* [KII-**District CBHI focal person**].

Beyond economic assets, some participants appreciated that the system also incorporated social and health-related vulnerabilities. Households with illness, disability, or dependency were recognized as needing special consideration, which was viewed as reflecting community values of solidarity and compassion. As one key informant explained, *“We classified people who were dependent on others for various reasons, such as illness and disability, and had no income, as poor in both rural and urban areas”* [KII-**District CBHI focal person**].

Participants largely reported that the participatory and community-led nature of the stratification process further strengthened its perceived legitimacy. Because local committees were composed of individuals familiar with household circumstances, many participants felt the outcomes were credible and socially accepted. As one respondent summarized, *“The community itself knows who is better off and who is not; that is why we accepted the result”* [**FGD-Men**].

### 3.3. Concerns in household stratification and premium classification process

Although the sliding-scale CBHI premium system was generally accepted, participants raised concerns about fairness, affordability, transparency, and consistency in household classification. These issues were reported across community and key informant interviews and were linked to weak criteria, limited transparency, and implementation challenges. The following section presents key concerns supported by participant quotations.

#### 3.3.1. Concerns about the affordability of the new premium contribution.

Despite broad support, some concerns emerged regarding affordability, timing, and perceived value of the premium increases. These concerns were context-dependent rather than an outright rejection of the scheme. The main challenges to premium affordability included declining household incomes from market fluctuations, rising living costs and inflation, difficulty paying the full premium at once, sudden and large premium increases, a perceived mismatch between premium cost and service quality, and poorly timed collection periods that coincided with cash shortages (Table 3).

**Table 3:** Participants’ concerns about the affordability of new premium contribution.

Key Theme	Brief Description	Supporting Quotation(s)
1. Income Decline Due to Market Fluctuations	In cash-crop-dependent areas, declining market prices (coffee, khat) reduced households’ ability to pay.	*• Our community’s income is mainly based on khat, and now that the price has dropped, it has become challenging to pay.”* **[FGD-Women]** *• People depend on coffee… this year’s coffee market is very low, and now no one has money.”* **[FGD-Women]**
2. Rising Living Costs and Inflation	Inflation and basic living expenses limited disposable income, making premiums feel heavier.	*• This year, the high membership payments, combined with the rising cost of living, have made it difficult for many community members to afford insurance membership.”* **[FGD-Men]**
3. One-Time Lump Sum Payment Challenge	Paying the full premium at once was difficult; participants preferred installment options.	*• “Paying the premium in one lump sum is difficult for our community. We would prefer to contribute in multiple rounds.”* **[FGD-Men]**
4. Abrupt and Excessive Premium Increase	The steep, sudden rise (up to 4x the previous rate) caused financial strain and dissatisfaction.	• *Last year it was 410 ETB. This year, the medium is 1260 ETB and the maximum is 1710 ETB. Some people were not happy.”* **(FGD-Women)** *“An individual who used to pay 420 birr is now paying 1720 birr.”* **[KII-District Health office Head]**
5. Perceived Cost–Value Discrepancy	Premiums increased, but service quality (medicine availability) did not improve, eroding trust.	*• The payment increased more than double, and medicine availability decreased… members suspect the payments are not being used for insurance.”* **[FGD-Men]**
6. Poor Timing of Premium Collection	Payment deadlines often coincided with cash-poor seasons, worsening perceived unaffordability.	*• The enrollment period came when people did not have cash on hand. It would be better if they collected payments when we have money saved up.”* **[FGD-Women]**

#### 3.3.2. Perceived inequities and weaknesses in household stratification.

Although participants generally accepted the principle of linking CBHI contributions to households’ ability to pay, many community members and stakeholders expressed concerns about the fairness, accuracy, and transparency of the household stratification process. Across both rural and urban settings, participants described frequent cases of misclassification, often attributing them to weak asset assessment methods, inconsistent application of criteria, and limited community involvement in the process. A participant in men FGD explained, *“During classification, there was a mistake. Those who should be categorized as poor were classified as middle, and vice versa”* [FGD-Men]. Similarly, a woman stated, *“They categorized me to a higher level while they knew I was a poor mother raising children without a husband”* [FGD-Men].

Participants argued that the process relied heavily on superficial or inappropriate indicators such as housing type, clothing appearance, or land size, which did not adequately reflect actual economic capacity. Rural participants particularly criticized the use of landholding as a proxy for wealth, emphasizing that productivity and income varied widely despite similar land size. As one woman explained, *“Classifying households according to their resources rather than their land area is incorrect… Even a person without land is sometimes made to pay”* [FGD-Women]. Others described how committee members assessed households *“simply by observing the type of house and the clothing of its occupants”* [FGD-Men], creating a perception that categorization decisions were arbitrary and technically weak. Participants also noted that the three-level classification system (indigent, middle and upper) failed to capture the economic diversity within communities, particularly among households categorized as “middle level.” Consequently, several participants recommended increasing the number of categories to improve fairness and proportionality of contributions.

#### 3.3.3. Transparency, social bias, and institutional challenges.

Concerns about transparency and accountability emerged consistently across interviews and discussions. Community members frequently reported that they were not adequately informed about the criteria used for classification and had little opportunity to challenge decisions. One participant noted, *“The community did not understand the criteria, and no adjustments are made even if there are complaints”* [FGD-Women]. Another added, *“Once they decide on the payment amount, they cannot reconsider it for those who complain”* [FGD-Women]. In their view, these experiences contributed to feelings of exclusion and weakened trust in the intended equity of the system among many community members.

Stakeholders also acknowledged implementation challenges at the local level, including favoritism, quota-related pressures, and limited technical capacity. A district CBHI focal person explained that some committees *“were categorizing their close friends or relatives to lower-level category even though they have a high economy”* [KII-District CBHI focal person]. At the same time, better-off households were reportedly reluctant to accept higher contribution levels and sometimes concealed assets to avoid increased payments. According to a village leader, *“Those who can pay still want to be classified as poor”* [KII-village leader]. Key informants further described difficulties in applying fixed proportional quotas across economic categories, particularly in rural areas where households did not fit neatly into predefined classifications. In addition, inadequate training, limited institutional support, and insufficient community sensitization were reported to contribute to inconsistent implementation. As one CBHI branch manager reflected, *“Limited training and support during screening made it difficult to classify properly”* [KII-CBHI branch manager].

## 4. Discussion

Ethiopia introduced the Community-Based Health Insurance (CBHI) program in 2011 as a central pillar of its health care financing reform, aiming to enhance financial protection for the informal sector, expand access and utilization, promote equity in health financing, mobilize domestic resources, and foster community ownership (2). As of 2020, 862 districts (78% of all districts) in Ethiopia were enrolled in the CBHI scheme, covering approximately 8.4 million households (around 39 million individuals), which represents 56% of the eligible population. Although the scheme has achieved notable progress in expanding coverage and protecting households from catastrophic expenditure (1), a structural limitation has persisted: the use of a flat-rate premium system that requires all households to contribute equally regardless of income. This design feature has constrained enrollment among low-income households, undermined fairness, and weakened revenue generation, as wealthier groups have contributed below their capacity and willingness to pay (3,4). In response, Ethiopia initiated a socio-economically differentiated premium contribution model in 13 districts across Oromia and Sidama regions. The new approach links premium payments to households’ relative economic status, with the goal of improving equity, sustainability, and inclusiveness in CBHI financing (7). The current study explored early community and implementer responses to this policy initiative, focusing on perceptions and experience of fairness, affordability, acceptability and legitimacy during the initial implementation phase. Such early assessments are critical, as the initial reactions of communities and frontline implementers often determine the long-term adoption, institutionalization, and sustainability of new policy interventions (5,6). The key findings that emerged from the study are discussed below in a thematic manner.

### 4.1. Early acceptance, social legitimacy, and perceived equity

Overall, findings from this qualitative inquiry demonstrate a strong early endorsement of the differentiated premium model by both community members and implementers. Respondents viewed the new model as fair, contextually relevant, and socially just, as it allows households to contribute based on economic capacity rather than a uniform flat rate. The reform was widely interpreted as a corrective measure to the inequities embedded in the previous system, which was often perceived as regressive and unjust. This perception of fairness appears to have been central to the model’s early acceptance, reaffirming that equity-oriented reforms gain traction when they resonate with both community values and moral expectations of justice and reciprocity. The reform’s alignment with traditional systems of mutual assistance and solidarity further reinforced its legitimacy [10]. In many Ethiopian rural communities, collective risk-sharing and social support mechanisms are deeply embedded in cultural norms [6,17]; better-off families traditionally help poorer households during illness or hardship. By institutionalizing these social values into a formal financing mechanism, the differentiated premium model indeed bridged the gap between state policy and community practice. This normative congruence transformed the reform from a technocratic fiscal adjustment into a socially meaningful initiative, echoing evidence from similar contexts that cultural coherence enhances policy acceptability and compliance [8,9]. From the perspective of implementers, the sliding-scale CBHI reform was particularly valued for its innovative combination of equity and fiscal utility. By linking household contributions to income, the scheme represents a novel approach in which citizens’ pay according to their ability, promoting social fairness while simultaneously expanding and diversifying the resource pool. Unlike flat-rate systems, this design enhances revenue predictability, prevents financial shortfalls, and strengthens the long-term sustainability of the CBHI program. These findings align with broader evidence that income-sensitive contribution mechanisms not only improve resource mobilization but also enhance financial protection, highlighting the sliding-scale approach as a promising model for achieving both fiscal stability and equitable access to health services [13].The dual foundation of moral legitimacy (equity and justice) and instrumental legitimacy (sustainability and solvency) thus created a synergistic base for the early success of the pilot.

### 4.2. Conditional acceptance and the fragility of early enthusiasm

While the overall response was positive, the acceptance observed should be interpreted as conditional and potentially fragile. This fragility is particularly evident in the context of reported operational challenges. Concerns around misclassification of households, perceived favoritism in beneficiary identification, and inconsistent application of eligibility criteria may undermine trust in the system. Trust, once eroded, can have a disproportionate effect on participation decisions, especially in community-based schemes where social perceptions and collective experiences strongly influence enrollment and renewal. The community’s willingness to support the new scheme appears strongly contingent on the expectation that higher or differentiated contributions will lead to tangible improvements in service access and quality—particularly the consistent availability of medicines, responsive service delivery, and transparent management of funds. Similar findings have been documented elsewhere, where perceived disconnect between premium payments and service quality undermines community trust and enrollment retention [18,19]. Thus, the early enthusiasm may not be self-sustaining; it represents a window of opportunity that must be consolidated through credible performance and visible results as the initiative implementation progresses.

Another dimension of conditionality relates to the timing and process of implementation. Several participants described the reform as abrupt, with limited preparatory communication and inadequate time for households to plan financially—especially in the context of inflation and fluctuating market prices especially for rural livelihoods. This underscores a broader lesson in policy diffusion: even well-designed reforms risk resistance when communities perceive procedural unfairness or limited consultation. Transparent communication, adequate transition periods, and participatory planning can mitigate such perceptions and enhance legitimacy.

### 4.3. Stratification and the challenge of operational fairness

The asset-based household stratification process was widely accepted in principle, particularly given the difficulty of accurately estimating household income in largely informal rural economies [20,21]. However, its execution presented implementation challenges. Communities raised concerns about overreliance on land size as a proxy for wealth without sufficient consideration of productivity or household dependency ratios. Moreover, gaps in transparency in the classification process—where some households learned of their economic category only during premium collection—created perceptions of arbitrariness. Misclassification risks, whether real or perceived, can erode trust and foster grievances, particularly in settings where social hierarchies are sensitive. These findings reflect a common tension between technical rationality and social legitimacy in community-based targeting systems. While asset-based stratification offers administrative simplicity and objectivity, it may fail to capture the fluidity of rural livelihoods and non-material indicators of vulnerability. As seen in community health financing experiences in Ghana and Rwanda [11,22], the perceived fairness of targeting processes—more than their statistical precision—determines public acceptance and compliance [23–25]. However, these contexts also differ from Ethiopia in important ways that affect transferability. For example, Rwanda’s CBHI operates under a highly centralized and mandatory enrollment system with stronger administrative capacity and enforcement mechanisms, which reduces variability in implementation and improves adherence to targeting rules. In contrast, Ethiopia’s more decentralized and community-driven approach relies heavily on local committees, increasing flexibility but also creating space for subjective interpretation, inconsistency, and potential favoritism. Similarly, Ghana’s NHIS benefits from a more formalized insurance infrastructure and urbanized population structure, which differs from Ethiopia’s predominantly rural and agrarian setting where livelihoods are more seasonal and difficult to classify using fixed asset-based measures. These differences suggest that while the principle of perceived fairness is consistent across settings, the feasibility and performance of targeting approaches are highly context-dependent [11]. Therefore, enhancing transparency, communication, and opportunities for community feedback is essential to transform technical classification into a socially validated process. Participants’ suggestions for refining the stratification framework—such as introducing a four-tier system to capture the gradient between extreme poverty and moderate means—reflect constructive engagement rather than opposition. This adaptive feedback indicates that the reform has already stimulated deliberation about fairness and social differentiation at the community level, a desirable outcome for participatory governance at community level. Addressing these practical issues—through regular review of proxies, participatory validation, and accessible grievance mechanisms—can enhance both accuracy and perceived justice. Importantly, such refinements are feasible and incremental, suggesting that the concerns raised are not fundamental flaws but rather manageable implementation challenges that can be resolved through iterative learning.

### 4.4. Governance, accountability, and sustaining trust

The study also highlighted governance-related challenges, including occasional favoritism by village committees, weak complaint-handling mechanisms, and limited capacity to reach wealthier households during awareness campaigns. These issues, while modest, have broader implications for the credibility and accountability of decentralized health financing systems. Evidence from other contexts shows that transparent communication, procedural fairness, and timely grievance redress are decisive for maintaining the social contract underpinning community-based insurance [26]. Establishing systematic community feedback platforms, participatory audits, and periodic performance reviews could strengthen vertical accountability and prevent the erosion of trust that often follows early program enthusiasm. From a policy perspective, these findings point to the critical interplay between technical design and institutional capacity. Even the most equitable financing formula can fail if administrative systems lack transparency or responsiveness. Conversely, transparent and participatory implementation can compensate for technical imperfections by fostering perceived fairness and public trust and legitimacy of the program [10,27]. Hence, the success of the differentiated premium model hinges less on the precision of asset proxies and more on the quality of governance surrounding its application.

### 4.5. Balancing simplicity, inclusiveness, and administrative feasibility

The argument among participants regarding the three-tier versus four-tier classification system reveals a deeper trade-off between simplicity and inclusiveness. While additional tiers could improve differentiation and perceived fairness, they also increase administrative complexity and the potential for disputes. Policymakers must therefore strike a balance between the precision of socio-economic targeting and the capacity of local structures to implement it consistently. Adaptive management—where classification frameworks evolve through empirical feedback and learning—offers a practical pathway to resolve this tension. Similarly, the reform’s success will depend on its ability to maintain inclusiveness without deterring participation from wealthier households. The pilot’s early evidence suggests that the differentiated contribution model can indeed mobilize higher contributions from the better-off without reducing their willingness to participate. However, maintaining this balance will require continuous engagement and assurance that contributions are used efficiently and transparently. Without this, even high initial participation rates could decline over time—a phenomenon observed in other CBHI programs when contributors perceive inequitable benefit distribution or weak accountability [28].

## 5. Conclusions

This early-phase assessment, conducted immediately following the introduction of the socio-economic-based premium contribution system for CBHI in Ethiopia, demonstrated an overall positive reception and acceptance among both community members and scheme implementers. The reform was broadly perceived as a fair, relevant, and contextually appropriate innovation that aligns premium contributions with households’ ability to pay. Participants viewed the asset-based, community-led classification process as an equitable approach that reflects existing norms of solidarity and mutual support, where better-off households are socially expected to contribute toward the welfare of poorer families. The participatory nature of the stratification process enhanced its legitimacy, ownership, and perceived fairness, fostering early trust in the scheme. However, while the principle of socio-economic stratification was widely acknowledged as legitimate and socially just, its implementation presented notable operational and ethical challenges. Misclassification of households, favoritism, inconsistent application of criteria, and limited communication were frequently reported. Inadequate grievance redress mechanisms and weak technical capacity at local levels further constrained the credibility of the classification process. Participants emphasized that to achieve genuine equity, classification should extend beyond visible assets to account for livelihood realities, income variability, and household vulnerability. Sustaining community trust and achieving long-term acceptance will therefore require improvements in transparency, procedural fairness, and institutional support.

## 6. Policy and practice implications

This study provides important insights into how Ethiopia’s differentiated CBHI premium reform can be strengthened for effective implementation, equity, and long-term sustainability. The findings show that while the sliding-scale model is broadly accepted, its success depends less on its technical design and more on how it is implemented, governed, and experienced by communities. Accordingly, the implications below are organized into practice, policy, and long-term sustainability considerations.

### 6.1. Practice implications

The findings highlight that the effectiveness of Ethiopia’s socio-economic-based CBHI premium system depends primarily on implementation quality at the local level rather than on the design of the premium model itself. A key practical implication is the need to strengthen the accuracy, consistency, and transparency of household classification processes. This can be achieved through the use of standardized but context-sensitive classification guidelines that better reflect the multidimensional and dynamic nature of rural and urban livelihoods. To reduce misclassification and perceived unfairness, community-level implementation structures and committees require sustained capacity strengthening. This includes regular training for classification committees, provision of clear operational manuals, and routine supportive supervision from district health offices. Standardization of procedures across villages is particularly important to minimize variation, subjective judgment, and potential favoritism in household categorization.

Grievance redress mechanisms also need to be made more functional, visible, and accessible. Practically, this can be implemented through structured appeal sessions at kebele (village) level, simple and low-cost complaint channels (such as suggestion boxes and designated focal persons at health facilities), and scheduled community review forums at village and PHCU levels where households can request reassessment of their classification. Importantly, these mechanisms should include clear response timelines and documentation procedures to ensure accountability and procedural fairness. Community engagement should be embedded within existing local CBHI governance structures. Health extension workers, kebele leaders, women’s associations, and community development groups can be systematically engaged to communicate classification criteria, explain premium structures, and facilitate dialogue with households. Public display and community validation of provisional household lists prior to finalization can further enhance transparency and reduce perceptions of bias.

### 6.2. Policy implications

At the policy level, the findings suggest the need to refine Ethiopia’s asset-based targeting approach to better capture real socio-economic conditions. Current indicators should be complemented with additional measures reflecting income and asset variability, and livelihood instability, particularly in rural and informal settings where welfare status is fluid and context-dependent. National CBHI guidelines should be revised to standardize classification principles while allowing controlled contextual adaptation at district level. This would improve national consistency, reduce subjective interpretation, and strengthen equity and transparency in implementation.

A further policy priority is institutionalizing grievance redress and accountability systems within CBHI governance structures at the primary level. This requires formal recognition of appeal mechanisms within PHCU and district health offices and clear accountability pathways for resolving classification disputes and other issues. Such institutionalization would strengthen procedural justice and reinforce legitimacy and public trust of the system. Collaboration between health, agriculture, in land revenue offices and social protection sectors can improve the reliability of socio-economic data and align CBHI targeting with broader national poverty identification systems. This would enhance efficiency, reduce duplication, and improve consistency across social programs. In addition, policy should introduce flexibility in premium collection modalities-aligning payment schedules with post-harvest periods would improve affordability and reduce dropout.

### 6.3. Long-term sustainability implications

The findings indicate that the long-term sustainability of Ethiopia’s differentiated CBHI reform depends more on sustained trust, perceived fairness, and visible service improvements than on the technical design of the premium model alone. Although initial acceptance of the sliding-scale approach is positive, it remains conditional and may weaken without continued evidence of tangible benefits. Sustaining participation requires that CBHI revenues are visibly translated into improved service delivery. This includes ensuring consistent availability of essential medicines, reducing stock-outs, improving referral coordination, and strengthening provider responsiveness. These improvements can be supported through stronger supply chain management, routine supportive supervision, and facility-level performance monitoring with feedback to district health authorities. Transparency and accountability mechanisms are equally critical. Regular community engagement, participatory monitoring, social audits, and simple public reporting of CBHI fund use at kebele and district levels should be embedded into routine implementation. These mechanisms help strengthen trust and create continuous feedback between communities and the health system. The study also highlights the need for adaptive implementation. Socioeconomic classification criteria should be periodically reviewed to reflect changing local realities, particularly in rural settings where livelihoods are dynamic. Without such updates, misclassification risks may accumulate and erode perceived fairness and trust over time. Overall, sustainability depends on aligning technical reforms with strong implementation systems. A participatory, transparent, and context-responsive approach—supported by institutional capacity, intersectoral coordination, and continuous community engagement—is essential for equitable and durable health financing in Ethiopia. This reform also reflects Ethiopia’s alignment with the global UHC agenda, supported by institutions such as WHO and the World Bank, while maintaining domestic ownership. The sliding-scale model therefore represents a locally adapted equity reform whose long-term success will depend on sustained political commitment, governance strength, and effective local implementation

## Supporting information

S1 Appendix Open-ended discussion/interview guides. (DOCX)

S2 Appendix Details of the codes and quotations underlying this analysis. (RTF)

S3 Appendix Consolidated Criteria for Reporting Qualitative Research (COREQ). (PDF)

S4 Appendix Resources mobilized in 2016 compared to 2015. (DOCX)
