# Supplementary material for: Early community reactions and acceptance of the Sliding-Scale Community Based Health Insurance Scheme in Ethiopia: Qualitative Findings from the treatment arm
Source: PLoS One. 2026 Jul 22;21(7):e0353876. doi: 10.1371/journal.pone.0353876 (PMC13390935; doi:10.1371/journal.pone.0353876)
Supplement: S1 Appendix — (DOCX) [file pone.0353876.s001.docx]

SECTION H: QUALITATIVE TOOLS

**FGD participants’ characteristics recording format**

Region: ______________________District/Town:______________________, Kebele_____________ Date: _____________ Starting time: _____________ Ending time: ______________, Name of data collector/facilitator/modulator: **_____________________**Name of assistant: _________________Setting: ______________

| **Participant’s** | **Age** | **CBHI membership category**  **1.Indigent (non-payers)**  **2. Middle HHs**  **3.Higher HHs**  **4.Uncategorized HHs** | **Membership type**  **1.New**  **2.Renew** | **Educational status** | **Religion** | **Occupation/main job** |
| --- | --- | --- | --- | --- | --- | --- |
| P1 |  |  |  |  |  |  |
| P2 |  |  |  |  |  |  |
| P3 |  |  |  |  |  |  |
| P4 |  |  |  |  |  |  |
| P5 |  |  |  |  |  |  |
| P6 |  |  |  |  |  |  |
| P8 |  |  |  |  |  |  |
| P9 |  |  |  |  |  |  |
| P10 |  |  |  |  |  |  |

**Instructions**

- Welcome the participants
- Introduce yourself and your colleague
- Explain the purpose of the discussion (use consent information sheet)
- Explain procedure of the discussions and norms for the discussion (equal participation, respect each other’s idea, speak out loudly and one person speak at a time, there is no write and wrong idea, confidential to the group
- Ask permission to use voice recorder

**Discussion guide for FGDs**

1. Please tell me what do you think or your experiences about health insurance in this area?

- Probe:
  - How well people in your community are membership of the health insurance? Why/why not? Benefits and disadvantages of being a membership?
  - Participation in and acceptance of the health insurance to communities? Why? Why not?
- Which group of society is often participating and which are not participate? Why/why not? (Probe: in terms of economic classes? Poor rich?
- Why do some households do not participate in the program? What the barriers? ( finance, access to services etc, quality of services etc)
- What are people concerns and worries about insurance services?
- **Premium contribution**
  - - What do people think regarding CBHI premium contribution in your area (Probe: amounts of contributions, basis for determination of amount, fairness and equity, acceptability, affordability etc)?
    - Peoples’ willingness and ability to contribute the required amount?
    - System of contribution-amount per households?) Adequacy of the contribution? Why/why not? Which amount is fair and affordable?
    - What shall be done to improve community’s participation/enrollment into CBHI program?
    - To what extent households in who are members of the program renew their membership? Why/why not? What shall be done to improve renewal rate?

1. Please tell health care seeking practice of communities in this area? Why/why not? How do you being CBHI membership or not membership affected care seeking practice?
2. What do you think the access to health care (drugs, laboratory, and health care providers’ receptions) for insured people? What about non-insured people? Why? What are the challenges?
3. Do you think CBHI promotes women’s and children care seeking and access to medical care? How/why?
4. In general what shall be done to improve health insurance service and health care service for insurance members?
5. Anything you want to add or tell us which we did not discussed yet?

**Key informative interview respondent’s recording format**

Region: ______________________zone__________________District/Town:______________________, role/position:_______________, contact address:__________________________(Phone):

Date: _____________ Starting time: _____________ Ending time: ______________

Name of data collector/facilitator/modulator: _____________________

1. Please tell me your roles and responsibility in relation to CBHI services?
2. Please tell me status, and coverage of CBHI in your area/district? Why/why not? What is your overall assessment of the schemes?
3. Would you please reflect on the amount of contribution to the program to be a membership?

- Sufficiency/adequacy to cover medical costs? Why/why not? Why? How people see it?
- Fairness and transparency in the contributions including collection and refund?
- Contribution scheme-fixed amount for all households or sliding based on household economic status? Why/why not?
- Adequacy? Affordability and fairness or equity in contribution

1. How do you see community’s willingness to participate in CBHI? Why? What are the key barriers for participation?
2. Please tell me how communities perceive and experiences of the CBHI? (Probe: community acceptance, attitude, participation, retention?)
3. Please tell me why some households do not get enrolled into CBHI program? (Probe: reasons, willingness, ability to pay, perceptions, service access, service quality and access, benefit packages, satisfactions with the services etc.)
4. Why some households do not renew their membership?
5. Please tell me how CBHI contributed to or impacted health service provisions (quality, supplies)?
6. Please tell health care seeking practice of communities in this area? Why/why not? How do you being CBHI membership or not membership affected care seeking practice?
7. Do you think being CBHI membership promotes women’s and children care seeking and access to medical care? How/why?
8. For pilot districts-Views of new policy initiative-sliding scale contribution

- What do you think if the contribution bases on household economic status? Why/why not?
- What would be the challenge if the contribution scheme would be shifted according to household economic status? Why?
- How do you think we could overcome these challenge?
- What would be the potential benefits/advantage of such shifts? (to CBHI program such as resource mobilization/financial sustainability, to community?, quality of services, care seeking, women empowerment, access/coverage, equity)
- What shall be done to increase acceptance and community participation/membership in the new policy initiative?

1. Anything you want to add or tell us which we did not discussed yet?

Thank you very much for your time and participation!
