# Supplementary material for: Early community reactions and acceptance of the Sliding-Scale Community Based Health Insurance Scheme in Ethiopia: Qualitative Findings from the treatment arm
Source: PLoS One. 2026 Jul 22;21(7):e0353876. doi: 10.1371/journal.pone.0353876 (PMC13390935; doi:10.1371/journal.pone.0353876)
Supplement: S2 Appendix — (RTF) [file pone.0353876.s002.rtf]

Codes-quotations list
Code-Filter: All
______________________________________________________________________

HU:	merged file
File:	 [C:\Users\hp\Desktop\FID_Slide_scale_trainig stakeholders\Papers_publications\merged file.hpr7]
Edited by:	Super
Date/Time:	2025-11-27 22:25:03
______________________________________________________________________

Code:  {0-0}
______________________________________________________________________

Code: Adequacy of contribution_SS_CBHI: disproportionate premium increment and expense {3-0}

P11: KII _Town HO head_Treatment.docx - 11:4 [First, as compared to current ..]  (19:19)   (Super)
Codes:	[Adequacy of contribution_SS_CBHI: disproportionate premium increment and expense] [Adequacy of contribution_SS_CBHI: Growing inflation] 
No memos

First, as compared to current inflation and the service provided the amount of money collected from insurers was not enough and fair. Second, a similar amount of payment among both the wealthy and poor was not correct. 

P18: KII_CBHI focal_treatment.docx - 18:10 [The previous contribution syst..]  (17:17)   (Super)
Codes:	[Adequacy of contribution_SS_CBHI: disproportionate premium increment and expense] [Adequacy of contribution_SS_CBHI: flat rate inadequecy] 
No memos

The previous contribution system has so many problems; the first one is the premium amount was too few to purchase health inputs and to provide adequate service. Do you think 410 birr can purchase medicine, laboratory services and other inputs for the whole family members for one year? As one client repeatedly visits health institution, the money paid for the service might be used up within two or three visits. The price of medicine and laboratory reagents have been increased from time to time and now it is unthinkable to provide full health service by 410 birr for one family members. Forget about the household members, this amount of money cannot cover the health need of one individual patient.

P19: KII_Head of  health Office_treatment.docx - 19:5 [Till now, the amount of the co..]  (27:27)   (Super)
Codes:	[Adequacy of contribution_SS_CBHI: disproportionate premium increment and expense] 
No memos

Till now, the amount of the contribution and the services imbalanced. The money collected from households and the services provided have huge gaps. If we convert the services to money and compare it with amount of contributions, the services provided are greater by a large percentage.

______________________________________________________________________

Code: Adequacy of contribution_SS_CBHI: flat rate inadequecy {1-0}

P18: KII_CBHI focal_ treatment.docx - 18:10 [The previous contribution syst..]  (17:17)   (Super)
Codes:	[Adequacy of contribution_SS_CBHI: disproportionate premium increment and expense] [Adequacy of contribution_SS_CBHI: flat rate inadequecy] 
No memos

The previous contribution system has so many problems; the first one is the premium amount was too few to purchase health inputs and to provide adequate service. Do you think 410 birr can purchase medicine, laboratory services and other inputs for the whole family members for one year? As one client repeatedly visits health institution, the money paid for the service might be used up within two or three visits. The price of medicine and laboratory reagents have been increased from time to time and now it is unthinkable to provide full health service by 410 birr for one family members. Forget about the household members, this amount of money cannot cover the health need of one individual patient.

______________________________________________________________________

Code: Adequacy of contribution_SS_CBHI: Growing claim {0-0}
______________________________________________________________________

Code: Adequacy of contribution_SS_CBHI: growing expense {0-0}
______________________________________________________________________

Code: Adequacy of contribution_SS_CBHI: Growing inflation {1-0}

P11: KII _Town HO head_Treatment.docx - 11:4 [First, as compared to current ..]  (19:19)   (Super)
Codes:	[Adequacy of contribution_SS_CBHI: disproportionate premium increment and expense] [Adequacy of contribution_SS_CBHI: Growing inflation] 
No memos

First, as compared to current inflation and the service provided the amount of money collected from insurers was not enough and fair. Second, a similar amount of payment among both the wealthy and poor was not correct. 

______________________________________________________________________

Code: Adequacy of contribution_SS_CBHI: growing service cost {0-0}
______________________________________________________________________

Code: Adequacy of contribution_SS_CBHI: high service cost {1-0}

P14: KII HEW_2024_.docx - 14:2 [The community contributed a sm..]  (12:12)   (Super)
Codes:	[Adequacy of contribution_SS_CBHI: high service cost] 
No memos

The community contributed a small amount of money and they are served up to inpatient services. Based on this, it means that when a person who is not a member of health insurance becomes ill, he/she would be served by selling his/her livestock or land. Therefore, this contribution is small. For example, how much does syrup cost us today if we go to the pharmacy? So the community is willing to join this health insurance membership that we will use our whole family when we are sick.

______________________________________________________________________

Code: Adequacy of contribution_SS_CBHI: imbalanced premium and expense {0-0}
______________________________________________________________________

Code: Adequacy of contribution_SS_CBHI: Inflation {0-0}
______________________________________________________________________

Code: Adequacy of contribution_SS_CBHI: medicine price {1-0}

P14: KII HEW_2024_.docx - 14:4 [For example, how much does syr..]  (12:12)   (Super)
Codes:	[Adequacy of contribution_SS_CBHI: medicine price] 
No memos

For example, how much does syrup cost us today if we go to the pharmacy? So the community is willing to join this health insurance membership that we will use our whole family when we are sick.

______________________________________________________________________

Code: Adequacy of contribution_SS_CBHI: service vs premium {1-0}

P16: KII__HEW_.docx - 16:1 [The contribution of members of..]  (15:15)   (Super)
Codes:	[Adequacy of contribution_SS_CBHI: service vs premium] [Adequacy of sliding premium_SS_CBHI: imbalance of expense and premium] 
No memos

The contribution of members of community based health insurance is not enough to cover the cost of their treatment. Because if we compare private and public health facilities, the whole family is treated throughout the year with a one-time contribution from the public health facilities, while private health facilities cost up to birr 3,000 per person at a time.

______________________________________________________________________

Code: Adequacy of contribution_SS_CBHI: Sustainability concern {0-0}
______________________________________________________________________

Code: Adequacy of sliding premium_SS_CBHI: ability to pay vs adequecy {1-0}

P16: KII__HEW_.docx - 16:5 [Their (Community) contribution..]  (21:21)   (Super)
Codes:	[Adequacy of sliding premium_SS_CBHI: ability to pay vs adequecy] 
No memos

Their (Community) contribution for community based health insurance is not enough. But there is a problem with the ability of the community to pay, so we look at their difficulty (due to famine and falling chat prices) and collect money from them.

______________________________________________________________________

Code: Adequacy of sliding premium_SS_CBHI: consider healthcare expenses {1-0}

P12: KII CBHI focal_TREATMENT TW.docx - 12:3 [In this regard, I think a stud..]  (15:15)   (Super)
Codes:	[Adequacy of sliding premium_SS_CBHI: consider healthcare expenses] [Adequacy of sliding premium_SS_CBHI: gap of premium and inflation] 
No memos

In this regard, I think a study of the market, payment, and health services should be conducted because the prices of various laboratory examinations and medicines are increasing from time to time. Therefore, I do not think that the CBHI will continue with its current payment amount unless the health care and payment situation are thoroughly investigated and improvements are made according to the market situation. Generally, although this amount of settlement is good as a starting point, it needs to be improved in the future.

______________________________________________________________________

Code: Adequacy of sliding premium_SS_CBHI: enough budget {1-0}

P18: KII_CBHI focal_treatment.docx - 18:5 [However, the new initiative st..]  (11:11)   (Super)
Codes:	[Adequacy of sliding premium_SS_CBHI: enough budget] 
No memos

However, the new initiative started this year [sliding scale] seems a good system to collect enough amount of budget to provide adequate service for our clients.

______________________________________________________________________

Code: Adequacy of sliding premium_SS_CBHI: financial strength {1-0}

P19: KII_Head of  health Office_treatment.docx - 19:6 [I believe the sliding scale he..]  (27:27)   (Super)
Codes:	[Adequacy of sliding premium_SS_CBHI: financial strength] [Sliding scale benifits_SS_CBHI: service improvement] 
No memos

 I believe the sliding scale helps to close the large gaps of contribution amount and services provided. The money collected using fat scale not serve for more than a quarter. Thus, the sliding scale help us to solve the shortage of health services due to lack of money. 

______________________________________________________________________

Code: Adequacy of sliding premium_SS_CBHI: gap of premium and inflation {1-0}

P12: KII CBHI focal_TREATMENT TW.docx - 12:3 [In this regard, I think a stud..]  (15:15)   (Super)
Codes:	[Adequacy of sliding premium_SS_CBHI: consider healthcare expenses] [Adequacy of sliding premium_SS_CBHI: gap of premium and inflation] 
No memos

In this regard, I think a study of the market, payment, and health services should be conducted because the prices of various laboratory examinations and medicines are increasing from time to time. Therefore, I do not think that the CBHI will continue with its current payment amount unless the health care and payment situation are thoroughly investigated and improvements are made according to the market situation. Generally, although this amount of settlement is good as a starting point, it needs to be improved in the future.

______________________________________________________________________

Code: Adequacy of sliding premium_SS_CBHI: growing inflation {1-0}

P13: KII  _HC-Head _ Ttreatment.docx - 13:3 [If we compare it with the serv..]  (15:15)   (Super)
Codes:	[Adequacy of sliding premium_SS_CBHI: growing inflation] [Adequacy of sliding premium_SS_CBHI: imbalance of expense and premium] 
No memos

If we compare it with the services they received, the membership contribution is not enough and the membership contribution should increase. Because although the current contribution is better, the cost of living as well as medicines is increasing from time to time and it is not enough. Even most of the community believes that the contribution is not enough. For example, they know how much they pay for a one-time service while they are sick in a hospital or clinic, so they know that the contribution is not enough by comparison.

______________________________________________________________________

Code: Adequacy of sliding premium_SS_CBHI: high healthcare seeking and consumption {1-0}

P19: KII_Head of  health Office_treatment.docx - 19:8 [Currently, the health seeking ..]  (27:27)   (Super)
Codes:	[Adequacy of sliding premium_SS_CBHI: high healthcare seeking and consumption] 
No memos

Currently, the health seeking behavior of the people is increasing. The disease burden is also increasing. But the amount of money collected from households for CBHI membership is too small and can't cover the services they utilize. 

______________________________________________________________________

Code: Adequacy of sliding premium_SS_CBHI: imbalance of expense and premium {4-0}

P13: KII_HC-Head _ Ttreatment.docx - 13:3 [If we compare it with the serv..]  (15:15)   (Super)
Codes:	[Adequacy of sliding premium_SS_CBHI: growing inflation] [Adequacy of sliding premium_SS_CBHI: imbalance of expense and premium] 
No memos

If we compare it with the services they received, the membership contribution is not enough and the membership contribution should increase. Because although the current contribution is better, the cost of living as well as medicines is increasing from time to time and it is not enough. Even most of the community believes that the contribution is not enough. For example, they know how much they pay for a one-time service while they are sick in a hospital or clinic, so they know that the contribution is not enough by comparison.

P16: KII__HEW_.docx - 16:1 [The contribution of members of..]  (15:15)   (Super)
Codes:	[Adequacy of contribution_SS_CBHI: service vs premium] [Adequacy of sliding premium_SS_CBHI: imbalance of expense and premium] 
No memos

The contribution of members of community based health insurance is not enough to cover the cost of their treatment. Because if we compare private and public health facilities, the whole family is treated throughout the year with a one-time contribution from the public health facilities, while private health facilities cost up to birr 3,000 per person at a time.

P17: KII_CBHI Focal_treatment.docx - 17:2 [When we compare the amount of ..]  (19:19)   (Super)
Codes:	[Adequacy of sliding premium_SS_CBHI: imbalance of expense and premium] 
No memos

When we compare the amount of money contributed by households and the services they utilize, the amount of money contributed by households are not sufficient to cover the health services utilized by their family members.

P19: KII_Head of  health Office_treatment.docx - 19:7 [Nowadays, the cost of medicine..]  (27:27)   (Super)
Codes:	[Adequacy of sliding premium_SS_CBHI: imbalance of expense and premium] 
No memos

Nowadays, the cost of medicines are too high and hence, the amount of contribution collected from household is not adequate. For instance, one person may consume up to 50,000 to 80,000birr at hospital which is quite far from the amount of premium collected from them. Because of this, we face shortage to reimburse the money for hospitals and model pharmacies. 

______________________________________________________________________

Code: Adequecy_SS_CBHI: Believe government must subside {0-0}
______________________________________________________________________

Code: Adequecy_SS_CBHI: Flat rate deficiency {1-0}

P18: KII_CBHI focal_ treatment.docx - 18:4 [To be honest, the former contr..]  (11:11)   (Super)
Codes:	[Adequecy_SS_CBHI: Flat rate deficiency] 
No memos

To be honest, the former contribution system [flat rate] and the amount of premium could not fully support us to provide the expected level of service for our clients. For example, the amount collected last year was about 4 million birr, which has 9 million difference with this year's contribution.

______________________________________________________________________

Code: Affordability of sliding premium_SS_CBH: align with service quality {1-0}

P20: KII_Kebele manager_ treatment.docx - 20:16 [The amount of premium is not t..]  (23:23)   (Super)
Codes:	[Affordability of sliding premium_SS_CBH: align with service quality] 
No memos

The amount of premium is not that much difficult for our community; even they are ready to pay more than this if the service provision is improved

______________________________________________________________________

Code: Affordability of sliding premium_SS_CBH: good awareness of infilation {1-0}

P20: KII_Kebele manager_treatment.docx - 20:8 [This year the amount of paymen..]  (9:9)   (Super)
Codes:	[Affordability of sliding premium_SS_CBH: good awareness of infilation] 
No memos

This year the amount of payment significantly increased; our community knows that the price of medicine if being increased from time to time like other goods on the market. Therefore, they have no question on this increment rather they have question on unavailability of medicine in the health facilities; they are ready to pay the required amount if health facilities can provide all required medicine for them. 

______________________________________________________________________

Code: Affordability of sliding premium_SS_CBH: unexpected increment {1-0}

P16: KII__HEW_.docx - 16:2 [Most of the community says it'..]  (15:15)   (Super)
Codes:	[Affordability of sliding premium_SS_CBH: unexpected increment] 
No memos

Most of the community says it's enough, and a few of them say it's too hard for us. Because we paid a little money last year and it added to us this year, why? They asked. They will understand if we point out that the contribution has increased over last year as the purchasing of medicines has increased.

______________________________________________________________________

Code: Attitude SS_CBHI: favors flat rate {0-0}~
______________________________________________________________________

Code: Attitude SS_CBHI: positive and beneficial {3-0}

P 4: FGD_MEN_ treatment.docx - 4:15 [Our community's income is main..]  (132:132)   (Super)
Codes:	[Attitude SS_CBHI: positive and beneficial] [Premium contribution challenge: droped chat price] 
No memos

Our community's income is mainly based on chat and now the price of chat has dropped and it is become challenge to pay. That's why some people say it's hard for us to contribute the insurance membership fee. How do I become the high payer when the committee ranks to high, medium and low payer households? Few people mention that I have not reached this level. However, the community knows that health insurance is beneficial and believes in it and becomes an insurance member.

P 7: FGDs_Men_ treatment.docx - 7:18 [The community will assume that..]  (144:144)   (Super)
Codes:	[Attitude SS_CBHI: positive and beneficial] [Attitude SS_CBHI: Supportive and recomend] [Stratification: fair] 
No memos

The community will assume that is balanced. As one, this person is one powerful, one is powerless. When it comes to the assessment group, they measured how much wealth he had and how much this person had. They measured it on three levels, one high, one medium, and the last, low. That's how they measured it. That evaluation of this thing is pretty nice.

P 9: KII_Cluster_CBHI Desk KII.docx - 9:11 [Until now, the same sliding sc..]  (36:36)   (Super)
Codes:	[Attitude SS_CBHI: positive and beneficial] 
No memos

Until now, the same sliding scale is being done at the pilot level, so it has not brought people's capacity. We know how to balance people, they have been studied in the right way on the sliding scale, and now they have been paid a lot of money for that thing. There are many calculations. Since it was built with all these things in mind, if the society can't contribute to that capacity, if people can't contribute and benefit from the service according to their capacity, unless CBHI misses the purpose for which it was established. He contributes according to his ability, but whenever wants the treatment, get the treatment according to the contract. It is basic to contribute according to the economic capacity.

______________________________________________________________________

Code: Attitude SS_CBHI: positive and happy to contribute {1-0}

P 4: FGD_MEN_treatment.docx - 4:16 [ur community has willingness t..]  (134:134)   (Super)
Codes:	[Attitude SS_CBHI: positive and happy to contribute] [Premium contribution challenge: droped chat price] 
No memos

ur community has willingness to pay but it is challenging to pay due to undervalued chat price. So, the community's economy depends on chat revenue. However, we are being treated in many rounds with a one-time contribution. So it means our community is happy to contribute.

______________________________________________________________________

Code: Attitude SS_CBHI: positive, lestds the government continue it {5-0}~

P 2: FGD_Men__TREATMENT.docx - 2:6 [Then we learned the importance..]  (121:121)   (Super)
Codes:	[Attitude SS_CBHI: positive, lestds the government continue it] [PB_CBHI: No out of pocket expenditure] 
No memos

Then we learned the importance of insurance and recommended others to be members of insurance. We all are enrolled in membership. We are happy with the current payment classification that considers the lower income as a low income, the middle income as his middle income, and the higher income as his income. We are happy, that we cannot pay from our pocket when we are sick but using our membership ID. 

P 2: FGD_Men___TREATMENT.docx - 2:9 [currently we have a member of ..]  (122:122)   (Super)
Codes:	[Attitude SS_CBHI: positive, lestds the government continue it] [PB_CBHI:Service satisfaction] 
No memos

 currently we have a member of insurance and we treated using this insurance card when we ailed. The government profoundly supports this in different aspects because health insurance payment is based on an individual's capacity to pay both for lower and higher income. So I am mentally satisfied with it. Indeed, the government does not belong to only one individual but to all the country's individuals. But except for some individuals who ignored themselves, insurance has an important in a different aspect because in the past we carried patients using local beds (qareeza), but now, we can call for an Ambulance using call service numbers, by expressing the case that happened then the patient is immediately taken to the hospital for survive. Specially for laboring mothers, if possible it can be handled here (woreda) or referred to Dabra-birhane hospital. So we are very happy.

P 2: FGD_Men__TREATMENT.docx - 2:12 [In the past, we resisted being..]  (128:128)   (Super)
Codes:	[Attitude SS_CBHI: positive, lestds the government continue it] [PB_CBHI: No out of pocket payment] 
No memos

In the past, we resisted being members of health insurance without knowing its importance. For a long time, I did not understand the benefits of it, but last summer I fully understood health insurance benefits because if you are a member of it, you can get services like for registration card, and drugs from health facilities without payment. If the drugs are not available at health facilities there is a community service pharmacy in the town. You can get drugs from there without payment. But if you are not, you may buy drugs and everything from a private clinic with a huge amount of money. The second point is in the previous all individuals paid the same amount of money 410 birr. But now contribution is considered the capacity of individuals to pay which is a very important measurement taken by the government. 

P 2: FGD_Men__.docx - 2:20 [This is the barriers community..]  (143:144)   (Super)
Codes:	[Attitude SS_CBHI: positive, lestds the government continue it] [Premium collection challenge: similar time with tax collection] [Weakness and challenges SS-CBHI implementation : missclasification] 
No memos

This is the barriers community and farmers' barriers to membership. In another way, the classification of payment that was categorized by the government is very good in classifying to lower, middle, and higher. It is based on their income, the presence of land for irrigation or not, the types of their house condition, and others that are very nice.  However, during classification, there was a mistake in classification. For example, those have to be categorized under poor/lower level classified under middle level and vs verses. But there were no problems with upper-class categorization. So it is better to correct those misclassification. 
The other suggestion that will be improved is the price of annual contribution. It is better if it reconsiders the paying capacity of individuals.  Since the health insurance contribution is paid with other expenses like taxation, the total it costs is above the price of a cattle (notify).   So it is difficult to pay in this situation. So I suggest some adjustments to the classification payment contribution.

P 2: FGD_Men__TREATMENT.docx - 2:31 [Regarding the payment scheme i..]  (168:169)   (Super)
Codes:	[Attitude SS_CBHI: positive, lestds the government continue it] [strength of CBHI_SS implementation: government covers for the poor] [strength of CBHI_SS implementation: stratification is income based] [Weakness and challenges SS-CBHI implementation : missclasification] 
No memos

 Regarding the payment scheme it was categorized into three levels. The criteria that they used for classification are good. especially the criteria for high payers is good. but in rare cases, there was misclassification simply observing Qororroo Manaa and the clothes he wears, classified under higher payer. In this area, it needs correction. It is better if it is re-evaluated. As mentioned in this discussion the government slide scale is a very nice job that brought. There is a person who is unable to afford even the minimum payment of seven hundred birr. But the action taken by the government that everyone including the poor to be treated using health insurance is very good. As a farmer, those poor are with us. So we are very happy that the poor can get treatment by paying the starting payment. You know, if such types of service are not available, the poor person may borrow for treatment. If he/she does not have money to return the money by selling his land and becomes landless and exposed to hunger then dies. So it's a great job done government. It is nice. 
P5: The sliding scale (addaan baasuu) is very important because the poor may die due to lack of money for treatment. The government plan was very good and categorizing is also good. let the government keep up it. in our Garee there are no problems with categorizing but in others, there may be problems in properly categorizing.

______________________________________________________________________

Code: Attitude SS_CBHI: supportive {5-0}

P 9: KII_ _Cluster_CBHI Desk KII.docx - 9:5 [CBHI was just established with..]  (30:30)   (Super)
Codes:	[Attitude SS_CBHI: supportive] 
No memos

CBHI was just established with one principle, people contribute according to their ability, but when need treatment, they will get the treatment according to the contract. It should be paid according to their capacity. Each society has a different economic status according to capacity.

P 9: KII__Cluster_CBHI Desk KII.docx - 9:9 [Therefore, if we have to contr..]  (34:34)   (Super)
Codes:	[Attitude SS_CBHI: supportive] 
No memos

Therefore, if we have to contribute according to our ability and benefit from the service, each family should contribute according to the required level. Therefore, I say that the contribution that is being collected now is almost still not enough. 

P 9: KII_ Cluster_CBHI Desk KII.docx - 9:10 [the sliding scale, which was s..]  (34:34)   (Super)
Codes:	[Attitude SS_CBHI: supportive] 
No memos

the sliding scale, which was started with the pilot, may be applied to everyone, so it will create a way for your people to contribute according to their ability.

P 9: KII_ Cluster_CBHI Desk KII.docx - 9:12 [In the current situation, norm..]  (38:38)   (Super)
Codes:	[Attitude SS_CBHI: supportive] 
No memos

 In the current situation, normally when we prepare the first sliding scale, in terms of purpose, in terms of benefits, in terms of the way in which it is made, I believe that it can confirm the purpose of CBHI very well and continuously. 

P 9: KII_ Cluster_CBHI Desk KII.docx - 9:15 [t's necessary to classify thes..]  (38:38)   (Super)
Codes:	[Attitude SS_CBHI: supportive] [implementation challenge: budget shortage] 
No memos

t's necessary to classify these people before the work is done. Who should on the high, who should on the medium, and who should on the lower. But when I got into the practice after already classified, things were difficult. We don't have money at this stage, so it was a bit of a burden to carry it out. we had a slight problem with it. I think it will be fixed continuously

______________________________________________________________________

Code: Attitude SS_CBHI: Supportive and recomend {5-0}~

P 6: FGdD_Female_ treatment.docx - 6:1 [In the beginning, we heard tha..]  (125:125)   (Super)
Codes:	[Attitude SS_CBHI: Supportive and recomend] [PB_CBHI:  inclussion of all family memebers for the service] [PB_CBHI: No out of pocket payment] [Premium contribution: fair: equivalent with  one time service at private] 
No memos

In the beginning,  we heard that health insurance be served to get health care without payment. They told us once we paid it would be servi for ourselves and our family. we paid for it and we received receipts based on our payment. And that individual who cannot able to pay can get a free membership. There are three different categories of payment schemes. So the health insurance is very important. The amount we pay for a membership that is served for a year for us and our children may be equivalent to the payment we pay at one visit of service at a private clinic.

P 7: FGDs_Men__ treatment.docx - 7:13 [The price is decisive and it i..]  (134:134)   (Super)
Codes:	[Attitude SS_CBHI: Supportive and recomend] 
No memos

The price is decisive and it is fair. We ranked it as low, medium, high. We decided our reasonable price. We grouped it according to the capacity of the man, the capacity of the person. the strength of the man, low and high, medium. That's how we're giving receipt.

P 7: FGDs_Men__ treatment.docx - 7:18 [The community will assume that..]  (144:144)   (Super)
Codes:	[Attitude SS_CBHI: positive and beneficial] [Attitude SS_CBHI: Supportive and recomend] [Stratification: fair] 
No memos

The community will assume that is balanced. As one, this person is one powerful, one is powerless. When it comes to the assessment group, they measured how much wealth he had and how much this person had. They measured it on three levels, one high, one medium, and the last, low. That's how they measured it. That evaluation of this thing is pretty nice.

P 9: KII_ Cluster_CBHI Desk KII.docx - 9:6 [Because if the poor, the middl..]  (30:30)   (Super)
Codes:	[Attitude SS_CBHI: Supportive and recomend] 
No memos

Because if the poor, the middle-class, and the high-class contribute equally, we can't say that we have proven justice. But if everyone is allowed to contribute according to the economic status they have, the first principle is that those who have not are supported. I have an idea that if the sliding scale are applied, it is possible to ensure more fairness.

P 9: KII_ Cluster_CBHI Desk KII.docx - 9:7 [f all the members pay equally,..]  (32:32)   (Super)
Codes:	[Attitude SS_CBHI: Supportive and recomend] 
No memos

f all the members pay equally, maybe one of them has the ability to pay, then as I said earlier, we said that it is helping. Helping in principle is as example the younger class helps these older ones. This is a charity that now has a large amount of money. So, if we contribute equally, it is not fair. If CBHI needs to continue, we should help financially. So, if people contribute equally, firstly it's not fair, secondly, we the continuity of CBHI in doubts. Thirdly, people lose their sense of belonging. If I don't contribute to what I deserve, I think it matters. It means that I don't care if CBHI grows or not. For example, if someone always pays me, I don't feel anything, it's like that. Therefore, in order for one CBHI to be stand, the society and the health sector must also be. Therefore, since CBHI is a mechanism through which we support each other, if we make the same contribution so that there is no continuity. Second, it raises complain among people. I have an idea that how can a person who has this much capital contribute as much as I do, and that we will send CBH to an unwanted purpose.

______________________________________________________________________

Code: Attitude toward CBHI: good {1-0}

P 2: FGD_Men_TREATMENT.docx - 2:24 [Really, health insurance has a..]  (149:149)   (Super)
Codes:	[Attitude toward CBHI: good] [Poor reception of health workers:] [Service gap: delay referal process] 
No memos

Really, health insurance has a lot of benefits. In another way, my complaint was with health professionals. For example, we took to laboring mother to a health facility and they told us to wait until midnight and back again. But mother suffered and was near to death. The person on duty was already asleep and after many knocks on his door, he woke up. After many repeated requests to evaluate the mother, he came and tried to evaluate but never considered the mother's pain and screamed at me by saying 'get out from me' We also requested them to refer to other facilities if it is beyond their capacity. Finally, after many struggles, they referred it to Debrabihan Hospital. When we arrived at the hospital, the Debrabiran doctors shouting me “Why are you delayed until such kind the mother suffered?” they condescended to me. finally, they saved that mother. So insurance is very essential but what shall we do? Are the health workers abusing us? This is our major probl

______________________________________________________________________

Code: Attitude toward CBHI: good and eager to renew {1-0}~

P 6: FGdD_Female_treatment.docx - 6:5 [We cannot finshed the insuranc..]  (127:127)   (Super)
Codes:	[Attitude toward CBHI: good and eager to renew] [PB_CBHI:  referal service] [PB_CBHI: good pakage of service (admission)] [PB_CBHI: No out of pocket expenditure] 
No memos

We cannot finshed the insurance benefit by listing it within this limited time. At the start of insurance in the nation, most people fear to engage in insurance by saying our money would treat us. We do not want this membership card and call it a 'carton'.we can treat ourselves with no need from the government. However, after the community understood the advantages of health insurance, they immediately paid the annual premium and renewed the card within a given deadline and utilizing. Like fertilizer in its beginning most people take it forcibly we also had taken insurance. But now that we the farmers searching for health insurance like sugar because they are well aware of the importance of health insurance. We have been treated here but if it is beyond the health center we are referred to Dodola Hospital. During this referral service, we only paid for transportation and there is no anything we paid for the Dodola hospital. We never pay for admission, cards, and drugs. So the importance of health insurance is so many to list.  So we would like to thank the government for providing such services for us.

______________________________________________________________________

Code: Attitude toward CBHI: poor {0-0}~
______________________________________________________________________

Code: Attitude_SS_CBHI: poor service availability {1-0}

P20: KII_Kebele manager_treatment.docx - 20:2 [When we look at the community'..]  (5:5)   (Super)
Codes:	[Attitude_SS_CBHI: poor service availability] 
No memos

When we look at the community's attitude toward this CBHI program, there are both positive and negative attitudes in our community.  As you know, CBHI began many years ago and the majority of our community benefited from it; medicines were available before years; however, in recent years, both health centers and hospitals could not provide the expected level of services for our community; you cannot get the medicines you want from health centers and hospitals at this time. 

______________________________________________________________________

Code: Awareness creation campaign {3-0}

P 9: KII_ Cluster_CBHI Desk KII.docx - 9:22 [Well, it was the same as I sai..]  (50:50)   (Super)
Codes:	[Awareness creation campaign] 
No memos

Well, it was the same as I said before, now I can say that CBHI can sustained if we have to enable it to continue, the members have to renew. If members are not renewing, it means there is a problem. So, most of the time, looking at the current catchment of two thousand and sixteen, as I said before, it is hundred percent renewal, hundred percent new members. As I said, it is hundred percent in East Hararge. It's hundred percent on Maya City. So, what does this show? It means that the members who renew know the benefits and renew more. Seeing the renewal, the new members are learning from others, those who were running behind, those who were saying that it is useless, and those who were saying that it is useless, seeing that those existing members. Some of the awareness creation that we are doing for them is the situation that many people are coming here willingly. To bring them in, as I said earlier, you will work especially on creating awareness to bring new members. It is a condition of improving the quality of service in order to maintain it. The same will be evaluated and we now have a section called Provider. Periodic audit works, again comprehensive works. So, this is about readiness. How many health institutions are ready is evaluated every time, every six months. And what kind of status is it? These people are providing services to the society by buying the resources they need with the money they earn.

P 9: KII_Cluster_CBHI Desk KII.docx - 9:24 [I think that may be probably d..]  (52:52)   (Super)
Codes:	[Awareness creation campaign] [CBHI attiude: improving] 
No memos

 I think that may be probably depend on the understanding some households. Awareness creation is done systematically. But what they understand, their attitude and understanding towards CBHI may be low. But many members are understanding the importance of this matter

P 9: KII_ Cluster_CBHI Desk KII.docx - 9:42 [the main thing is to organize ..]  (78:78)   (Super)
Codes:	[Awareness creation campaign] 
No memos

the main thing is to organize the creation of awareness every time. It can be through different channels, it can be through the media, it can be through Montarbo, it can be through brochures, it is to expand the awareness network by using different channels. Therefore, if you feed the society every time when new things come, the society will actively participate in that matter. After that, I have an idea that they can play the role we want in this service.

______________________________________________________________________

Code: Barriers_SS_CBHI: attitude {1-0}

P16: KII__HEW_.docx - 16:10 [Regarding attitude some people..]  (25:25)   (Super)
Codes:	[Barriers_SS_CBHI: attitude] [Barriers_SS_CBHI: poor service] 
No memos

Regarding attitude some people when they go to the health center if there is a problem, they stand on that and get angry and don't pay. Even they said that when we get sick we will be treated with our own money.

______________________________________________________________________

Code: Barriers_SS_CBHI: attitude and awareness {1-0}

P16: KII__HEW.docx - 16:13 [I paid for community based hea..]  (29:29)   (Super)
Codes:	[Barriers_SS_CBHI: attitude and awareness] [Reasons for not becoming member_SS_CBHI: family health status] 
No memos

I paid for community based health insurance last year, but I didn't go to the health facility even for a single day last year because I didn't get sick. So there are people who say that I should not pay insurance this year because I paid it last year but not served.

______________________________________________________________________

Code: Barriers_SS_CBHI: awareness {1-0}

P13: KII _HC-Head _ Ttreatment.docx - 13:7 [One of the things that can be ..]  (23:23)   (Super)
Codes:	[Barriers_SS_CBHI: awareness] [Barriers_SS_CBHI: poor service] 
No memos

One of the things that can be a barrier to participation in community based health insurance is if there are not enough medicines, and if the professional does not provide the right services. From our experience, there is even an obstacle that a few people say that, we pay money for CBHI membership contribution but not served.

______________________________________________________________________

Code: Barriers_SS_CBHI: health workers behavior {2-0}

P18: KII_CBHI focal_treatment.docx - 18:20 [As the closest stakeholder and..]  (27:27)   (Super)
Codes:	[Barriers_SS_CBHI: health workers behavior] 
No memos

As the closest stakeholder and responsible office for CBHI related services, our community repeatedly visit our office and tell us that health workers do not respect them and give priority for non-members while providing services. To verify this report, we tried to observe service delivery process at different health facilities under our catchment. There were some issues that identified as problems; like disrespect from health workers, and considering CBHI members as if they visit the facility without getting illness.

P20: KII_Kebele manager_treatment.docx - 20:18 [Another problem that can be li..]  (23:23)   (Super)
Codes:	[Barriers_SS_CBHI: health workers behavior] [Service gap_SS_CBHI: Discrimination between CBI members and non-members] 
No memos

Another problem that can be listed as a barrier is not giving priority for CBHI members and disrespecting CBHI members; they [health workers] always give priority and also respect non-CBHI clients, they do not treat you equally with clients those who do not have CBHI membership ID. 

______________________________________________________________________

Code: Barriers_SS_CBHI: inability to pay {1-0}

P14: KII HEW_.docx - 14:12 [The only thing that is barrier..]  (24:24)   (Super)
Codes:	[Barriers_SS_CBHI: inability to pay] [Barriers_SS_CBHI: premium amount] 
No memos

The only thing that is barrier to health insurance is the payment and there is nothing that stops community from becoming a member. They want it, they want it very much. They have nothing but what they say is a little higher on this payment. This year, insurance premium contributions have increased. At one point, the insurance premium contributions increased to Birr 1,700, which caused a little disappointment

______________________________________________________________________

Code: Barriers_SS_CBHI: medicine unavailable {5-0}

P18: KII_CBHI focal_ treatment.docx - 18:15 [To some extent, the different ..]  (21:21)   (Super)
Codes:	[Barriers_SS_CBHI: medicine unavailable] [Barriers_SS_CBHI: service unavailable] 
No memos

To some extent, the different payment system from neighbouring s was challenged us during premium collection. Another major challenge that demotivate the community to participate in CBHI scheme was unavailability of medicines in the health facility; our community has tired off by unavailability of medicines in the health facilities, the give up to their membership when you tell them the medicine they want is not available. Many households express this issue when you ask them to pay for CBHI membership: what is the benefit of becoming a member if they are not getting the services they need.

P18: KII_CBHI focal_treatment.docx - 18:19 [You know it is impossible to a..]  (25:25)   (Super)
Codes:	[Barriers_SS_CBHI: medicine unavailable] [Reasons for not becoming member_SS_CBHI: medicine unavailablity] 
No memos

You know it is impossible to avail all medicines in all health facilities; our community my expect that they can get all the services they need at any time. When this expectation does not meet, they may dissatisfy with their membership and may decide not to renew their membership. Therefore, in my opinion, major factor that affect the level of community's participation in CBHI is affected by medicine unavailability. Even sometimes, you cannot get some types of medicine from EPSS and on the private market; at this time our client cannot find the medicine even from private pharmacies. 

P20: KII_Kebele manager_treatment.docx - 20:5 [In these recent years, this th..]  (5:5)   (Super)
Codes:	[Barriers_SS_CBHI: medicine unavailable] 
No memos

In these recent years, this thing [unavailability of medicine] pushes our community from becoming to CBHI members; they do not want to pay for CBHI; they ask as why they have to pay as far as they cannot get the medicines from health facilities. 

P20: KII_Kebele manager_treatment.docx - 20:6 [As I told you earlier, this pr..]  (7:7)   (Super)
Codes:	[Barriers_SS_CBHI: medicine unavailable] 
No memos

As I told you earlier, this program started before years and our community gradually learned the advantage of becoming member. However, they still complain about the unavailability of medicines form health facilities.

P20: KII_Kebele manager_treatment.docx - 20:17 [The main problem is poor servi..]  (23:23)   (Super)
Codes:	[Barriers_SS_CBHI: medicine unavailable] [Service gap_SS_CBHI: medicine availability] 
No memos

The main problem is poor service provision; especially they [health workers] order patients to buy medicine from private market at high cost. Our community does not want to buy medicine from private market once they become member of CBHI.

______________________________________________________________________

Code: Barriers_SS_CBHI: poor service {2-0}

P13: KII HC-Head _ Ttreatment.docx - 13:7 [One of the things that can be ..]  (23:23)   (Super)
Codes:	[Barriers_SS_CBHI: awareness] [Barriers_SS_CBHI: poor service] 
No memos

One of the things that can be a barrier to participation in community based health insurance is if there are not enough medicines, and if the professional does not provide the right services. From our experience, there is even an obstacle that a few people say that, we pay money for CBHI membership contribution but not served.

P16: KII__HEW_.docx - 16:10 [Regarding attitude some people..]  (25:25)   (Super)
Codes:	[Barriers_SS_CBHI: attitude] [Barriers_SS_CBHI: poor service] 
No memos

Regarding attitude some people when they go to the health center if there is a problem, they stand on that and get angry and don't pay. Even they said that when we get sick we will be treated with our own money.

______________________________________________________________________

Code: Barriers_SS_CBHI: premium amount {1-0}

P14: KII HEW_.docx - 14:12 [The only thing that is barrier..]  (24:24)   (Super)
Codes:	[Barriers_SS_CBHI: inability to pay] [Barriers_SS_CBHI: premium amount] 
No memos

The only thing that is barrier to health insurance is the payment and there is nothing that stops community from becoming a member. They want it, they want it very much. They have nothing but what they say is a little higher on this payment. This year, insurance premium contributions have increased. At one point, the insurance premium contributions increased to Birr 1,700, which caused a little disappointment

______________________________________________________________________

Code: Barriers_SS_CBHI: rumours and defamations {2-0}

P12: KII CBHI focal__TREATMENT TW.docx - 12:5 [As a barrier, there are some p..]  (21:21)   (Super)
Codes:	[Barriers_SS_CBHI: rumours and defamations] 
No memos

As a barrier, there are some people in the community who are not well-intentioned and even defame the organization due to a lack of clear information about CBHI

P12: KII CBHI focal__TREATMENT TW.docx - 12:6 [As a barrier, there are some p..]  (21:21)   (Super)
Codes:	[Barriers_SS_CBHI: rumours and defamations] 
No memos

As a barrier, there are some people in the community who are not well-intentioned and even defame the organization due to a lack of clear information about CBHI. Such people create bad feelings in the community and prevent the community from becoming members and from taking advantage of the CBHI

______________________________________________________________________

Code: Barriers_SS_CBHI: service unavailable {2-0}

P12: KII CBHI focal___TREATMENT TW.docx - 12:11 [The main reason they did not r..]  (29:29)   (Super)
Codes:	[Barriers_SS_CBHI: service unavailable] 
No memos

The main reason they did not renew their membership was a lack of service. According to them, they are opposed to renewing their membership because they cannot get the services they want from the public health institution. 

P18: KII_CBHI focal__ treatment.docx - 18:15 [To some extent, the different ..]  (21:21)   (Super)
Codes:	[Barriers_SS_CBHI: medicine unavailable] [Barriers_SS_CBHI: service unavailable] 
No memos

To some extent, the different payment system from neighbouring s was challenged us during premium collection. Another major challenge that demotivate the community to participate in CBHI scheme was unavailability of medicines in the health facility; our community has tired off by unavailability of medicines in the health facilities, the give up to their membership when you tell them the medicine they want is not available. Many households express this issue when you ask them to pay for CBHI membership: what is the benefit of becoming a member if they are not getting the services they need.

______________________________________________________________________

Code: Basis for sliding premium_SS_CBHI: common rural assests {1-0}

P20: KII_Kebele manager__treatment.docx - 20:10 [We started from owning land; a..]  (13:13)   (Super)
Codes:	[Basis for sliding premium_SS_CBHI: common rural assests] 
No memos

We started from owning land; additionally we counted their cattle, and estimated their annual production of different types of grains. In addition, we also considered the types of their home. In addition to these assets, we also considered if they have plants like [bargamo, and gravilla], because, these can also be converted to money. 

______________________________________________________________________

Code: Basis for sliding premium_SS_CBHI: health status and disability {1-0}

P12: KII CBHI focal___TREATMENT TW.docx - 12:20 [In addition, we classified peo..]  (41:41)   (Super)
Codes:	[Basis for sliding premium_SS_CBHI: health status and disability] 
No memos

In addition, we classified people who were dependent on others for various reasons such as illness and disability and had no income as poor in both rural and urban areas.

______________________________________________________________________

Code: Basis for sliding premium_SS_CBHI: rural_crops and land {1-0}

P12: KII CBHI focal___TREATMENT TW.docx - 12:18 [As a , we look at diff..]  (37:40)   (Super)
Codes:	[Basis for sliding premium_SS_CBHI: rural_crops and land] 
No memos

As a , we look at different properties in urban and rural areas. In rural areas, we look at locally known products such as teff, ox, cow, tractors, trees, and land area. For instance,
Ø	If they have three or more hectares of land and good yield, we classify them as High 
Ø	If they have four or more ox, or tractor, we classify them as High 
Ø	If they have three hectares or less of land and two ox, we classify them as medium

______________________________________________________________________

Code: Basis for sliding premium_SS_CBHI: urban_business {1-0}

P12: KII CBHI focal___TREATMENT TW.docx - 12:19 [At the urban level, we divided..]  (41:41)   (Super)
Codes:	[Basis for sliding premium_SS_CBHI: urban_business] 
No memos

At the urban level, we divided them into three categories according to their housing, cars, and shops

______________________________________________________________________

Code: Benifits_SS_CBHI: Access {1-0}

P15: KII PHCU Leader__TREATMENT TW.docx - 15:11 [In addition to providing quali..]  (28:28)   (Super)
Codes:	[Benifits_SS_CBHI: Access] [Impact_SS_CBH: commy health status] 
No memos

In addition to providing quality services as our health center, we have been providing health education every working day at health centers, schools, and public gatherings. As a result, our community has good health service-seeking behaviors. For example, at this health center, we provide delivery services to 70 to 100 mothers per month.

______________________________________________________________________

Code: Benifits_SS_CBHI: Access and equity {1-0}

P11: KII _Town HO head_Treatment.docx - 11:1 [BHI is very important in ensur..]  (9:9)   (Super)
Codes:	[Benifits_SS_CBHI: Access and equity] 
No memos

BHI is very important in ensuring equity and accessibility of health services. Especially health insurance is pivotal to ensuring equity for all people regardless of their economic status

______________________________________________________________________

Code: Benifits_SS_CBHI: Access to levels of care {0-0}
______________________________________________________________________

Code: Benifits_SS_CBHI: bridge the disparity in access {3-0}

P12: KII CBHI focal_TREATMENT TW.docx - 12:12 [Despite its shortcomings, the ..]  (31:31)   (Super)
Codes:	[Benifits_SS_CBHI: bridge the disparity in access] [Benifits_SS_CBHI: equal access] 
No memos

Despite its shortcomings, the CBHI has played an important role in delivering health services to the community, especially to the poor. Therefore, the CBHI plays an important role in maintaining equity and fairness in health services.

P13: KII HC-Head _ Ttreatment.docx - 13:8 [If the community based health ..]  (25:25)   (Super)
Codes:	[Benifits_SS_CBHI: bridge the disparity in access] [Benifits_SS_CBHI: service utilization] 
No memos

If the community based health insurance members gets a little sick, they go to the health fscility immediately for seeking care. Previously, if community members were not members of community based health insurance, they would have difficulty getting treated because they could not afford it. They also have good experience. They have good participation. They inspire each other to become members. They will even continue to renew membership.

P14: KII HEW__2024_.docx - 14:11 [In previous, he/she didn't get..]  (23:23)   (Super)
Codes:	[Benifits_SS_CBHI: bridge the disparity in access] 
No memos

In previous, he/she didn't get inpatient service , he/she didn't have anything to pay for, but now that insurance service has come, we have renewed it in time, taken a photo, treated him/her in hospital and there is a refund. Then there are people who come and say that the insurance contribution is very small.

______________________________________________________________________

Code: Benifits_SS_CBHI: catastrophic expenditure {2-0}

P11: KII _Town HO head_ Treatment.docx - 11:19 [There are significant numbers ..]  (41:41)   (Super)
Codes:	[Benifits_SS_CBHI: catastrophic expenditure] [Benifits_SS_CBHI: Risk sharing] 
No memos

There are significant numbers of insurance users for medical costs their costs were 20,000, 30,000, and 40,000 from a small amount collected from each individual. It has known health insurance that develops a culture of helping each other.

P14: KII HEW__2024_.docx - 14:3 [when a person who is not a mem..]  (12:12)   (Super)
Codes:	[Benifits_SS_CBHI: catastrophic expenditure] 
No memos

when a person who is not a member of health insurance becomes ill, he/she would be served by selling his/her livestock or land. Therefore, this contribution is small.

______________________________________________________________________

Code: Benifits_SS_CBHI: cheap price {1-0}

P16: KII__HEW_ _ _.docx - 16:8 [The community has good percept..]  (25:25)   (Super)
Codes:	[Benifits_SS_CBHI: cheap price] 
No memos

The community has good perception for community based health insurance. They even have good experience. They are willing to pay once for what the whole family uses at a cheap price.

______________________________________________________________________

Code: Benifits_SS_CBHI: cheap_free service {1-0}

P17: KII_CBHI Focal__ treatment.docx - 17:5 [They have also interest to bec..]  (21:21)   (Super)
Codes:	[Benifits_SS_CBHI: cheap_free service] [Benifits_SS_CBHI: Removes financial barrier] 
No memos

They have also interest to become member of CBHI. The community has been saying that if we convert the health services we utilize, one of our family member can even consume more than the money we pay for CBHI renewal/membership. For instance, last year, 58 insured people referred to black lion and received health care without additional payment. Thus, the community know the benefits of CBHI and has great interest.

______________________________________________________________________

Code: Benifits_SS_CBHI: empower women and children {1-0}

P12: KII CBHI focal___TREATMENT TW.docx - 12:13 [The advent of CBHI services ha..]  (33:33)   (Super)
Codes:	[Benifits_SS_CBHI: empower women and children] 
No memos

 The advent of CBHI services has ensured equal access to health services for all. It has given women and children, in particular, more access to health care than ever before. As is well known, in the past, women did not have equal access to health services as men. 

______________________________________________________________________

Code: Benifits_SS_CBHI: equal access {1-0}

P12: KII CBHI focal___TREATMENT TW.docx - 12:12 [Despite its shortcomings, the ..]  (31:31)   (Super)
Codes:	[Benifits_SS_CBHI: bridge the disparity in access] [Benifits_SS_CBHI: equal access] 
No memos

Despite its shortcomings, the CBHI has played an important role in delivering health services to the community, especially to the poor. Therefore, the CBHI plays an important role in maintaining equity and fairness in health services.

______________________________________________________________________

Code: Benifits_SS_CBHI: free service {2-0}

P14: KII HEW__2024_.docx - 14:10 [The willingness of the communi..]  (23:23)   (Super)
Codes:	[Benifits_SS_CBHI: free service] 
No memos

The willingness of the community to participate in health insurance is good. This is because some people have been treated free of charge because of their health insurance membership. How many people have been treated in my village now?

P20: KII_Kebele manager__treatment.docx - 20:7 [I am living with this communit..]  (7:7)   (Super)
Codes:	[Benifits_SS_CBHI: free service] [Benifits_SS_CBHI: Risk sharing] 
No memos

I am living with this community and I know their opinion about becoming member of CBHI; many of our community especially those ever admitted to the hospital praise CBHI program. Even though they may not get all medicines from hospital, they can tell you that they save from huge expenses due to their membership; especially they talk about bed and food services in the hospital. 

______________________________________________________________________

Code: Benifits_SS_CBHI: free services {1-0}

P20: KII_Kebele manager__treatment.docx - 20:23 [especially, those who are CBHI..]  (29:29)   (Super)
Codes:	[Benifits_SS_CBHI: free services] [Impact_SS_CBHI: health seeking behavior] 
No memos

especially, those who are CBHI members do not delay tom seek health service. Even though they [members] may not get all medicines from health center, still they get many services free of charge like card, some medicine, laboratory diagnosis, and bed services, which encourage them to visit health facility whenever they feel illness.  

______________________________________________________________________

Code: Benifits_SS_CBHI: improved medicine accessability {0-0}
______________________________________________________________________

Code: Benifits_SS_CBHI: new borns accomodation {1-0}

P17: KII_CBHI Focal__ treatment.docx - 17:19 [The two months renewal period ..]  (33:33)   (Super)
Codes:	[Benifits_SS_CBHI: new borns accomodation] 
No memos

The two months renewal period is not for newborns or additional child (daa'imman dabalataa iddoo biraa turanii gara maatii dhufan). The guideline also allow newborns to become member of CBHI at any time throughout the year. 

______________________________________________________________________

Code: Benifits_SS_CBHI: Removes financial barrier {3-0}

P14: KII HEW__2024_.docx - 14:15 [Why do they spend money? If so..]  (27:27)   (Super)
Codes:	[Benifits_SS_CBHI: Removes financial barrier] 
No memos

Why do they spend money? If someone gets sick, there is no going to the village to look for money. She/he puts her/his insurance card in pocket goes to health center and even for referral. They have good experience with this.

P15: KII PHCU Leader___TREATMENT TW.docx - 15:1 [Therefore, CBHI is as importan..]  (10:10)   (Super)
Codes:	[Benifits_SS_CBHI: Removes financial barrier] 
No memos

Therefore, CBHI is as important as the Edir because CBHI has ensured equal access to health services for both the rich and the poor.

P17: KII_CBHI Focal__ treatment.docx - 17:5 [They have also interest to bec..]  (21:21)   (Super)
Codes:	[Benifits_SS_CBHI: cheap_free service] [Benifits_SS_CBHI: Removes financial barrier] 
No memos

They have also interest to become member of CBHI. The community has been saying that if we convert the health services we utilize, one of our family member can even consume more than the money we pay for CBHI renewal/membership. For instance, last year, 58 insured people referred to black lion and received health care without additional payment. Thus, the community know the benefits of CBHI and has great interest.

______________________________________________________________________

Code: Benifits_SS_CBHI: Risk sharing {3-0}

P11: KII _Town HO head_ Treatment.docx - 11:3 [The CBHI insurance has many im..]  (18:18)   (Super)
Codes:	[Benifits_SS_CBHI: Risk sharing] 
No memos

The CBHI insurance has many important. The major importance is to prevent individuals from financial costs when health problems are faced. 

P11: KII _Town HO head_ Treatment.docx - 11:19 [There are significant numbers ..]  (41:41)   (Super)
Codes:	[Benifits_SS_CBHI: catastrophic expenditure] [Benifits_SS_CBHI: Risk sharing] 
No memos

There are significant numbers of insurance users for medical costs their costs were 20,000, 30,000, and 40,000 from a small amount collected from each individual. It has known health insurance that develops a culture of helping each other.

P20: KII_Kebele manager__treatment.docx - 20:7 [I am living with this communit..]  (7:7)   (Super)
Codes:	[Benifits_SS_CBHI: free service] [Benifits_SS_CBHI: Risk sharing] 
No memos

I am living with this community and I know their opinion about becoming member of CBHI; many of our community especially those ever admitted to the hospital praise CBHI program. Even though they may not get all medicines from hospital, they can tell you that they save from huge expenses due to their membership; especially they talk about bed and food services in the hospital. 

______________________________________________________________________

Code: Benifits_SS_CBHI: service utilization {1-0}

P13: KII _ _HC-Head _ Ttreatment.docx - 13:8 [If the community based health ..]  (25:25)   (Super)
Codes:	[Benifits_SS_CBHI: bridge the disparity in access] [Benifits_SS_CBHI: service utilization] 
No memos

If the community based health insurance members gets a little sick, they go to the health fscility immediately for seeking care. Previously, if community members were not members of community based health insurance, they would have difficulty getting treated because they could not afford it. They also have good experience. They have good participation. They inspire each other to become members. They will even continue to renew membership.

______________________________________________________________________

Code: Capitation pilot {0-0}
______________________________________________________________________

Code: catastrophic expense: {0-0}~
______________________________________________________________________

Code: CBHI _challenge: lack of understanding {1-0}

P 1: _FGD_WOMEN_TREATMENT_TW.docx - 1:7 [his means that anyone who has ..]  (97:97)   (Super)
Codes:	[CBHI _challenge: lack of understanding] [CBHI_challenge: not only professionals] [PB_CBHI: developed passion] [PB_CBHI: eager to renew] [Perceived benefits of CBHI: free service] 
No memos

his means that anyone who has seen and heard of its benefits is eager to renew their membership. For example, in the last year, a child of a relative of mine who is a member of CBHI was lying in bed for surgery at Tulu Bolo. The child was treated free of all service fees, including food. In the same ward, there was a man without CBHI being treated, and he was treated and discharged at great expense for similar services as that of a child of my relative. Since then, I have developed a passion for the CBHI. I also told people I knew about the benefits and made them interested. So in my opinion, it is a lack of understanding, but the problem of professionals alone does not prevent renewing the membership.

______________________________________________________________________

Code: CBHI attiude: improving {1-0}

P 9: KII_ __Cluster_CBHI Desk KII.docx - 9:24 [I think that may be probably d..]  (52:52)   (Super)
Codes:	[Awareness creation campaign] [CBHI attiude: improving] 
No memos

 I think that may be probably depend on the understanding some households. Awareness creation is done systematically. But what they understand, their attitude and understanding towards CBHI may be low. But many members are understanding the importance of this matter

______________________________________________________________________

Code: CBHI attiude: it is valued program {1-0}

P 6: FGdD_Female_ treatment.docx - 6:31 [So the community started to gi..]  (155:155)   (Super)
Codes:	[CBHI attiude: it is valued program] [weakness from community-  lack family member Photo] 
No memos

 So the community started to give priory even more than tax paying. But there is carelessness after paying annually not to bring photos for renewal. I have been treated for a year by paying 1710 birr at once. However, at one visit it may be requested 3000-4000 at once in a private clinic. Learning from such things and inflation makes people engaged in insurance. 

______________________________________________________________________

Code: CBHI CHALLENGE: High community expectations {1-0}

P 8: KII HEW_SCH__TREATMENT_TW.docx - 8:6 [there is a problem of expectat..]  (24:24)   (Super)
Codes:	[CBHI CHALLENGE: High community expectations] 
No memos

 there is a problem of expectations among the community. The problem is that when the community goes to the health center, they think that everything should be provided for them. This one is bothering us as a barrier.

______________________________________________________________________

Code: cbhi coverage: {0-0}
______________________________________________________________________

Code: CBHI valued program {5-0}~

P 1: _FGD_WOMEN_TREATMENT_TW.docx - 1:10 [verybody wants to become a mem..]  (101:101)   (Super)
Codes:	[CBHI valued program] [Poor reception of health workers:] [Service gap: Medicine unavailability ingovernment facility] 
No memos

verybody wants to become a member of CBHI regardless of whether they are poor or rich. Although there is often a shortage of medicines and problems with professional's reception, many people in our kebele need this CBHI.

P 1: _FGD_WOMEN_TREATMENT_TW.docx - 1:11 [Everyone is willing to pay and..]  (103:103)   (Super)
Codes:	[CBHI valued program] [Lack of money] 
No memos

Everyone is willing to pay and become a member. The thing that keeps them from paying voluntarily is the lack of money

P 1: _FGD_WOMEN_TREATMENT_TW.docx - 1:13 [CBHI is very useful. The only ..]  (106:106)   (Super)
Codes:	[CBHI valued program] [Service gap: Poor reception of health workers: it is painful] 
No memos

CBHI is very useful. The only place we have complaints is that health professionals do not kindly welcome people and do not treat people on time.

P 4: FGD_MEN_ treatment.docx - 4:2 [It is good that the government..]  (111:111)   (Super)
Codes:	[CBHI valued program] 
No memos

It is good that the government offers us this health insurance service.

P 9: KII_ __Cluster_CBHI Desk KII.docx - 9:32 [Mainly, now, CBHI is the most ..]  (62:62)   (Super)
Codes:	[CBHI valued program] 
No memos

Mainly, now, CBHI is the most important. Now, it is not only benefiting the community, but also benefiting the health institutions. So, before, health institutions were able to growth with their own, with their own income called health care financing, and the government made them go that way.

______________________________________________________________________

Code: CBHI_challenge: Exclusion of expensive drugs and investigationfrom the list of insurance {1-0}

P 3: FGD_Men__April 08,2024_.docx - 3:7 [I was informed that the mayor ..]  (90:90)   (Super)
Codes:	[CBHI_challenge: Exclusion of expensive drugs and investigationfrom the list of insurance] [CBHI_challenge: professionals refer us to their own clinic or laboratory] 
No memos

I was informed that the mayor had instructed them not to dispense expensive medicine through the insurance service. In general, in my opinion the implementation of health insurance has not correctly aligned with the government policy; I do not think that the government knows about excluding expensive medicines from insurance service. Another problem with the implementation of health insurance in addition to excluding expensive medicine is that they intentionally send you outside for X-Ray and Ultrasound imaging; those health workers who work on these services have their own clinic outside and they intentionally send you to outside to their clinic. Especially, those who are member of health insurance sent to private clinics intentionally. I tried to file a complaint to management of the hospital; after I tried so many times and rejected, I left it and took my wife to their clinic for X-Ray service. They [admin of hospital] are not ready to hear your complaint. I have seen when non-insurance members got X-Ray services while I dined only due to insurance service. 

______________________________________________________________________

Code: CBHI_challenge: not only professionals {1-0}

P 1: _FGD_WOMEN_TREATMENT_TW.docx - 1:7 [his means that anyone who has ..]  (97:97)   (Super)
Codes:	[CBHI _challenge: lack of understanding] [CBHI_challenge: not only professionals] [PB_CBHI: developed passion] [PB_CBHI: eager to renew] [Perceived benefits of CBHI: free service] 
No memos

his means that anyone who has seen and heard of its benefits is eager to renew their membership. For example, in the last year, a child of a relative of mine who is a member of CBHI was lying in bed for surgery at Tulu Bolo. The child was treated free of all service fees, including food. In the same ward, there was a man without CBHI being treated, and he was treated and discharged at great expense for similar services as that of a child of my relative. Since then, I have developed a passion for the CBHI. I also told people I knew about the benefits and made them interested. So in my opinion, it is a lack of understanding, but the problem of professionals alone does not prevent renewing the membership.

______________________________________________________________________

Code: CBHI_challenge: professionals refer us to their own clinic or laboratory {1-0}

P 3: FGD_Men__April 08,2024_.docx - 3:7 [I was informed that the mayor ..]  (90:90)   (Super)
Codes:	[CBHI_challenge: Exclusion of expensive drugs and investigationfrom the list of insurance] [CBHI_challenge: professionals refer us to their own clinic or laboratory] 
No memos

I was informed that the mayor had instructed them not to dispense expensive medicine through the insurance service. In general, in my opinion the implementation of health insurance has not correctly aligned with the government policy; I do not think that the government knows about excluding expensive medicines from insurance service. Another problem with the implementation of health insurance in addition to excluding expensive medicine is that they intentionally send you outside for X-Ray and Ultrasound imaging; those health workers who work on these services have their own clinic outside and they intentionally send you to outside to their clinic. Especially, those who are member of health insurance sent to private clinics intentionally. I tried to file a complaint to management of the hospital; after I tried so many times and rejected, I left it and took my wife to their clinic for X-Ray service. They [admin of hospital] are not ready to hear your complaint. I have seen when non-insurance members got X-Ray services while I dined only due to insurance service. 

______________________________________________________________________

Code: CBHI_governance: direction to give priority for members {0-0}
______________________________________________________________________

Code: CBHI_governance: leadership commitment {0-0}~
______________________________________________________________________

Code: CBHI_Governance: problem {0-0}
______________________________________________________________________

Code: CBHI_membership: lack money {1-0}

P 2: FGD_Men___TREATMENT.docx - 2:16 [the past, some people were not..]  (139:139)   (Super)
Codes:	[CBHI_membership: lack money] 
No memos

the past, some people were not enrolled in health insurance but at this time almost all are members. However, due to limited quotes for the poor to be enrolled,  very few people may not be enrolled because of lack of money.

______________________________________________________________________

Code: CBHI_money management: only for health facilities {0-0}
______________________________________________________________________

Code: CBHI_premium : difficult for some {0-0}
______________________________________________________________________

Code: CBHI_premium: not adequate  to cover the health care cost {0-0}
______________________________________________________________________

Code: CBHI_premium: not adequate  to cover the service {1-0}

P 9: KII_ __Cluster_CBHI Desk KII.docx - 9:1 [I still do not belief that con..]  (22:22)   (Super)
Codes:	[CBHI_premium: not adequate  to cover the service] 
No memos

 I still do not belief that contribution is enough because we all know the economic crisis in our country, not only in the country, but at the international level. The purchasing power of money is going down, the cost of living is getting very high, and because the resources that are spent on medical treatment are growing in a very serious manner, and they are also increasing in connection with the supply of medicines, perhaps the situation that is being covered is becoming very great fear

______________________________________________________________________

Code: CBHI_PREmium: not enough for health care cost {1-0}

P 9: KII_ __Cluster_CBHI Desk KII.docx - 9:8 [Yes, I mentioned it earlier. T..]  (34:34)   (Super)
Codes:	[CBHI_PREmium: not enough for health care cost] 
No memos

Yes, I mentioned it earlier. The money we collect from membership is not enough. As I said before, the economy is getting worse day by day. Therefore, associated with CBHI medical equipment, supply of medicine, the resources needed for health facilities, which has a high cost. As we know, even the medical facilities of our country, especially in relation to the production of these medicines, one was in Tigray. Now, because of the war, that has stopped. Because there is not much that is better elsewhere, so many things come from import. It is known how much it will cost in terms of dollars, so we cannot afford to cover this

______________________________________________________________________

Code: CBHI_premium: tansparen {1-0}

P 8: KII HEW_SCH__TREATMENT_TW.docx - 8:1 [There is transparency. A clien..]  (17:17)   (Super)
Codes:	[CBHI_premium: tansparen] 
No memos

There is transparency. A client's payment should not be lost. The receipt will be given to the members. The amount they pay is also recorded in their CBHI notebook. So there is no complaint in terms of transparency or fairness.

______________________________________________________________________

Code: CBHI_SS challenge: Additional payment for>18 years {1-0}

P 6: FGdD_Female_ treatment.docx - 6:4 [However, there are problems wi..]  (126:126)   (Super)
Codes:	[CBHI_SS challenge: Additional payment for>18 years] 
No memos

However, there are problems with categorizing lower, middle, and higher payers. If family members 18+ of age are informed to pay additional payments. My question is if a person lives with us what is the problem if he/she pays us with family? This causes problems for society. For example for the higher payer 1720 pay if that household has 18+ years, the government is forced to pay 200 and something for one individual. So it is better to service without additional payment as that individual has lived with as a family member.   

______________________________________________________________________

Code: CBHI_SS challenge: delay contribution {1-0}

P 1: _FGD_WOMEN_TREATMENT_TW.docx - 1:17 [the lack of drugs may be due t..]  (110:110)   (Super)
Codes:	[CBHI_SS challenge: delay contribution] [CBHI_SS challenge: Forced to pay] [CBHI_SS challenge: Increase fee] [CBHI_SS challenge: market inflation] [Service gap: Medicine unavailability ingovernment facility] 
No memos

the lack of drugs may be due to the current market situation. Another is that all the people do not pay equally on time. If all the people do not pay on time, both those who pay and those who do not pay may lose the medicine. Many people are not members because of the fees have increased so much this year. Last year it was 410 ETB. This year, the medium is 1260 ETB and the maximum is 1710 ETB. Many people, including myself, were opposed to this one, especially the maximum payment. Later, however, they came to our workplace and forced us to pay the CBHI payment with taxes. Generally, the community is more willing to pay except for higher prices.

______________________________________________________________________

Code: CBHI_SS challenge: Forced to pay {1-0}

P 1: _FGD_WOMEN_TREATMENT_TW.docx - 1:17 [the lack of drugs may be due t..]  (110:110)   (Super)
Codes:	[CBHI_SS challenge: delay contribution] [CBHI_SS challenge: Forced to pay] [CBHI_SS challenge: Increase fee] [CBHI_SS challenge: market inflation] [Service gap: Medicine unavailability ingovernment facility] 
No memos

the lack of drugs may be due to the current market situation. Another is that all the people do not pay equally on time. If all the people do not pay on time, both those who pay and those who do not pay may lose the medicine. Many people are not members because of the fees have increased so much this year. Last year it was 410 ETB. This year, the medium is 1260 ETB and the maximum is 1710 ETB. Many people, including myself, were opposed to this one, especially the maximum payment. Later, however, they came to our workplace and forced us to pay the CBHI payment with taxes. Generally, the community is more willing to pay except for higher prices.

______________________________________________________________________

Code: CBHI_SS challenge: high fee for middle group {3-0}

P 3: FGD_Men__April 08,2024_.docx - 3:3 [Insurance program is good to p..]  (89:89)   (Super)
Codes:	[CBHI_SS challenge: high fee for middle group] [Medicine unavailability in government facility] [PB_CBHI: protect from un necessary expense] 
No memos

Insurance program is good to protect our community from unnecessary expenses. However, this year the amount of payment has increased by more than double, which makes it difficult for many people especially poor households cannot pay for it despite they have interest to become member. Another major problem with health insurance, especially exacerbated this year is about unavailability of medicine from pharmacy of government health facilities. People are not getting medicines; they are being obligated to buy from private pharmacy at high cost. Sometimes, they may get from Red Cross pharmacy; however, government must work to improve the availability of medicine to sustain this health insurance

P 3: FGD_Men__April 08,2024_.docx - 3:14 [I am hypertensive and go to ho..]  (97:97)   (Super)
Codes:	[CBHI_SS challenge: high fee for middle group] [discrimination of insurance members: insulted] 
No memos

 I am hypertensive and go to hospital frequently; I have observed some problems about insurance service especially how health workers treat insurance member. One day a pharmacist insulted insurance members in front of me saying, “Those who are carrying insurance card immediately leave this area for those who can pay money for the medicine they are going to take”. I angrily asked that boy why he spoke like that; I told him that those insurance members also paid money for the government to get enough service from that hospital. Regarding the amount of payment, it is not affordable for some individuals, now people are facing challenges with the increased cost of living, I know some individuals who could not pay for insurance service and excluded from it. You can imagine how it is difficult to pay that much money while cost of everything being increased from time to time. In my opinion, government needs to re-evaluate about the amount of payment, it needs to be revised in a way that it could be affordable for many individuals.

P 3: FGD_Men__April 08,2024_.docx - 3:21 [Regarding screening process, I..]  (103:103)   (Super)
Codes:	[CBHI_SS challenge: high fee for middle group] [high category  member:  need only quality service not amount of payment] [Weakness and challenges SS-CBHI implementation(stratification) : no information provided] 
No memos

Regarding screening process, I have no information about who did it, and how they did it. We only informed our level and told how much to pay. I want to give some advice for you and the government on the amount of payment, especially concerning those who identified as medium level and asked to pay 1300 birr; many individuals indented as medium and supposed to pay 1300 birr cannot pay even 500 birr, majority of them have nothing as property. Rich individuals have no worry about the amount of payment, rather they complain about unavailability of medicines. In the future, government should find the mechanism to support those who identified as medium and nothing to pay. I know the government supports poor households [indigent HHs], which is very good; however, it is good if the quota of those supported by the government increased to include individuals identified in medium level. Regarding the increment of insurance payment from time to time, I have no complain as the price of medicine also being increased from time to time. If we pay the same amount each year while the price of medicine increases, how do you think the government able to provide those medicines for us?

______________________________________________________________________

Code: CBHI_SS challenge: high payment no medication {1-0}

P 3: FGD_Men__April 08,2024_.docx - 3:4 [This year, payment increased m..]  (89:89)   (Super)
Codes:	[CBHI_SS challenge: high payment no medication] [Trust on money collected: doubt if the money is collected for insurance purose] 
No memos

 This year, payment increased more than double and medicine availability decreased more than previous year, which creates some rumours in the community; they [members] do not believe that much payment was for insurance; rather they suspect that the government collected that money for other purpose in the name of health insurance.

______________________________________________________________________

Code: CBHI_SS challenge: Increase fee {1-0}~

P 1: _FGD_WOMEN_TREATMENT_TW.docx - 1:17 [the lack of drugs may be due t..]  (110:110)   (Super)
Codes:	[CBHI_SS challenge: delay contribution] [CBHI_SS challenge: Forced to pay] [CBHI_SS challenge: Increase fee] [CBHI_SS challenge: market inflation] [Service gap: Medicine unavailability ingovernment facility] 
No memos

the lack of drugs may be due to the current market situation. Another is that all the people do not pay equally on time. If all the people do not pay on time, both those who pay and those who do not pay may lose the medicine. Many people are not members because of the fees have increased so much this year. Last year it was 410 ETB. This year, the medium is 1260 ETB and the maximum is 1710 ETB. Many people, including myself, were opposed to this one, especially the maximum payment. Later, however, they came to our workplace and forced us to pay the CBHI payment with taxes. Generally, the community is more willing to pay except for higher prices.

______________________________________________________________________

Code: CBHI_SS challenge: market inflation {1-0}

P 1: _FGD_WOMEN_TREATMENT_TW.docx - 1:17 [the lack of drugs may be due t..]  (110:110)   (Super)
Codes:	[CBHI_SS challenge: delay contribution] [CBHI_SS challenge: Forced to pay] [CBHI_SS challenge: Increase fee] [CBHI_SS challenge: market inflation] [Service gap: Medicine unavailability ingovernment facility] 
No memos

the lack of drugs may be due to the current market situation. Another is that all the people do not pay equally on time. If all the people do not pay on time, both those who pay and those who do not pay may lose the medicine. Many people are not members because of the fees have increased so much this year. Last year it was 410 ETB. This year, the medium is 1260 ETB and the maximum is 1710 ETB. Many people, including myself, were opposed to this one, especially the maximum payment. Later, however, they came to our workplace and forced us to pay the CBHI payment with taxes. Generally, the community is more willing to pay except for higher prices.

______________________________________________________________________

Code: CBHI_SS challenge: the fee for insurance is low compared to the expected service {1-0}

P 3: FGD_Men__April 08,2024_.docx - 3:5 [As my friends said, insurance ..]  (90:90)   (Super)
Codes:	[CBHI_SS challenge: the fee for insurance is low compared to the expected service] [PB_CBHI: Free service for poor] 
No memos

As my friends said, insurance service benefitted us; especially poor individuals who cannot immediately go to health facility at the time of illness by their own payment. You can get so many services free of charge once you become member of health insurance; you will not pay for bed service, you will not pay for laboratory services, and medicines if available inside that health facility. In my opinion, the payment for health insurance membership is very low when compare it with the advantages we are getting free of charge.

______________________________________________________________________

Code: CBHI_SS challenge: Very low payment compared to expected service {1-0}

P 3: FGD_Men__April 08,2024_.docx - 3:22 [Regarding the amount of paymen..]  (104:104)   (Super)
Codes:	[CBHI_SS challenge: Very low payment compared to expected service] 
No memos

Regarding the amount of payment, it is too low for me; we all know how much we pay at private clinics, you can pay up to 3000 birr only for one laboratory investigation. The problem is when you pay for insurance membership and you gain buy medicine from private pharmacy. At the administrative level, our hospitals claim to offer a comprehensive range of services, except for neck and brain surgeries. However, contrary to these assurances, the reality on the ground is quite different, as even basic medications like paracetamol are often unavailable when you visit.

______________________________________________________________________

Code: CBHI_SS challenge: why fee Increased {1-0}

P 4: FGD_MEN_ treatment.docx - 4:14 [Some communities complain that..]  (131:131)   (Super)
Codes:	[CBHI_SS challenge: why fee Increased] [Premium contribution: fair compared to private facility service] 
No memos

 Some communities complain that the insurance membership fees have increased. This year, the community complains about why health insurance contribution has increased. However, when they compare with private health facilities, they voluntarily become health insurance members because the health insurance contribution is small.

______________________________________________________________________

Code: CBHI_SS_future suggestions:  stratification needs revision {1-0}

P 5: FGD_Women__treatment.docx - 5:18 [he resident should also be nee..]  (136:136)   (Super)
Codes:	[CBHI_SS_future suggestions:  stratification needs revision] 
No memos

he resident should also be need to be classified as there are huge economic gaps among the residents.  The quota given for indigent is very small. Poorest people are request to pay health insurance which is unaffordable. Thus, household categorization criteria need to be revised. There are people who utilized health insurance services which costs up to 80,000 birr and hence we have no doubt regarding its benefits. But consideration need to done for those who are unable pay the premium. 

______________________________________________________________________

Code: CBHI_SS_future suggestions: Awareness creation {1-0}

P 7: FGDs_Men___ treatment.docx - 7:26 [What needs to be done in impro..]  (160:160)   (Super)
Codes:	[CBHI_SS_future suggestions: Awareness creation] 
No memos

What needs to be done in improving this community-based health insurance, is improving community awareness on the amount of payment and how to use it. I saw that there was a huge limitation in the situation. That's what I know.

______________________________________________________________________

Code: CBHI_SS_future suggestions: Awareness creation on {1-0}

P 7: FGDs_Men___ treatment.docx - 7:16 [The service providers should a..]  (140:140)   (Super)
Codes:	[CBHI_SS_future suggestions: Awareness creation on] 
No memos

The service providers should also provide adequate awareness to our people

______________________________________________________________________

Code: CBHI_SS_future suggestions: Awareness creation on stratification {1-0}

P 4: FGD_MEN_ treatment.docx - 4:19 [To improve community's partici..]  (140:140)   (Super)
Codes:	[CBHI_SS_future suggestions: Awareness creation on stratification] 
No memos

To improve community's participation in CBHI program making health services available nearby. On another hand there are a few people who wonder why they pay more than each other and that can be improved by creating awareness.

______________________________________________________________________

Code: CBHI_SS_future suggestions: better to consider all resoource not only land for stratification {1-0}

P 1: _FGD_WOMEN_TREATMENT_TW.docx - 1:23 [I believe that a sliding scale..]  (118:118)   (Super)
Codes:	[CBHI_SS_future suggestions: better to consider all resoource not only land for stratification] [Sliding scale perceptions: positive] [Weakness and challenges SS-CBHI implementation : poor stratification] 
No memos

I believe that a sliding scale should be used for the contribution. However, classifying people according to their resources rather than their land area is incorrect because it makes small-scale landowners pay the same as large-scale landowners. For instance there was an equal premium contribution for a person who paid 15 ETB land tax and a person who paid 100 ETB land tax. In addition, there are cases where even the person who has no land is made a payer while the person who has land is classified as poor. In my opinion, since most of the resources are from the land, the contribution should be categorized based on the size of the land. 

______________________________________________________________________

Code: CBHI_SS_future suggestions: Improve laboratory service {1-0}

P 6: FGdD_Female_ treatment.docx - 6:45 [The second one is that the lab..]  (187:187)   (Super)
Codes:	[CBHI_SS_future suggestions: Improve laboratory service] 
No memos

The second one is that the laboratory service is not enough and standard. So we need standard laboratory service. The third we need doctors to avoid referrals.

______________________________________________________________________

Code: CBHI_SS_future suggestions: separating middle class in to two {1-0}

P 3: FGD_Men__April 08,2024_.docx - 3:29 [I think currently there are th..]  (116:116)   (Super)
Codes:	[CBHI_SS_future suggestions: separating middle class in to two] [strength of CBHI_SS implementation: stratification is income based] 
No memos

I think currently there are three level of payments; rich, medium and poor payers; in my opinion, it is good if the government increased this level to four or five levels, because there are economically different individuals in level two, medium level can again categorized to two or three depending on their economic status. If the government can do this, it is possible to accommodate those excluded due to inability to pay.

______________________________________________________________________

Code: Challenge- Photo of family members {0-0}
______________________________________________________________________

Code: challenge_SS_CBH: CBHI users malpractices {1-0}

P17: KII_CBHI Focal__ treatment.docx - 17:37 [There is also a problem with s..]  (49:49)   (Super)
Codes:	[challenge_SS_CBH: CBHI users malpractices] [challenge_SS_CBH: false demand] [challenge_SS_CBH: misuse of medicine] 
No memos

There is also a problem with some people in the community. The go to health facility without getting sick. Then, the receive medications. They again got to other health facility and receive medications. In this way they collect medicines from public health facilities and sell them to private pharmacy. For instance, clients receive ceftriaxone and sell it to private pharmacy. This is one of the challenge from the community side. As much as possible a health professional working on community pharmacy is registering clients who visit the pharmacy in order to identify their frequency of community pharmacy visit.

______________________________________________________________________

Code: challenge_SS_CBH: demand awareness {1-0}

P18: KII_CBHI focal__ treatment.docx - 18:24 [To be honest, CBHI members see..]  (31:31)   (Super)
Codes:	[challenge_SS_CBH: demand awareness] [challenge_SS_CBH: false demand] 
No memos

To be honest, CBHI members seek service than non-member individuals; sometimes they [members] visit health facilities while they are on travel for other purpose like when they come to the market or while they are on travel to visit their relatives. They keep their membership ID with them when they travel to market or other place to use it as opportunity to seek service.

______________________________________________________________________

Code: challenge_SS_CBH: false demand {4-0}

P17: KII_CBHI Focal__ treatment.docx - 17:36 [Health insurance increased the..]  (49:49)   (Super)
Codes:	[challenge_SS_CBH: false demand] [challenge_SS_CBH: unmet demand] 
No memos

Health insurance increased the health care seeking behavior of people. That is why health facilities are facing the shortage of medicines.

P17: KII_CBHI Focal__ treatment.docx - 17:37 [There is also a problem with s..]  (49:49)   (Super)
Codes:	[challenge_SS_CBH: CBHI users malpractices] [challenge_SS_CBH: false demand] [challenge_SS_CBH: misuse of medicine] 
No memos

There is also a problem with some people in the community. The go to health facility without getting sick. Then, the receive medications. They again got to other health facility and receive medications. In this way they collect medicines from public health facilities and sell them to private pharmacy. For instance, clients receive ceftriaxone and sell it to private pharmacy. This is one of the challenge from the community side. As much as possible a health professional working on community pharmacy is registering clients who visit the pharmacy in order to identify their frequency of community pharmacy visit.

P18: KII_CBHI focal__ treatment.docx - 18:24 [To be honest, CBHI members see..]  (31:31)   (Super)
Codes:	[challenge_SS_CBH: demand awareness] [challenge_SS_CBH: false demand] 
No memos

To be honest, CBHI members seek service than non-member individuals; sometimes they [members] visit health facilities while they are on travel for other purpose like when they come to the market or while they are on travel to visit their relatives. They keep their membership ID with them when they travel to market or other place to use it as opportunity to seek service.

P18: KII_CBHI focal__ treatment.docx - 18:26 [Some patient may return to hea..]  (31:31)   (Super)
Codes:	[challenge_SS_CBH: false demand] 
No memos

Some patient may return to health facility without completing the medicine given for them and again take another medicine. Therefore, health workers are expected to ask their previous history and counsel them to complete their treatment first before asking for another medicine or service. 

______________________________________________________________________

Code: challenge_SS_CBH: government budget {1-0}

P15: KII PHCU Leader___TREATMENT TW.docx - 15:6 [The budget allocated and the s..]  (22:22)   (Super)
Codes:	[challenge_SS_CBH: government budget] 
No memos

The budget allocated and the services we provide are not exactly equal. Because the budget allocated for the health center is only 300,000 ETB and the population using the health center is over 44000 people, Additionally, the prices of medicines have increased dramatically. As a result, we sometimes took money from health care finances.

______________________________________________________________________

Code: challenge_SS_CBH: high demand {1-0}

P19: KII_Head of  health Office_ treatment.docx - 19:30 [. Health seeking behavior of t..]  (59:59)   (Super)
Codes:	[challenge_SS_CBH: high demand] [Impact_SS_CBHI: health seeking behavior] 
No memos

. Health seeking behavior of the community increased after the initiation of health insurance. The community has no worries about medical costs for a year once they paid the premium. But there is a shortage of drugs. To my best understanding as experienced health professional, the shortage of drugs is due to the increment of health seeking/demand, not due to health insurance.

______________________________________________________________________

Code: challenge_SS_CBH: informal discussions {1-0}

P14: KII HEW__2024_.docx - 14:21 [Seeking care practice of our c..]  (38:38)   (Super)
Codes:	[challenge_SS_CBH: informal discussions] 
No memos

Seeking care practice of our community is good. For example, they had a discussion there. They tell us that there is a problem supplies. That's how you know they are seeking for care. They want to be treated properly. They even show us the medicine they take.

______________________________________________________________________

Code: challenge_SS_CBH: low premium {1-0}

P15: KII PHCU Leader___TREATMENT TW.docx - 15:8 [But the budget is not enough t..]  (22:22)   (Super)
Codes:	[challenge_SS_CBH: low premium] 
No memos

But the budget is not enough to provide adequate services to our community, and it is often difficult for us. To solve this, we are preparing to open a community pharmacy.

______________________________________________________________________

Code: challenge_SS_CBH: misuse of medicine {1-0}

P17: KII_CBHI Focal__ treatment.docx - 17:37 [There is also a problem with s..]  (49:49)   (Super)
Codes:	[challenge_SS_CBH: CBHI users malpractices] [challenge_SS_CBH: false demand] [challenge_SS_CBH: misuse of medicine] 
No memos

There is also a problem with some people in the community. The go to health facility without getting sick. Then, the receive medications. They again got to other health facility and receive medications. In this way they collect medicines from public health facilities and sell them to private pharmacy. For instance, clients receive ceftriaxone and sell it to private pharmacy. This is one of the challenge from the community side. As much as possible a health professional working on community pharmacy is registering clients who visit the pharmacy in order to identify their frequency of community pharmacy visit.

______________________________________________________________________

Code: challenge_SS_CBH: priority for payers {1-0}

P17: KII_CBHI Focal__ treatment.docx - 17:35 [The other reason for non-membe..]  (45:45)   (Super)
Codes:	[challenge_SS_CBH: priority for payers] [Reasons for not becoming member_SS_CBHI: ignorance of HWs CBHI users] 
No memos

The other reason for non-membership was due to lack of compassionate health workers. In _ , we made two windows at card room: one for insurance member and the other for MCH/exempted services. This is to serve all clients equally as we had been hearing complain from the clients about the existence of delayed services for MCH/exempted services.  

______________________________________________________________________

Code: challenge_SS_CBH: unmet demand {1-0}

P17: KII_CBHI Focal__ treatment.docx - 17:36 [Health insurance increased the..]  (49:49)   (Super)
Codes:	[challenge_SS_CBH: false demand] [challenge_SS_CBH: unmet demand] 
No memos

Health insurance increased the health care seeking behavior of people. That is why health facilities are facing the shortage of medicines.

______________________________________________________________________

Code: Challenge_SS_CBHI: awareness of referral system {0-0}
______________________________________________________________________

Code: Challenge_SS_CBHI: awareness of the scheme {0-0}
______________________________________________________________________

Code: Challenge_SS_CBHI: false demand {2-0}

P13: KII _ _HC-Head _ Ttreatment.docx - 13:14 [On the other hand, they have e..]  (33:33)   (Super)
Codes:	[Challenge_SS_CBHI: false demand] 
No memos

On the other hand, they have enough understanding to go to the health facility not only for treatment but also for checkups. They have insurance so if they get a little sick they come and seek services.

P14: KII HEW__2024_.docx - 14:20 [Insurance has a huge impact on..]  (36:36)   (Super)
Codes:	[Challenge_SS_CBHI: false demand] [Impact_SS_CBHI: health seeking behavior] 
No memos

Insurance has a huge impact on health service provision. If we don't use it now, we can't save it for next year. The people are mature now. The people go there and take it from here to the governor. So unless there is enough service there, it will be a big obstacle for us again. 

______________________________________________________________________

Code: Challenge_SS_CBHI: high claim {1-0}

P11: KII _Town HO head_ Treatment.docx - 11:20 [The question of reimbursing is..]  (42:42)   (Super)
Codes:	[Challenge_SS_CBHI: high claim] [Challenge_SS_CBHI: reimbursement] 
No memos

The question of reimbursing is impossible because of the small amount of money and the difficulty of persisting in the program. The money we collected from the household was not enough not only for a year but also not enough payment at once.  It's not simply to cover the bed, drugs, laboratory, and food during their stay in the health facility which may take from 1-3 months admission

______________________________________________________________________

Code: Challenge_SS_CBHI: High expectation {1-0}

P11: KII _Town HO head_ Treatment.docx - 11:18 [Even though we try our best in..]  (40:40)   (Super)
Codes:	[Challenge_SS_CBHI: High expectation] 
No memos

Even though we try our best in different aspects, we don't believe 100% of drugs are available. So if someone comes to a health facility can get 87%+ drugs from the health facilities. So if the remaining drugs are from outside no matter what he gets. 

______________________________________________________________________

Code: Challenge_SS_CBHI: lack of coordination {1-0}

P12: KII CBHI focal___TREATMENT TW.docx - 12:7 [Another obstacle is that the b..]  (21:21)   (Super)
Codes:	[Challenge_SS_CBHI: lack of coordination] 
No memos

Another obstacle is that the bodies supporting CBHI are not delivering the necessary resources to the community as required. For example, the lack of coordination between kebele structures and other government sectors on CBHI activities is an obstacle.

______________________________________________________________________

Code: Challenge_SS_CBHI: large catchment for hospitals {0-0}
______________________________________________________________________

Code: Challenge_SS_CBHI: overlap of activities {1-0}

P11: KII _Town HO head_ Treatment.docx - 11:24 [But is going on that the healt..]  (51:51)   (Super)
Codes:	[Challenge_SS_CBHI: overlap of activities] 
No memos

But is going on that the health sectors providing service, second procurement, and regulatory bodies. If such things are not corrected, the health sector exposed to inefficiency because there is no external body to evaluate it. The complaints of the community may not get a solution because of the health sector service provider and complain receiver.

______________________________________________________________________

Code: Challenge_SS_CBHI: Patient load at hospitals {0-0}
______________________________________________________________________

Code: Challenge_SS_CBHI: premium collection process {0-0}
______________________________________________________________________

Code: Challenge_SS_CBHI: reimbursement {1-0}

P11: KII _Town HO head_ Treatment.docx - 11:20 [The question of reimbursing is..]  (42:42)   (Super)
Codes:	[Challenge_SS_CBHI: high claim] [Challenge_SS_CBHI: reimbursement] 
No memos

The question of reimbursing is impossible because of the small amount of money and the difficulty of persisting in the program. The money we collected from the household was not enough not only for a year but also not enough payment at once.  It's not simply to cover the bed, drugs, laboratory, and food during their stay in the health facility which may take from 1-3 months admission

______________________________________________________________________

Code: Challenge_SS_CBHI: resources {1-0}

P12: KII CBHI focal___TREATMENT TW.docx - 12:10 [When we talk about the quality..]  (27:27)   (Super)
Codes:	[Challenge_SS_CBHI: resources] [Reasons for not becoming member_SS_CBHI: human and financial resource] [Reasons for not becoming member_SS_CBHI: poor quality] 
No memos

When we talk about the quality of health care, we mean giving someone who has come in need what they want in a complete way. This may not happen due to problems such as a lack of medical equipment and human resources. Therefore, there are still deficiencies in health quality that need to be improved. This decline in health quality, along with other problems, can cause problems in the recruitment and retention of CBHI members.

______________________________________________________________________

Code: Challenge_SS_CBHI: seeking care on non-workng days {0-0}
______________________________________________________________________

Code: Challenge_SS_CBHI: structure and fund to tor CBHI activities {1-0}

P12: KII CBHI focal___TREATMENT TW.docx - 12:8 [Lack of funds is also a constr..]  (21:21)   (Super)
Codes:	[Challenge_SS_CBHI: structure and fund to tor CBHI activities] 
No memos

Lack of funds is also a constraint on this work. In particular, lack of funds plays a major role in the lack of provision and monitoring of CBHI activities. I

______________________________________________________________________

Code: Challenge_SS_CBHI:khat market {1-0}

P14: KII HEW__2024_.docx - 14:19 [The community has no complaint..]  (32:32)   (Super)
Codes:	[Challenge_SS_CBHI:khat market] 
No memos

The community has no complaints about the paying ability except for a bit at the moment because chat prices have dropped. They have no lack of understanding.   A little bit on the financial and economic crisis right now.

______________________________________________________________________

Code: challengs {0-0}
______________________________________________________________________

Code: Challengs of the pilot_SS_CBHI:  awareness of the pilot {2-0}

P14: KII HEW__2024_.docx - 14:22 [Those who paid high and those ..]  (38:38)   (Super)
Codes:	[Challengs of the pilot_SS_CBHI:  awareness of the pilot] [Challengs of the pilot_SS_CBHI:  premium vs service slide] 
No memos

Those who paid high and those who paid low complain that they are being treated the same and what is the difference.

P20: KII_Kebele manager__treatment.docx - 20:14 [however, there has been some r..]  (19:19)   (Super)
Codes:	[Challengs of the pilot_SS_CBHI:  awareness of the pilot] [Challengs of the pilot_SS_CBHI: differences among s] 
No memos

however, there has been some rumours and confusion among the community regarding the different payment system in our  and neighbouring s; some farmers, especially those residing neighbour to adjacent s asked why the payment in our  is different while farmers in other s pay the same amount [flat rate]. 

______________________________________________________________________

Code: Challengs of the pilot_SS_CBHI:  basis for sliding {1-0}

P20: KII_Kebele manager__treatment.docx - 20:31 [During the screening the major..]  (35:35)   (Super)
Codes:	[Challengs of the pilot_SS_CBHI:  basis for sliding] [Challengs of the pilot_SS_CBHI:  economic status vs land ownership] 
No memos

During the screening the major problem we faced was land ownership versus economic status of the households; some farmers have large farmland and economically poor, and some farmers do not own land and economically rich. Therefore, if we fail to measure other assists during screening, we may misplace the households. Households who do not have farmland and economically rich do not want to be identified as high payer households. Before going for screening, we need to inform the households regarding what assets are going to be considered while the screening process to minimize complaints and errors.

______________________________________________________________________

Code: Challengs of the pilot_SS_CBHI:  economic status vs family size {2-0}

P13: KII _ _HC-Head _ Ttreatment.docx - 13:18 [The challenge may come from a ..]  (39:39)   (Super)
Codes:	[Challengs of the pilot_SS_CBHI:  economic status vs family size] 
No memos

The challenge may come from a lack of understanding. For example, among the people, why should I pay more when I have fewer family members? There may be those who say that the so-called poor has many more family members than me but pays less.

P16: KII__HEW_ _ _.docx - 16:18 [The challenge we may face is t..]  (39:39)   (Super)
Codes:	[Challengs of the pilot_SS_CBHI:  economic status vs family size] 
No memos

The challenge we may face is that some high payer households say why we pay so much and we don't have families as equal as the low payer ones. We have fewer family members when compared to low payer ones.

______________________________________________________________________

Code: Challengs of the pilot_SS_CBHI:  economic status vs land ownership {1-0}

P20: KII_Kebele manager__treatment.docx - 20:31 [During the screening the major..]  (35:35)   (Super)
Codes:	[Challengs of the pilot_SS_CBHI:  basis for sliding] [Challengs of the pilot_SS_CBHI:  economic status vs land ownership] 
No memos

During the screening the major problem we faced was land ownership versus economic status of the households; some farmers have large farmland and economically poor, and some farmers do not own land and economically rich. Therefore, if we fail to measure other assists during screening, we may misplace the households. Households who do not have farmland and economically rich do not want to be identified as high payer households. Before going for screening, we need to inform the households regarding what assets are going to be considered while the screening process to minimize complaints and errors.

______________________________________________________________________

Code: Challengs of the pilot_SS_CBHI:  flat rate s {1-0}

P18: KII_CBHI focal__ treatment.docx - 18:14 [We faced some challenges from ..]  (19:19)   (Super)
Codes:	[Challengs of the pilot_SS_CBHI:  flat rate s] 
No memos

We faced some challenges from households those share border with adjacent s; they questioned us why their payment system was different while the neighbouring  pay equally [960 ETB]. We solved this challenge telling them that the system is being tested in some s and gradually expand to all s. Additionally, we told the community that the government is about to increase the amount of payment to improve availability of medicines and laboratory services as the price of these items increases from time to time. 

______________________________________________________________________

Code: Challengs of the pilot_SS_CBHI:  premium vs service slide {2-0}

P14: KII HEW__2024_.docx - 14:22 [Those who paid high and those ..]  (38:38)   (Super)
Codes:	[Challengs of the pilot_SS_CBHI:  awareness of the pilot] [Challengs of the pilot_SS_CBHI:  premium vs service slide] 
No memos

Those who paid high and those who paid low complain that they are being treated the same and what is the difference.

P14: KII HEW__2024_.docx - 14:25 [If the community pays based on..]  (44:44)   (Super)
Codes:	[Challengs of the pilot_SS_CBHI:  premium vs service slide] 
No memos

If the community pays based on the economic situation, there will be no obstacles. Yes, you know the community from their point of view in treatment. Some farmers say that just as contribute based on economic status, you will serve us differently in treatment. 

______________________________________________________________________

Code: Challengs of the pilot_SS_CBHI:  risk of fa_re if service quality not improved {1-0}

P20: KII_Kebele manager__treatment.docx - 20:28 [The community may resist it if..]  (35:35)   (Super)
Codes:	[Challengs of the pilot_SS_CBHI:  risk of fa_re if service quality not improved] 
No memos

The community may resist it if the government fail to provide adequate services especially adequate medicines.

______________________________________________________________________

Code: Challengs of the pilot_SS_CBHI:  stratification fairness {5-0}

P11: KII _Town HO head_ Treatment.docx - 11:10 [In some cases, deliberately co..]  (26:26)   (Super)
Codes:	[Challengs of the pilot_SS_CBHI:  stratification fairness] 
No memos

In some cases, deliberately collecting money from high-income persons the quota of middle income.    This program should be sustainable. If it goes bankrupt, we are going to fail in this program.

P12: KII CBHI focal___TREATMENT TW.docx - 12:21 [As is well known, we had estab..]  (43:43)   (Super)
Codes:	[Challengs of the pilot_SS_CBHI:  stratification fairness] 
No memos

As is well known, we had established a community resource committee to divide the community into three parts. However, these committees had serious shortcomings in properly identifying the resources of the community and classifying them as poor, middle and high. Therefore, the fact that the committee did not meet and divide the community fairly was a big obstacle for us.

P15: KII PHCU Leader___TREATMENT TW.docx - 15:5 [There have been problems in th..]  (20:20)   (Super)
Codes:	[Challengs of the pilot_SS_CBHI:  stratification fairness] 
No memos

There have been problems in the process of sliding-scale implementation. For example, they had brought in someone I knew to be poor as a payer and someone who had wealth as a poor man. I have taken this one out of the payer who is truly poor by my own decision, categorized him as poor, and returned the one who is said to be poor despite having wealth to the payer.

P17: KII_CBHI Focal__ treatment.docx - 17:6 [The Ganda level committee were..]  (23:23)   (Super)
Codes:	[Challengs of the pilot_SS_CBHI:  stratification fairness] [Challengs of the pilot_SS_CBHI:  transparency and fairness] 
No memos

The Ganda level committee were not properly categorizing the households based on the criteria. They were making bias, they were categorizing their socials to lower level category even though they have high economy. They were not relying on the criteria. For instance, in Washa kebele, one of the committee member was mobilizing the community to pay only 720 birr. 

P17: KII_CBHI Focal__ treatment.docx - 17:42 [But the problem was lack of fa..]  (54:54)   (Super)
Codes:	[Challengs of the pilot_SS_CBHI:  stratification fairness] 
No memos

But the problem was lack of fairness and transparency during household categorization to the three level. This complain was repeatedly heard from the community. We will work hard to correct this issue in the next year. 

______________________________________________________________________

Code: Challengs of the pilot_SS_CBHI:  stratification quality {1-0}

P18: KII_CBHI focal__ treatment.docx - 18:32 [However, there have been some ..]  (37:37)   (Super)
Codes:	[Challengs of the pilot_SS_CBHI:  stratification quality] 
No memos

However, there have been some complaints regarding the classification of households to different categories. There has been fairness question on this classification

______________________________________________________________________

Code: Challengs of the pilot_SS_CBHI:  transparency and fairness {2-0}

P17: KII_CBHI Focal__ treatment.docx - 17:4 [They complain that the committ..]  (19:19)   (Super)
Codes:	[Challengs of the pilot_SS_CBHI:  transparency and fairness] 
No memos

They complain that the committees were not fairly and transparently categorizing the households based on their economy. People were saying the committee is categorizing households of the same economy into different CBHI categories. Committee were making favor for their socials/friends and categorizing households of high economy to lower category.

P17: KII_CBHI Focal__ treatment.docx - 17:6 [The Ganda level committee were..]  (23:23)   (Super)
Codes:	[Challengs of the pilot_SS_CBHI:  stratification fairness] [Challengs of the pilot_SS_CBHI:  transparency and fairness] 
No memos

The Ganda level committee were not properly categorizing the households based on the criteria. They were making bias, they were categorizing their socials to lower level category even though they have high economy. They were not relying on the criteria. For instance, in Washa kebele, one of the committee member was mobilizing the community to pay only 720 birr. 

______________________________________________________________________

Code: Challengs of the pilot_SS_CBHI: absence of kebele structure {1-0}

P18: KII_CBHI focal__ treatment.docx - 18:7 [The screening was performed by..]  (11:11)   (Super)
Codes:	[Challengs of the pilot_SS_CBHI: absence of kebele structure] [Challengs of the pilot_SS_CBHI: workload] 
No memos

The screening was performed by our health workers due to inexistence of kebele structure in our woreda. This screening was performed by moving home to home throughout all kebeles in our , which was a huge burden for our staff and was taken a long time to complete. 

______________________________________________________________________

Code: Challengs of the pilot_SS_CBHI: additional paymen for additional family member {1-0}

P17: KII_CBHI Focal__ treatment.docx - 17:16 [Previously, if there are peopl..]  (29:29)   (Super)
Codes:	[Challengs of the pilot_SS_CBHI: additional paymen for additional family member] 
No memos

Previously, if there are people of age greater than 18 years in the household, they contribute additional 20 percent of the standard of each category. But the problem was that committee was making some mistakes. They forget to add the additional payment of 50% for the second wife and 20% for each individual greater than 18 years in the family member. When such people came to our office for renewal, we were returning them to pay the additional percentage and come back for renewal. This causes the customers to wonder there and there, frequently visit our office.

______________________________________________________________________

Code: Challengs of the pilot_SS_CBHI: ambiguity {1-0}

P17: KII_CBHI Focal__ treatment.docx - 17:7 [Because if the households pay ..]  (23:23)   (Super)
Codes:	[Challengs of the pilot_SS_CBHI: ambiguity] [Challengs of the pilot_SS_CBHI: confusion on premium] 
No memos

Because if the households pay based on the three level categories, we was perceiving that economy based CBHI payment will continue in a similar ways from this year onwards. Most of the households in Washa kebele paid 720 birr. Later on, after many debt and conflicts, we categorized the households based on their economy. Thus, the payment process lacks fairness and transparency in some kebeles of our . 

______________________________________________________________________

Code: Challengs of the pilot_SS_CBHI: awareness {3-0}

P15: KII PHCU Leader___TREATMENT TW.docx - 15:3 [As is well known, it is diffic..]  (16:16)   (Super)
Codes:	[Challengs of the pilot_SS_CBHI: awareness] [Challengs of the pilot_SS_CBHI: raised premium] 
No memos

As is well known, it is difficult to change the ideas and attitudes of society at once. They were refusing because they did not have an understanding of the cost of health services such as medicines. Later, they became aware of it and became members without any objection. However, there is still a lack of understanding among parents who take payment seriously and fear it.

P17: KII_CBHI Focal__ treatment.docx - 17:10 [This was the main gap in the C..]  (27:27)   (Super)
Codes:	[Challengs of the pilot_SS_CBHI: awareness] [Challengs of the pilot_SS_CBHI: urgency] 
No memos

This was the main gap in the CBHI premium collection of this year. It was a new approach for our  to categorize the households based on their economy. But as the time/schedule was too tight, the communities were not well informed about the categories and criteria of categorization. 

P17: KII_CBHI Focal__ treatment.docx - 17:13 [The other challenge was that h..]  (27:27)   (Super)
Codes:	[Challengs of the pilot_SS_CBHI: awareness] [Challengs of the pilot_SS_CBHI: premium collection methods] [Challengs of the pilot_SS_CBHI: transparency of premium] 
No memos

The other challenge was that households receive a number voucher/receipt at a time (hawaasni al-tokkotti nagahee hedduu akka kutan gaafatamuu). CBHI is linked to a number of voucher. They were saying that we have interest to pay for CBHI even more amount of money (e.g.3000 birr). But due to linkage to receipts, we refuse/agitate to pay for CBHI too. Most of the time, households don't know the amount of money they paid for CBHI as it is summed up with other vouchers. This also give opportunity for cheating as the household may not know for which services they paid. Indeed, sometimes those who collect the premium desire to visit home to home than collecting the money at office to facilitate for cheating.

______________________________________________________________________

Code: Challengs of the pilot_SS_CBHI: awareness of the pilot {2-0}

P17: KII_CBHI Focal__ treatment.docx - 17:1 [Regarding the premium collecti..]  (19:19)   (Super)
Codes:	[Challengs of the pilot_SS_CBHI: awareness of the pilot] [Challengs of the pilot_SS_CBHI: differences among s] 
No memos

Regarding the premium collection process, the community were saying all households in our neighbor s are paying equal amount, why we pay based on categories.

P17: KII_CBHI Focal__ treatment.docx - 17:12 [The premium collection was mad..]  (27:27)   (Super)
Codes:	[Challengs of the pilot_SS_CBHI: awareness of the pilot] [Challengs of the pilot_SS_CBHI: differences among s] 
No memos

The premium collection was made by visiting home to home. Some of the households were also asking question such as why the category was made as our relatives in other s are paying similar amount of money. Even though they asked, they hadn't get enough answer

______________________________________________________________________

Code: Challengs of the pilot_SS_CBHI: confusion on premium {1-0}

P17: KII_CBHI Focal__ treatment.docx - 17:7 [Because if the households pay ..]  (23:23)   (Super)
Codes:	[Challengs of the pilot_SS_CBHI: ambiguity] [Challengs of the pilot_SS_CBHI: confusion on premium] 
No memos

Because if the households pay based on the three level categories, we was perceiving that economy based CBHI payment will continue in a similar ways from this year onwards. Most of the households in Washa kebele paid 720 birr. Later on, after many debt and conflicts, we categorized the households based on their economy. Thus, the payment process lacks fairness and transparency in some kebeles of our . 

______________________________________________________________________

Code: Challengs of the pilot_SS_CBHI: coordination {1-0}

P17: KII_CBHI Focal__ treatment.docx - 17:11 [During our meeting with projec..]  (27:27)   (Super)
Codes:	[Challengs of the pilot_SS_CBHI: coordination] [Challengs of the pilot_SS_CBHI: urgency] 
No memos

During our meeting with project central team, we were informed that we will get plenty time for creating awareness of the community. However, later on we informed to complete the premium collection processes with similar schedule to other s that use flat scale approach. Thus, due to a shortage of time schedule, people were not well informed about the issue. This creates resistance even among some of the Ganda level committee members.

______________________________________________________________________

Code: Challengs of the pilot_SS_CBHI: deviating from enrollment protocol _ {1-0}

P19: KII_Head health Office_ treatment.docx - 19:4 [Yes, both girls and boys need ..]  (25:25)   (Super)
Codes:	[Challengs of the pilot_SS_CBHI: deviating from enrollment protocol _] 
No memos

Yes, both girls and boys need to be independent to be CBHI member. In case, they are unable to pay (e.g. students), they can be considered as indigent and the government cover the CBHI payment for them or they get donation from others such as government employees. 

______________________________________________________________________

Code: Challengs of the pilot_SS_CBHI: differences among s {5-0}

P14: KII HEW__2024_.docx - 14:29 [The differences between the di..]  (52:52)   (Super)
Codes:	[Challengs of the pilot_SS_CBHI: differences among s] 
No memos

The differences between the s, people there will hear each other and why does another  pay like this and why do we pay like this? This has been a bit of a big obstacle for us. 

P17: KII_CBHI Focal__ treatment.docx - 17:1 [Regarding the premium collecti..]  (19:19)   (Super)
Codes:	[Challengs of the pilot_SS_CBHI: awareness of the pilot] [Challengs of the pilot_SS_CBHI: differences among s] 
No memos

Regarding the premium collection process, the community were saying all households in our neighbor s are paying equal amount, why we pay based on categories.

P17: KII_CBHI Focal__ treatment.docx - 17:12 [The premium collection was mad..]  (27:27)   (Super)
Codes:	[Challengs of the pilot_SS_CBHI: awareness of the pilot] [Challengs of the pilot_SS_CBHI: differences among s] 
No memos

The premium collection was made by visiting home to home. Some of the households were also asking question such as why the category was made as our relatives in other s are paying similar amount of money. Even though they asked, they hadn't get enough answer

P19: KII_Head of  health Office_ treatment.docx - 19:21 [For example, our community was..]  (47:47)   (Super)
Codes:	[Challengs of the pilot_SS_CBHI: differences among s] [Challengs of the pilot_SS_CBHI: suspecting service improvement] 
No memos

For example, our community was saying, all people in our neighbor  are paying equal amount of money for CBHI membership, why it is different in our . We were also creating awareness to the community that with the previous approach (flat scale), we can't achieve the goal of health insurance which is a culture of social support. The community were also not refusing to pay, rather they were requesting for the availability of services at health facilities. 

P20: KII_Kebele manager__treatment.docx - 20:14 [however, there has been some r..]  (19:19)   (Super)
Codes:	[Challengs of the pilot_SS_CBHI:  awareness of the pilot] [Challengs of the pilot_SS_CBHI: differences among s] 
No memos

however, there has been some rumours and confusion among the community regarding the different payment system in our  and neighbouring s; some farmers, especially those residing neighbour to adjacent s asked why the payment in our  is different while farmers in other s pay the same amount [flat rate]. 

______________________________________________________________________

Code: Challengs of the pilot_SS_CBHI: excutors fairness {1-0}

P17: KII_CBHI Focal__ treatment.docx - 17:9 [However, the ganda level commi..]  (25:25)   (Super)
Codes:	[Challengs of the pilot_SS_CBHI: excutors fairness] 
No memos

However, the ganda level committee made unfairness and lacks transparency in some area. 

______________________________________________________________________

Code: Challengs of the pilot_SS_CBHI: fulfilling expectations after the pilot {1-0}

P20: KII_Kebele manager__treatment.docx - 20:27 [the major challenge, I repeate..]  (35:35)   (Super)
Codes:	[Challengs of the pilot_SS_CBHI: fulfilling expectations after the pilot] 
No memos

 the major challenge, I repeatedly raised in this discussion is about poor medicine availability in health facilities; if our health facilities continue with the same practice [unavailability of medicine], increasing the amount of payment and different payment amount for different households will not sustain. 

______________________________________________________________________

Code: Challengs of the pilot_SS_CBHI: government budget {1-0}

P15: KII PHCU Leader___TREATMENT TW.docx - 15:14 [In my opinion, the main obstac..]  (34:34)   (Super)
Codes:	[Challengs of the pilot_SS_CBHI: government budget] [Challengs of the pilot_SS_CBHI:medicine unavailability] 
No memos

In my opinion, the main obstacle in the beginning is the lack of medicines. Next, the budget is allocated equally to different levels of health centers, which causes difficulties in providing services. This means that the government allocates 300,000 ETB for grade 'A' health centers, equal to grade 'B' and 'C' health centers. This has a serious problem because a grade 'A' health center serves a large population and there is a shortage of medicines. In contrast, it causes the waste of medicines in grade 'B' and 'C' health centers.

______________________________________________________________________

Code: Challengs of the pilot_SS_CBHI: involvement of health workers {1-0}

P18: KII_CBHI focal__ treatment.docx - 18:8 [Only three or four kebeles had..]  (11:11)   (Super)
Codes:	[Challengs of the pilot_SS_CBHI: involvement of health workers] [Challengs of the pilot_SS_CBHI: time consuming] 
No memos

Only three or four kebeles had structure and supported as in screening process. In those kebeles, kebele leaders and kebele managers participated in screening process. Due to the lack of a kebele structure, health workers handled the majority of all procedures, from screening to money collection. This process [screening and collecting payments] is a major activity in implementing sliding scale, which needs time, and enough human resource. Even we recorded it is a major challenge we faced in implementation process in our woreda.

______________________________________________________________________

Code: Challengs of the pilot_SS_CBHI: mis-stratification {1-0}

P18: KII_CBHI focal__ treatment.docx - 18:3 [The main reason is that the ma..]  (9:9)   (Super)
Codes:	[Challengs of the pilot_SS_CBHI: mis-stratification] [Challengs of the pilot_SS_CBHI: rsistance from high level HHs] 
No memos

The main reason is that the majority of the households identified as higher did not believe and accept their level and denied to pay the required amount. During stratification, we used so many criteria like land, cattle, and other sources of income. However, still there are so many complains especially from identified high-level households. To be honest, there might be misclassification 

______________________________________________________________________

Code: Challengs of the pilot_SS_CBHI: political interferance {1-0}

P17: KII_CBHI Focal__ treatment.docx - 17:15 [The other challenge was big pr..]  (27:27)   (Super)
Codes:	[Challengs of the pilot_SS_CBHI: political interferance] [Suggestions to improve the pilot_SS_CBHI: autonomy] 
No memos

The other challenge was big pressure from those on higher positions. There is a pressure on payers to pay the CBHI premium. But once shouldn't be forced to pay to become CBHI member. People should understand, accept and should have willingness for CBHI to receive health services. Thus, CBHI is highly politicalized and people forced to pay. This need to be improved in the future. 

______________________________________________________________________

Code: Challengs of the pilot_SS_CBHI: premium collection methods {1-0}

P17: KII_CBHI Focal__ treatment.docx - 17:13 [The other challenge was that h..]  (27:27)   (Super)
Codes:	[Challengs of the pilot_SS_CBHI: awareness] [Challengs of the pilot_SS_CBHI: premium collection methods] [Challengs of the pilot_SS_CBHI: transparency of premium] 
No memos

The other challenge was that households receive a number voucher/receipt at a time (hawaasni al-tokkotti nagahee hedduu akka kutan gaafatamuu). CBHI is linked to a number of voucher. They were saying that we have interest to pay for CBHI even more amount of money (e.g.3000 birr). But due to linkage to receipts, we refuse/agitate to pay for CBHI too. Most of the time, households don't know the amount of money they paid for CBHI as it is summed up with other vouchers. This also give opportunity for cheating as the household may not know for which services they paid. Indeed, sometimes those who collect the premium desire to visit home to home than collecting the money at office to facilitate for cheating.

______________________________________________________________________

Code: Challengs of the pilot_SS_CBHI: raised premium {1-0}

P15: KII PHCU Leader___TREATMENT TW.docx - 15:3 [As is well known, it is diffic..]  (16:16)   (Super)
Codes:	[Challengs of the pilot_SS_CBHI: awareness] [Challengs of the pilot_SS_CBHI: raised premium] 
No memos

As is well known, it is difficult to change the ideas and attitudes of society at once. They were refusing because they did not have an understanding of the cost of health services such as medicines. Later, they became aware of it and became members without any objection. However, there is still a lack of understanding among parents who take payment seriously and fear it.

______________________________________________________________________

Code: Challengs of the pilot_SS_CBHI: responding to service improvement expectations {1-0}

P19: KII_Head of  health Office_ treatment.docx - 19:33 [Similarly, their expectation r..]  (63:63)   (Super)
Codes:	[Challengs of the pilot_SS_CBHI: responding to service improvement expectations] [Suggestions to improve the pilot_SS_CBHI: ensure availability] [Suggestions to improve the pilot_SS_CBHI: respond to expecttions from clients] 
No memos

Similarly, their expectation regarding the availability and quality of services are high. Our fear is only availability of supplies and the way health workers welcome and treat clients. When I say availability of the drugs, it is not at health facilities or model pharmacy found in our . We fear availability of drugs at health facilities which has agreement with our office. Thus, we have to work hard to meet the expectation of the community.

______________________________________________________________________

Code: Challengs of the pilot_SS_CBHI: rsistance from high level HHs {3-0}

P11: KII _Town HO head_ Treatment.docx - 11:9 [The major problem faced with C..]  (25:25)   (Super)
Codes:	[Challengs of the pilot_SS_CBHI: rsistance from high level HHs] [Gaps of the pilot_SS_CBHI: startification criteria] [Gapsof the pilot_SS_CBHI: awareness] 
No memos

The major problem faced with CBHI, as this category started this year, the challenge we faced was that the households categorized under high income tried to pay the amount of middle income. So to avoid such type, awareness creation on the payment difference, clearing implementing the criteria set has to be informed for community and the collection money should be improved. 

P18: KII_CBHI focal__ treatment.docx - 18:3 [The main reason is that the ma..]  (9:9)   (Super)
Codes:	[Challengs of the pilot_SS_CBHI: mis-stratification] [Challengs of the pilot_SS_CBHI: rsistance from high level HHs] 
No memos

The main reason is that the majority of the households identified as higher did not believe and accept their level and denied to pay the required amount. During stratification, we used so many criteria like land, cattle, and other sources of income. However, still there are so many complains especially from identified high-level households. To be honest, there might be misclassification 

P20: KII_Kebele manager__treatment.docx - 20:15 [This question raised from high..]  (21:21)   (Super)
Codes:	[Challengs of the pilot_SS_CBHI: rsistance from high level HHs] 
No memos

This question raised from higher level household; they have information that other s collect the same amount of payment regardless of households' economic status. The majority of households identified as high level do not want to accept their level and also reluctant to perform the payment complaining that they are not different from households identified as the medium level payers. To solve this problem, it is crucial to expand the new payment system [sliding scale] to all s. 

______________________________________________________________________

Code: Challengs of the pilot_SS_CBHI: screening process {1-0}

P20: KII_Kebele manager__treatment.docx - 20:29 [Another thing that can challen..]  (35:35)   (Super)
Codes:	[Challengs of the pilot_SS_CBHI: screening process] 
No memos

Another thing that can challenge this initiative from my experience is the process of screening households in to different level; this thing needs adequate time and known standing committee who have good training and also who knows the community well.

______________________________________________________________________

Code: Challengs of the pilot_SS_CBHI: suspecting service improvement {1-0}

P19: KII_Head of  health Office_ treatment.docx - 19:21 [For example, our community was..]  (47:47)   (Super)
Codes:	[Challengs of the pilot_SS_CBHI: differences among s] [Challengs of the pilot_SS_CBHI: suspecting service improvement] 
No memos

For example, our community was saying, all people in our neighbor  are paying equal amount of money for CBHI membership, why it is different in our . We were also creating awareness to the community that with the previous approach (flat scale), we can't achieve the goal of health insurance which is a culture of social support. The community were also not refusing to pay, rather they were requesting for the availability of services at health facilities. 

______________________________________________________________________

Code: Challengs of the pilot_SS_CBHI: time consuming {1-0}

P18: KII_CBHI focal__ treatment.docx - 18:8 [Only three or four kebeles had..]  (11:11)   (Super)
Codes:	[Challengs of the pilot_SS_CBHI: involvement of health workers] [Challengs of the pilot_SS_CBHI: time consuming] 
No memos

Only three or four kebeles had structure and supported as in screening process. In those kebeles, kebele leaders and kebele managers participated in screening process. Due to the lack of a kebele structure, health workers handled the majority of all procedures, from screening to money collection. This process [screening and collecting payments] is a major activity in implementing sliding scale, which needs time, and enough human resource. Even we recorded it is a major challenge we faced in implementation process in our woreda.

______________________________________________________________________

Code: Challengs of the pilot_SS_CBHI: transparency of premium {1-0}

P17: KII_CBHI Focal__ treatment.docx - 17:13 [The other challenge was that h..]  (27:27)   (Super)
Codes:	[Challengs of the pilot_SS_CBHI: awareness] [Challengs of the pilot_SS_CBHI: premium collection methods] [Challengs of the pilot_SS_CBHI: transparency of premium] 
No memos

The other challenge was that households receive a number voucher/receipt at a time (hawaasni al-tokkotti nagahee hedduu akka kutan gaafatamuu). CBHI is linked to a number of voucher. They were saying that we have interest to pay for CBHI even more amount of money (e.g.3000 birr). But due to linkage to receipts, we refuse/agitate to pay for CBHI too. Most of the time, households don't know the amount of money they paid for CBHI as it is summed up with other vouchers. This also give opportunity for cheating as the household may not know for which services they paid. Indeed, sometimes those who collect the premium desire to visit home to home than collecting the money at office to facilitate for cheating.

______________________________________________________________________

Code: Challengs of the pilot_SS_CBHI: urgency {3-0}

P17: KII_CBHI Focal__ treatment.docx - 17:10 [This was the main gap in the C..]  (27:27)   (Super)
Codes:	[Challengs of the pilot_SS_CBHI: awareness] [Challengs of the pilot_SS_CBHI: urgency] 
No memos

This was the main gap in the CBHI premium collection of this year. It was a new approach for our  to categorize the households based on their economy. But as the time/schedule was too tight, the communities were not well informed about the categories and criteria of categorization. 

P17: KII_CBHI Focal__ treatment.docx - 17:11 [During our meeting with projec..]  (27:27)   (Super)
Codes:	[Challengs of the pilot_SS_CBHI: coordination] [Challengs of the pilot_SS_CBHI: urgency] 
No memos

During our meeting with project central team, we were informed that we will get plenty time for creating awareness of the community. However, later on we informed to complete the premium collection processes with similar schedule to other s that use flat scale approach. Thus, due to a shortage of time schedule, people were not well informed about the issue. This creates resistance even among some of the Ganda level committee members.

P18: KII_CBHI focal__ treatment.docx - 18:6 [The process we passed through ..]  (11:11)   (Super)
Codes:	[Challengs of the pilot_SS_CBHI: urgency] 
No memos

The process we passed through to realize this payment system was so difficult; we only got the one-day orientation at Adama city and given only two months to select screening committee, establishing screen criteria, inform the community and collect money. 

______________________________________________________________________

Code: Challengs of the pilot_SS_CBHI: workload {1-0}

P18: KII_CBHI focal__ treatment.docx - 18:7 [The screening was performed by..]  (11:11)   (Super)
Codes:	[Challengs of the pilot_SS_CBHI: absence of kebele structure] [Challengs of the pilot_SS_CBHI: workload] 
No memos

The screening was performed by our health workers due to inexistence of kebele structure in our woreda. This screening was performed by moving home to home throughout all kebeles in our , which was a huge burden for our staff and was taken a long time to complete. 

______________________________________________________________________

Code: Challengs of the pilot_SS_CBHI:medicine unavailability {1-0}

P15: KII PHCU Leader___TREATMENT TW.docx - 15:14 [In my opinion, the main obstac..]  (34:34)   (Super)
Codes:	[Challengs of the pilot_SS_CBHI: government budget] [Challengs of the pilot_SS_CBHI:medicine unavailability] 
No memos

In my opinion, the main obstacle in the beginning is the lack of medicines. Next, the budget is allocated equally to different levels of health centers, which causes difficulties in providing services. This means that the government allocates 300,000 ETB for grade 'A' health centers, equal to grade 'B' and 'C' health centers. This has a serious problem because a grade 'A' health center serves a large population and there is a shortage of medicines. In contrast, it causes the waste of medicines in grade 'B' and 'C' health centers.

______________________________________________________________________

Code: Claim process: transparent {0-0}
______________________________________________________________________

Code: Community Worries: stress about referal {1-0}

P 6: FGdD_Female_ treatment.docx - 6:24 [There is a shortage of medicin..]  (148:148)   (Super)
Codes:	[Community Worries: stress about referal] [Service gap: inadequate professionals] [Service gap: Medicine unavailability ingovernment facility] [Service gap: Un availability of Laboratory and diagnostic service] 
No memos

There is a shortage of medicine, second if we have enough doctors in the Ganda, and adequate laboratory services are there we do not worry about the services. When he goes for service, he worries about getting service and is stressed about a referral.  If a strong laboratory, enough workers, and medicine, we will be fully confident in insurance. 

______________________________________________________________________

Code: Complaign hearing method: community discussion {1-0}

P 9: KII_ __Cluster_CBHI Desk KII.docx - 9:26 [what is the main thing, our of..]  (56:56)   (Super)
Codes:	[Complaign hearing method: community discussion] [Complaign hearing method: grevance redressal office] 
No memos

what is the main thing, our office has its own department called Appeals and Grievance Redressal Program. Regarding this petition and complaints, what is my job? In each health facility, people of my own will receive reports when people have complaints or problems. There is the focal person, and there are the registration forms. After that, there will be a community discussion again. Community discussion happens every now and then. So, the society is asked. It means that one to thirty to forty people who gather at health facilities and come for service will be asked a question.

______________________________________________________________________

Code: Complaign hearing method: grevance redressal office {1-0}

P 9: KII_ __Cluster_CBHI Desk KII.docx - 9:26 [what is the main thing, our of..]  (56:56)   (Super)
Codes:	[Complaign hearing method: community discussion] [Complaign hearing method: grevance redressal office] 
No memos

what is the main thing, our office has its own department called Appeals and Grievance Redressal Program. Regarding this petition and complaints, what is my job? In each health facility, people of my own will receive reports when people have complaints or problems. There is the focal person, and there are the registration forms. After that, there will be a community discussion again. Community discussion happens every now and then. So, the society is asked. It means that one to thirty to forty people who gather at health facilities and come for service will be asked a question.

______________________________________________________________________

Code: Complaign hearing method:continous monitoring {1-0}

P 9: KII_ __Cluster_CBHI Desk KII.docx - 9:27 [What the problem they have wit..]  (56:56)   (Super)
Codes:	[Complaign hearing method:continous monitoring] 
No memos

What the problem they have with the drug supply, ethics, starting from the guard, starting from the entrance to the health facilities, until they get the service, until they get the pharmacy, until they get the laboratory, until they get out. What is the problem, then take points from the raises they raise and who create the problem? This is done where the director of the health center is

______________________________________________________________________

Code: Complain on Excluded benefit package: {1-0}

P 3: FGD_Men__April 08,2024_.docx - 3:28 [why some services like acciden..]  (115:115)   (Super)
Codes:	[Complain on Excluded benefit package:] 
No memos

why some services like accidents, trauma, motor accidents, machine related accidents, are excluded from insurance services. Despite being a member of the insurance program, I was denied free medical service last year when I accidentally sustained an injury from a machine.

______________________________________________________________________

Code: Concer: CBHI may fail due to lower poremium {1-0}

P 9: KII_ __Cluster_CBHI Desk KII.docx - 9:2 [I am concerned that the insura..]  (22:22)   (Super)
Codes:	[Concer: CBHI may fail due to lower poremium] [Premium : requiere revision] 
No memos

I am concerned that the insurance may be canceled tomorrow, so perhaps this matter should be carefully studied in the next revision of the price, and I do not think it is enough.

______________________________________________________________________

Code: Concern: {1-0}~

P 8: KII HEW_SCH__TREATMENT_TW.docx - 8:9 [Absolutely, this CBHI should c..]  (36:36)   (Super)
Codes:	[Concern:] [Suggestions to improve the cbhi :  avail  adequate drugs] 
No memos

Absolutely, this CBHI should continue. In order for our community to enjoy becoming a member, necessities such as medicines must be properly met and services must be available nearby. On the part of CBHI members, we face many problems. Because we are the ones who collect money from them and make them members of CBHI, the community fights us when there is a lack of services.

______________________________________________________________________

Code: creating sense of ownership {1-0}

P 9: KII_ __Cluster_CBHI Desk KII.docx - 9:43 [CBHI is now especially concern..]  (80:80)   (Super)
Codes:	[creating sense of ownership] 
No memos

CBHI is now especially concerned with the cost of living, which is being collected from businesspersons, and people are being made to join this service willingly which is good. Maybe if we need the sustainability of CBHI program people should pay the money out of their pockets in what manner it is. One principle of CBHI is to create a spirit of ownership. So, a person who is the owner of this thing, and a person who is not an owner, create a very different situation to manage it.  It will be sensitive if it is the owner. They work hard to make it grow, adjust to make everything easy

______________________________________________________________________

Code: delay referal {1-0}

P 2: FGD_Men___TREATMENT.docx - 2:22 [Regarding the provision of ser..]  (147:147)   (Super)
Codes:	[delay referal] [Poor reception of health workers:] [Service gap: Medicine unavailability ingovernment facility] 
No memos

Regarding the provision of service, there are a lot of problems. There are many problems, many…many. For example in the month of September of this year, a pregnant mother bleeding like sheep on the floor and they allowed this lady to go by standing simply by hugging her hand when she transferred from one room to another room. It's better if they ask for assistance from others to carry this mother. Really, I cover her body with clothes ashamed of their job.  Complaints are always raised by people. But no one can pay attention to the complaints. Even though there is appraised work done on insurance, but there are many and always problems in service provisions. The community always...always… always... Raised service problems. The major community complaint on the government is that always raised in every meeting is service providers. Is that the government can't has no capacity to avail medicine? Let alone, he (the government) can't afford and is short of medicine, why workers don't properly and timely perform their job? Why they did not refer patients timely where the medicine was available rather than waiting for them the whole day and finally telling us about the unavailability of medicine? The 100% of community complaints remained without a solution to kebele level as already mentioned. 

______________________________________________________________________

Code: Denial of service : the new kids or family photo is not on the card {2-0}

P 2: FGD_Men___TREATMENT.docx - 2:38 [From family members, Except th..]  (186:187)   (Super)
Codes:	[Denial of service : the new kids or family photo is not on the card] 
No memos

 From family members, Except those listed in the insurance card, no one can get service. For instance, if the family members have five their photos are on their card and get service if not they do not get it. but if you get a child after its renewal, it is requested to bring the kid's photo to add to family members. Whether a new newborn or a missed member of the family cannot get service for example if the family member is 8, and those on the Insurance card are 5, only five of the family members can get service. If the family members including wife and child get service if their photo and name are there on their card. 
P4: If women and children are members of insurance in the family they can get service but if their photo is on the insurance card they are not service as it already said. For example, for my new kid as she was not included in the family insurance, treated her two times by paying from my pocket.

P 5: FGD_Women__treatment.docx - 5:20 [a newborn baby who born after ..]  (138:138)   (Super)
Codes:	[Denial of service : the new kids or family photo is not on the card] [Newborn challenge: Cannot use the service until the next tound] 
No memos

a newborn baby who born after the premium collection can't be member of health insurance until the next round of premium collection schedule. This prone newborns to death. It should be allowed for newborns to be member of health insurance at any time regardless of the fixed premium collection period. Thus, such issue need to be modified.

______________________________________________________________________

Code: Denial of service : the new kids photo is not on the card {1-0}~

P 2: FGD_Men___TREATMENT.docx - 2:29 [The recommendation, I have on ..]  (164:164)   (Super)
Codes:	[Denial of service : the new kids photo is not on the card] 
No memos

The recommendation, I have on the improvement of health is that it has to be pre-planned and notification of delivery of service on the day of photo taken, pay, etc. I had one kid after I renewed my insurance but treated my kid two times with my money regardless of insurance due to my kid's photo was not on the insurance card. They told me that 'there was no her photo on this insurance' and I asked the health extension to add the photo of my kid to the insurance card. But she said, “Bring your photo by capturing your mobile phone”. However,  many farmers do not have smart photos to take photos. So why she did not carry out her duty properly? Rather than say take a photo by ourselves. So she should prepare a plan for her work as we do not have a camera to take photos. We said that health professionals poorly serve us and also there are problems in properly renewing insurance cards. Such types of activities may lead to complaints and barriers to being a health insurance member. There was a person who came three times and did not get service disappointed and sought to be treated at a private clinic due to bureaucracy on getting an insurance card. So person may be disappointed that this insurance discontinued the insurance.  I heard from a farmer who already plans to stop to pay for insurance. I heard from a farmer (mormi koo haacitu) I swore my neck to a knife,  I never pay for insurance again if they want they can jail me.'   The farmer decides not to pay for insurance I already pay for others but I never pay for insurance. So why the health extension did not has her plan to take photos to renew the insurance card and why she was not strict about her appointment? Those are what the government should improve in having regular renewed. Nobody knows when it will be renewed as an illiterate cannot know by reading on the card that is written.

______________________________________________________________________

Code: Digitalization: to redice fake report {1-0}

P 9: KII_ __Cluster_CBHI Desk KII.docx - 9:45 [I propose to digitize everythi..]  (80:80)   (Super)
Codes:	[Digitalization: to redice fake report] 
No memos

I propose to digitize everything, because if we digitize it, the job will be easier. We can cover all the scams. throw out all fake reports, unwanted things, things that just don't seem like it, and it's necessary to go hard on it. Other initiatives are such as sliding scale, strengthening the pooling and raising the base for these new discoveries. 

______________________________________________________________________

Code: discrimination of cbhi members: give priority for non members {0-0}
______________________________________________________________________

Code: discrimination of insurance members: {0-0}
______________________________________________________________________

Code: discrimination of insurance members: Favors non cbhi {1-0}~

P 8: KII HEW_SCH__TREATMENT_TW.docx - 8:3 [the community suggests that th..]  (20:20)   (Super)
Codes:	[discrimination of insurance members: Favors non cbhi] 
No memos

the community suggests that the health care worker will accommodate them more quickly if they go without CBHI than if they take the CBHI to the health center. According to the community, when they go with CBHI, the health care providers are not willing to treat them. In general, the community complains that the health care providers give priority to those who go for cash treatment over those who go with CBHI.

______________________________________________________________________

Code: discrimination of insurance members: insulted {1-0}~

P 3: FGD_Men__April 08,2024_.docx - 3:14 [I am hypertensive and go to ho..]  (97:97)   (Super)
Codes:	[CBHI_SS challenge: high fee for middle group] [discrimination of insurance members: insulted] 
No memos

 I am hypertensive and go to hospital frequently; I have observed some problems about insurance service especially how health workers treat insurance member. One day a pharmacist insulted insurance members in front of me saying, “Those who are carrying insurance card immediately leave this area for those who can pay money for the medicine they are going to take”. I angrily asked that boy why he spoke like that; I told him that those insurance members also paid money for the government to get enough service from that hospital. Regarding the amount of payment, it is not affordable for some individuals, now people are facing challenges with the increased cost of living, I know some individuals who could not pay for insurance service and excluded from it. You can imagine how it is difficult to pay that much money while cost of everything being increased from time to time. In my opinion, government needs to re-evaluate about the amount of payment, it needs to be revised in a way that it could be affordable for many individuals.

______________________________________________________________________

Code: discrimination of insurance members: Professional hate us {1-0}~

P 3: FGD_Men__April 08,2024_.docx - 3:25 [his is defamation; no one go t..]  (109:109)   (Super)
Codes:	[discrimination of insurance members: Professional hate us] [Poor reception of health workers:] 
No memos

his is defamation; no one go to health facility without illness. Do you believe we go to health facility without illness to face that all challenges? Let me tell you the truth, Insurance members and the beggars sitting in front of church are the same [kadhataan bataskaana fuuldura taa'u fi miseensi insuraansii fayyaa tokkodha]. Once they see that insurance card in your hand, they extremely hate you, they never greet you, and they ignore you at all. No one go to health facility without experiencing some illness or discomfort. Even the problems and experiences we shared with you on this discussion is not enough, you have to go to the rural community and ask them; I am sure many of them will cry while telling you the problems they faced in the health facilities. Rich individuals may not see what we are talking as problem because they have enough money to seek health service from private clinics. I know the problem in detail because I have experienced many challenges before becoming to insurance member and after I became insurance member. I have a child with chronic illness and faced all problems shared here by my friends

______________________________________________________________________

Code: Discrimination of insurance members {3-0}~

P 3: FGD_Men__April 08,2024_.docx - 3:10 [Previously, I was member of he..]  (93:93)   (Super)
Codes:	[Discrulination of insurance members] [long waiting time] [Medicine unavailability in government facility] [PB_CBHI: protect from expensive payment] 
No memos

Previously, I was member of health insurance; however, this year I do not want to be a member, because the service that I could get by this membership is not that much, there is no medicine in the government hospital. Not only medicine; as my friend previously raised, they also not permit X-Ray, and ultrasound services for insurance members. When an insurance member sent to X-Ray or ultrasound room, the individuals work in those room intentionally give extended appointment for the service. Imagine how a person who came from rural area wait three or four days to get these services. They [health workers] give you alternatives; if you can wait for long time, you can get these services free of charge by your insurance service, however, if you want it by the same day the request written, you have to go to the private clinics [mentioned the clinics by name]. If these problems resolved, insurance service is a relief for our community especially our rural community. I have good awareness about insurance service; I have no problem of awareness, I decided to drop out from membership due to inadequate services and mistreatment by health workers for insurance members. Even in my opinion, rural community have good awareness about the benefits of health insurance service. Even I learnt about insurance service from a person who came from rural area to seek health service for his child; fortunately, my child and his child admitted to the same room in one hospital in Addis Ababa, at the time, he was member of health insurance and I was not, and he told me about it in detail. Even he helped me to collect some of expensive medicine for my child using his insurance book. 

P 3: FGD_Men__April 08,2024_.docx - 3:12 [nsurance members at this time ..]  (96:96)   (Super)
Codes:	[Discrulination of insurance members] 
No memos

nsurance members at this time have no respect from health workers; health workers do not treat health insurance members as human beings. Let me tell you what happened to me last month; I experienced severe headache and at midnight and visited a health center, at which they told me my blood pressure raised and referred me to general hospital. After I reached the general hospital, they did not evaluate me as I needed and ordered me to return home informing me I had no any illness. They joked to me, laughed at me saying, “you are fine, go to your home”. In the following day, I visited one private clinic and diagnosed with kidney problem and severe hypertension and referred to  referral hospital. Thanks to the referral hospital, they identified my illness and referred me to st.paul hospital. All staff of the hospital were good for me and treated me well except those who work in pharmacy room; they mistreated me and denied me a medicine after they learned that I was insurance member. They sent me to private pharmacy from which they have share

P 7: FGDs_Men___ treatment.docx - 7:30 [They serve nonmembers first an..]  (170:170)   (Super)
Codes:	[Discrulination of insurance members] 
No memos

They serve nonmembers first and push us backwards. This is the complaint from the health centers. If they continue addressing the complaint the community will not be left behind. They have plans to renew, they renew if the health facility improves there

______________________________________________________________________

Code: Enrollment problem_SS_CBHI: Individual level enrollment >18 years {1-0}

P19: KII_Head of  health Office_ treatment.docx - 19:3 [Previously, the head of the ho..]  (23:23)   (Super)
Codes:	[Enrollment problem_SS_CBHI: Individual level enrollment >18 years] 
No memos

Previously, the head of the household add some percent of payment and register all his/her family member as a member of CBHI even though they are >18 years old. But, currently it is not possible. Family member greater than 18 years old should be independent to be a member of CBHI if they have no especial issues such as mental health problems, disabilities, etc. Thus, even though they have no many to pay, we didn't allow them to use the green card of their family member for those whose age are >= 18 years old. We believe that this important indicator of fairness premium collection in our area. 

______________________________________________________________________

Code: Enrollment problem_SS_CBHI: Individual level enrollmentt {1-0}

P19: KII_Head of  health Office_ treatment.docx - 19:2 [But this year, we were mobiliz..]  (21:21)   (Super)
Codes:	[Enrollment problem_SS_CBHI: Individual level enrollmentt] 
No memos

 But this year, we were mobilizing adolescents/youth greater than 18 years to take green card which indicates they are independent CBHI member even though they are living with their family members.  This, help us to achieve greater than 100 percent. 

______________________________________________________________________

Code: Fairness and transparency_SS_CBHI: the process {2-0}

P13: KII _ _HC-Head _ Ttreatment.docx - 13:4 [Transparency and fairness are ..]  (17:17)   (Super)
Codes:	[Fairness and transparency_SS_CBHI: the process] [Strenght of the pilot_SS_CBHI: Transparency and fairness process] 
No memos

Transparency and fairness are good when insurance contributions are collected. The contributions for CBHI membership is collected by receipt. And people who contribute have a record on it. Even the community knows it (the amount of contribution) and does not pay unless it receives a receipt. Its fairness is also good. It is good because when ranking was done by community structure, together with HEW, and identifies the highest, middle and lowest payers into three categories.

P16: KII__HEW_ _ _.docx - 16:3 [In my opinion it has fairness ..]  (17:17)   (Super)
Codes:	[Fairness and transparency_SS_CBHI: the process] 
No memos

In my opinion it has fairness and transparency. The transparency of it is indicated that we collect it voluntarily, told amount of contribution, not necessarily by force, because we aware them that this thing will benefit you.

______________________________________________________________________

Code: Fairness_SS_CBHI: processes {0-0}
______________________________________________________________________

Code: Gaps of the pilot_SS_CBHI: awareness {1-0}

P12: KII CBHI focal___TREATMENT TW.docx - 12:24 [An understanding of the commun..]  (47:47)   (Super)
Codes:	[Gaps of the pilot_SS_CBHI: awareness] [Gaps of the pilot_SS_CBHI: urgency] 
No memos

An understanding of the community classification criteria was provided to the categorization committee. The committee was made to make categorization based on these criteria. No awareness was given down to the household level during the categorization. However, during the collection of payment, each household was reached and told of his category.

______________________________________________________________________

Code: Gaps of the pilot_SS_CBHI: preparation {1-0}

P11: KII _Town HO head_ Treatment.docx - 11:30 [Those involved in collecting t..]  (68:68)   (Super)
Codes:	[Gaps of the pilot_SS_CBHI: preparation] 
No memos

Those involved in collecting the data have to be trained and get sufficient information about the criteria. For this year we did not give an awareness on the head of implementation. But in the coming year will give before renewing.

______________________________________________________________________

Code: Gaps of the pilot_SS_CBHI: startification criteria {1-0}

P11: KII _Town HO head_ Treatment.docx - 11:9 [The major problem faced with C..]  (25:25)   (Super)
Codes:	[Challengs of the pilot_SS_CBHI: rsistance from high level HHs] [Gaps of the pilot_SS_CBHI: startification criteria] [Gapsof the pilot_SS_CBHI: awareness] 
No memos

The major problem faced with CBHI, as this category started this year, the challenge we faced was that the households categorized under high income tried to pay the amount of middle income. So to avoid such type, awareness creation on the payment difference, clearing implementing the criteria set has to be informed for community and the collection money should be improved. 

______________________________________________________________________

Code: Gaps of the pilot_SS_CBHI: urgency {1-0}

P12: KII CBHI focal___TREATMENT TW.docx - 12:24 [An understanding of the commun..]  (47:47)   (Super)
Codes:	[Gaps of the pilot_SS_CBHI: awareness] [Gaps of the pilot_SS_CBHI: urgency] 
No memos

An understanding of the community classification criteria was provided to the categorization committee. The committee was made to make categorization based on these criteria. No awareness was given down to the household level during the categorization. However, during the collection of payment, each household was reached and told of his category.

______________________________________________________________________

Code: Gapsof the pilot_SS_CBHI: awareness {1-0}

P11: KII _Town HO head_ Treatment.docx - 11:9 [The major problem faced with C..]  (25:25)   (Super)
Codes:	[Challengs of the pilot_SS_CBHI: rsistance from high level HHs] [Gaps of the pilot_SS_CBHI: startification criteria] [Gapsof the pilot_SS_CBHI: awareness] 
No memos

The major problem faced with CBHI, as this category started this year, the challenge we faced was that the households categorized under high income tried to pay the amount of middle income. So to avoid such type, awareness creation on the payment difference, clearing implementing the criteria set has to be informed for community and the collection money should be improved. 

______________________________________________________________________

Code: Health seeking behavior: Insurance mebers are better {1-0}

P 3: FGD_Men__April 08,2024_.docx - 3:24 [In my opinion, economically be..]  (107:107)   (Super)
Codes:	[Health seeking behavior: Insurance mebers are better] [High category needs : better service and avalable medication] 
No memos

In my opinion, economically better individuals protect their health and visit health facilities more than poor individuals. Those who have enough money protect their health; they do not worry about the payment or unavailability of medicine from government hospital as they afford to buy it from outside. To be honest, those who are insurance member also travel to health facility than those who are not members; they do this believing that they may get the medicine one day. For example, if they do not get their medicine today, they may go to the same health facility the next day to check if that medicine advises.

______________________________________________________________________

Code: Health seeking behavior: poor {3-0}~

P 1: _FGD_WOMEN_TREATMENT_TW.docx - 1:27 [Our community has difficulty i..]  (124:124)   (Super)
Codes:	[Health seeking behavior: poor] 
No memos

Our community has difficulty in seeking treatment timely. I started getting sick two days ago and haven't been to the health facility yet. The reason I didn't go to the clinic for treatment is because I thought it would leave me on its own and I used traditional local treatments.

P 1: _FGD_WOMEN_TREATMENT_TW.docx - 1:28 [Despite the shortage of medici..]  (125:125)   (Super)
Codes:	[Health seeking behavior: poor] [Service gap: Medicine unavailability ingovernment facility] [Service gap: Poor reception of health workers: denial for repeat service] 
No memos

Despite the shortage of medicines and health professional problems, CBHI is very good. However, we have a serious shortcoming in health seeking behaviour

P 2: FGD_Men___TREATMENT.docx - 2:34 [From my experience regarding h..]  (176:176)   (Super)
Codes:	[Health seeking behavior: poor] 
No memos

From my experience regarding health-seeking behavior I could not go for health care at the first step unless it severe case. When I was ill,  I ate chopped onion with chill and headache, and pneumonia (michi) improved me. This is my personal experience. 


______________________________________________________________________

Code: Health seeking behavior: poor for two reasons: {1-0}~

P 2: FGD_Men___TREATMENT.docx - 2:32 [Regarding health-seeking behav..]  (173:173)   (Super)
Codes:	[Health seeking behavior: poor for two reasons:] [no one can be fully confident in insurance without having money in his pocket because finally he will sent to private] 
No memos

 Regarding health-seeking behavior, there is a difference between the previous trend and the current. The difference, in the previous people took traditional drugs. For example, if someone is ill, He may drink called the so-called 'Bokkolluu'. In the past, if someone drank this Bokkollu cured immediatily. But now if someone drinks it he cannot cured because everything/climate has changed. But it is not working now. For example, my father let him rest in peace, when sick he drank two cups of Bokkollu and then cured immediately and took a bath. Some who were exposed to pneumonia (dhukkuba michii) drank it and saw results immediately. But now if you drink several cups, no improvement due to climate change rather than looking for a health facility. For this reason, every individual must look for health facilities because if he drinks the bokkolluu no change. He cures when he goes to a health facility. b/c of this they go for health facility. However, their health-seeking behavior is not immediate after ill. They stayed from two to three days before health seeking. The reasons are related to two things. First,  Perception toward health facilities. He says 'If I go for  healthcare, they provide me drugs simply by asking about my problems orally without further investigation and result in not addressing my disease.' So he delayed to health seek as he assumed no value in going there. The second for the delay is the perception that even if  I go to a public health facility using health insurance I can't get anything and back to the private clinic so He may be delayed in for searching money used to be treated at a private clinic. So he delayed the until he got money for treatment. I tell you the truth if someone is sick,  no one can be fully confident in insurance without having money in his pocket because finally he will sent to private.

______________________________________________________________________

Code: health workers are cooperative {1-0}

P 1: _FGD_WOMEN_TREATMENT_TW.docx - 1:5 [In my opinion, the health prof..]  (93:93)   (Super)
Codes:	[health workers are cooperative] 
No memos

 In my opinion, the health professionals tried to help me when I was very sick

______________________________________________________________________

Code: health workers unvailable {1-0}

P 2: FGD_Men___TREATMENT.docx - 2:27 [The problem related to not ren..]  (160:160)   (Super)
Codes:	[health workers unvailable] [Service gap: Medicine unavailability ingovernment facility] 
No memos

The problem related to not renewing was that for example, we come here three times but we cannot get workers. The second problem was they said this insurance did not benefit us because we paid for renewed in one and bought drugs from a private in another then decided to be treated at a private clinic. 

______________________________________________________________________

Code: high category  member:  majority are members {1-0}

P 6: FGdD_Female_ treatment.docx - 6:13 [most of the higher-income are ..]  (136:136)   (Super)
Codes:	[high category  member:  majority are members] 
No memos

most of the higher-income are members of health insurance. 

______________________________________________________________________

Code: high category  member:  need only quality service not amount of payment {1-0}

P 3: FGD_Men__April 08,2024_.docx - 3:21 [Regarding screening process, I..]  (103:103)   (Super)
Codes:	[CBHI_SS challenge: high fee for middle group] [high category  member:  need only quality service not amount of payment] [Weakness and challenges SS-CBHI implementation(stratification) : no information provided] 
No memos

Regarding screening process, I have no information about who did it, and how they did it. We only informed our level and told how much to pay. I want to give some advice for you and the government on the amount of payment, especially concerning those who identified as medium level and asked to pay 1300 birr; many individuals indented as medium and supposed to pay 1300 birr cannot pay even 500 birr, majority of them have nothing as property. Rich individuals have no worry about the amount of payment, rather they complain about unavailability of medicines. In the future, government should find the mechanism to support those who identified as medium and nothing to pay. I know the government supports poor households [indigent HHs], which is very good; however, it is good if the quota of those supported by the government increased to include individuals identified in medium level. Regarding the increment of insurance payment from time to time, I have no complain as the price of medicine also being increased from time to time. If we pay the same amount each year while the price of medicine increases, how do you think the government able to provide those medicines for us?

______________________________________________________________________

Code: High category needs : better service and avalable medication {1-0}

P 3: FGD_Men__April 08,2024_.docx - 3:24 [In my opinion, economically be..]  (107:107)   (Super)
Codes:	[Health seeking behavior: Insurance mebers are better] [High category needs : better service and avalable medication] 
No memos

In my opinion, economically better individuals protect their health and visit health facilities more than poor individuals. Those who have enough money protect their health; they do not worry about the payment or unavailability of medicine from government hospital as they afford to buy it from outside. To be honest, those who are insurance member also travel to health facility than those who are not members; they do this believing that they may get the medicine one day. For example, if they do not get their medicine today, they may go to the same health facility the next day to check if that medicine advises.

______________________________________________________________________

Code: high category not meber:  no medicine {1-0}

P 6: FGdD_Female_ treatment.docx - 6:18 [Sometimes there is the assumpt..]  (140:140)   (Super)
Codes:	[high category not meber:  no medicine] [high category not meber: long waitingbtime] [highcateg not member: bureaucracy] 
No memos

Sometimes there is the assumption that there challenge to becoming a member of insurance especially for those has enough wealth they have enough wealth, they say, “If I come here (to the public facility), I cannot get medicine; I cannot kill my time; there are long queue; bureaucracy I have to go to a private clinic and get service by paying my money” so it is impossible to say 100% has members of health insurance

______________________________________________________________________

Code: high category not meber:  Poor quality of service {1-0}~

P 5: FGD_Women__treatment.docx - 5:13 [The other reason is due to poo..]  (129:129)   (Super)
Codes:	[high category not meber:  Poor quality of service] [Premium contribution: too small compared to private facility service] 
No memos

The other reason is due to poor quality of health services at public health facilities. For instance, last year, I visited a public health facility after I got sick. They didn't assessed my illness very well. They gave me drugs with wrong diagnosis. I slept my home for about two months. Finally, I went to private clinic, I paid eight thousand birr. The private clinic told me that my illness was related to pregnancy. But the public facility even didn't tell me as I was pregnant. Thus, due to poor quality services at public facilities, people despair for health insurance and refuse to renew the health insurance card. I used to be member of health insurance. This year, however, I refused to renew. I had money but I refused to pay for the health insurance because the health services they provide has no quality. People pay premium but do not get health services. Because of this they decide to dropout from the membership. For those who are not member of health insurance, the health workers provide the all necessary services including laboratory investigation because the health facility need money in cash. 

______________________________________________________________________

Code: high category not meber: long waitingbtime {1-0}

P 6: FGdD_Female_ treatment.docx - 6:18 [Sometimes there is the assumpt..]  (140:140)   (Super)
Codes:	[high category not meber:  no medicine] [high category not meber: long waitingbtime] [highcateg not member: bureaucracy] 
No memos

Sometimes there is the assumption that there challenge to becoming a member of insurance especially for those has enough wealth they have enough wealth, they say, “If I come here (to the public facility), I cannot get medicine; I cannot kill my time; there are long queue; bureaucracy I have to go to a private clinic and get service by paying my money” so it is impossible to say 100% has members of health insurance

______________________________________________________________________

Code: high category not member:  no drugs available {1-0}

P 3: FGD_Men__April 08,2024_.docx - 3:17 [When it comes to rich individu..]  (99:99)   (Super)
Codes:	[high category not member:  no drugs available] 
No memos

When it comes to rich individuals, always they say that they do not want to become member due to unavailability of medicines. As they have no financial problem, they prefer to go to private health facilities.

______________________________________________________________________

Code: high category: prefer private {1-0}

P 6: FGdD_Female_ treatment.docx - 6:32 [: the middle level of the comm..]  (157:157)   (Super)
Codes:	[high category: prefer private] 
No memos

: the middle level of the community is more engaged in insurance. The higher category may not worry as the middle level as he may treated privately in case of sickness

______________________________________________________________________

Code: highcateg not member: bureaucracy {1-0}

P 6: FGdD_Female_ treatment.docx - 6:18 [Sometimes there is the assumpt..]  (140:140)   (Super)
Codes:	[high category not meber:  no medicine] [high category not meber: long waitingbtime] [highcateg not member: bureaucracy] 
No memos

Sometimes there is the assumption that there challenge to becoming a member of insurance especially for those has enough wealth they have enough wealth, they say, “If I come here (to the public facility), I cannot get medicine; I cannot kill my time; there are long queue; bureaucracy I have to go to a private clinic and get service by paying my money” so it is impossible to say 100% has members of health insurance

______________________________________________________________________

Code: hospital service not acceessable {2-0}

P 4: FGD_MEN_ treatment.docx - 4:20 [As a community in our ..]  (141:141)   (Super)
Codes:	[hospital service not acceessable] 
No memos

As a community in our , it is difficult to access hospital level services due to distance. Our patients are suffering because there is no hospital in our . So we need a hospital in our .  So it would be great if there is a hospital nearby. The people raise such complaints.

P 4: FGD_MEN_ treatment.docx - 4:29 [There should be a hospital nea..]  (156:156)   (Super)
Codes:	[hospital service not acceessable] 
No memos

There should be a hospital nearby to improve health insurance services. We do not have a hospital in this  and when we need hospital services we are using neighboring s like _ and Meta. Therefore, we need a hospital in our  to get hospital services nearby.

______________________________________________________________________

Code: how high coverage: {0-0}
______________________________________________________________________

Code: HWB: respect {1-0}~

P 1: _FGD_WOMEN_TREATMENT_TW.docx - 1:6 [Later, however, I learned that..]  (95:95)   (Super)
Codes:	[HWB: respect] [PB_ss_CBHI: good package of service] [Service gap: Health worker behaviour; no respect] 
No memos

Later, however, I learned that it was useful when I started using it and saw those who had used it even for the surgical treatment. So this CBHI is very useful. With regard to professional reception, not everyone has the same characteristics. This means that there are those who respect others and those who do not.

______________________________________________________________________

Code: Impact_SS_CBH: access gap with nonmembers {1-0}

P16: KII__HEW_ _ _.docx - 16:15 [Therefore, CBHI membership has..]  (33:33)   (Super)
Codes:	[Impact_SS_CBH: access gap with nonmembers] 
No memos

Therefore, CBHI membership has a significant impact on the health care seeking practices of our community. A person who is not a member of community based health insurance may have no money when he/she gets sick and will have difficulty getting the treatment/care he/she needs.

______________________________________________________________________

Code: Impact_SS_CBH: commy health status {3-0}

P14: KII HEW__2024.docx - 14:23 [In the past, how many people d..]  (38:38)   (Super)
Codes:	[Impact_SS_CBH: commy health status] [Impact_SS_CBH: timely access] 
No memos

In the past, how many people died because of lack of this treatment and lack of money. Now, as I told you earlier, the insurance has gone and he/she is being treated wonderfully. I mean the present. Even for a headache, the community is not lying on the floor like they used to and dying now. It means that our farmer is going there in the early hours using insurance card and returning to the development in the afternoon. I mean, it's very useful.

P15: KII PHCU Leader___TREATMENT TW.docx - 15:11 [In addition to providing quali..]  (28:28)   (Super)
Codes:	[Benifits_SS_CBHI: Access] [Impact_SS_CBH: commy health status] 
No memos

In addition to providing quality services as our health center, we have been providing health education every working day at health centers, schools, and public gatherings. As a result, our community has good health service-seeking behaviors. For example, at this health center, we provide delivery services to 70 to 100 mothers per month.

P19: KII_Head of  health Office_ treatment.docx - 19:23 [We have been receiving feedbac..]  (49:49)   (Super)
Codes:	[Impact_SS_CBH: commy health status] [Impact_SS_CBHI: empowered women and chidren] 
No memos

We have been receiving feedback from the community that the health insurance highly increases the health seeking behavior of women and children as most of the time women and children do not seek treatment due to lack of money.

______________________________________________________________________

Code: Impact_SS_CBH: deteriorate quality {1-0}

P13: KII _ _HC-Head _ Ttreatment.docx - 13:11 [The CBHI has an impact with in..]  (31:31)   (Super)
Codes:	[Impact_SS_CBH: deteriorate quality] [Impact_SS_CBHI: Unreliable finance] 
No memos

The CBHI has an impact with inflation. If the contribution and price of the drug balance each other, it will have a good impact. If the insurance contribution is low, it will have a negative impact. Because if the contribution is small, the price of medicines will be expensive and major procedures can be done in hospitals. In this case the CBHI has negative impact on health service provision.

______________________________________________________________________

Code: Impact_SS_CBH: empower family through women empowerement {1-0}

P14: KII HEW__2024_docx - 14:24 [The CBHI membership promotes w..]  (40:40)   (Super)
Codes:	[Impact_SS_CBH: empower family through women empowerement] [Impact_SS_CBHI: empowered women and chidren] 
No memos

The CBHI membership promotes women's and children care seeking and access to medical care, because health insurance is on women, families and women as I just told you. The husband is unaware. Most of the family is accompanied by a woman. It means she doesn't wait for money to search, because she has insurance. In the past, there was already someone who waits until the baby dies due to lack of money. Women are paying for the insurance premium contributions before their husbands.

______________________________________________________________________

Code: Impact_SS_CBH: family health {1-0}

P17: KII_CBHI Focal__ treatment.docx - 17:38 [Thus, any family member who ge..]  (51:51)   (Super)
Codes:	[Impact_SS_CBH: family health] [Impact_SS_CBH: maternal and child morbidity and mortality] 
No memos

Thus, any family member who get sick visit health facility using the green card without expectation and arrangement for searching money. Even if the head of the household is not around, anyone who get sick especially children and women visit health facilities without requesting money from their father/husband. In general, it is clear that health insurance highly contribute in the reduction of maternal and child morbidities and mortalities. 

______________________________________________________________________

Code: Impact_SS_CBH: high health seeking vs nonmebres {1-0}

P20: KII_Kebele manager__treatment.docx - 20:24 [Yes of course, as I told you, ..]  (31:31)   (Super)
Codes:	[Impact_SS_CBH: high health seeking vs nonmebres] 
No memos

Yes of course, as I told you, CBHI members can get some services freely while non-CBHI individuals should pay for all services they get from health facility. CBHI members do not fear payment to go to health facility, though they may think that they cannot get all medicines from that health facility. 

______________________________________________________________________

Code: Impact_SS_CBH: maternal and child morbidity and mortality {1-0}

P17: KII_CBHI Focal__ treatment.docx - 17:38 [Thus, any family member who ge..]  (51:51)   (Super)
Codes:	[Impact_SS_CBH: family health] [Impact_SS_CBH: maternal and child morbidity and mortality] 
No memos

Thus, any family member who get sick visit health facility using the green card without expectation and arrangement for searching money. Even if the head of the household is not around, anyone who get sick especially children and women visit health facilities without requesting money from their father/husband. In general, it is clear that health insurance highly contribute in the reduction of maternal and child morbidities and mortalities. 

______________________________________________________________________

Code: Impact_SS_CBH: negative impact on quality {1-0}

P18: KII_CBHI focal__ treatment.docx - 18:17 [if we look at the last year's ..]  (23:23)   (Super)
Codes:	[Impact_SS_CBH: negative impact on quality] 
No memos

if we look at the last year's scenario, health facilities in our s could not purchase medicines due to lack of budget; the collected budget was used up and our account was empty after two quarters. Red Cross pharmacy and hospital also cancelled their contracts with our  after we failed to pay the amount of budget they complained.

______________________________________________________________________

Code: Impact_SS_CBH: service quality detrerioration {3-0}

P18: KII_CBHI focal__ treatment.docx - 18:22 [As I explained earlier, we wer..]  (29:29)   (Super)
Codes:	[Impact_SS_CBH: service quality detrerioration] [Impact_SS_CBH: unreliable financial system] 
No memos

As I explained earlier, we were near to stop services due to inability to pay the complained budget for health facilities. All of our four health center did not get their 2015 payment due to lack of budget. Initially, our plan was to increase the amount of contribution from 410 to 960 birr to pay last year's credit and to support health facilities. 

P20: KII_Kebele manager__treatment.docx - 20:3 [As you know, CBHI began many y..]  (5:5)   (Super)
Codes:	[Impact_SS_CBH: service quality detrerioration] [Service gap_SS_CBHI: medicine availability] 
No memos

As you know, CBHI began many years ago and the majority of our community benefited from it; medicines were available before years; however, in recent years, both health centers and hospitals could not provide the expected level of services for our community; you cannot get the medicines you want from health centers and hospitals at this time. 

P20: KII_Kebele manager__treatment.docx - 20:21 [Medicine is the basic thing th..]  (27:27)   (Super)
Codes:	[Impact_SS_CBH: service quality detrerioration] 
No memos

Medicine is the basic thing that our community need from health facilities; as I told you before, health centers cannot avail medicine for our community at all; they send our community to Red Cross pharmacy or private pharmacy; therefore, for me, the quality of health service is compromised due to CBHI service.

______________________________________________________________________

Code: Impact_SS_CBH: stable family {1-0}

P14: KII HEW__2024_.docx - 14:14 [The housewife takes the card t..]  (27:27)   (Super)
Codes:	[Impact_SS_CBH: stable family] [Impact_SS_CBH: Women impowrement] 
No memos

The housewife takes the card to the hospital if her family is sick. She does not ask her husband for money. She has a health insurance card and takes it to the health center. How many people have been divorced because of this before?   Now that the insurance has come, she puts the card in her pocket and goes for care.

______________________________________________________________________

Code: Impact_SS_CBH: timely access {2-0}

P14: KII HEW__2024_.docx - 14:23 [In the past, how many people d..]  (38:38)   (Super)
Codes:	[Impact_SS_CBH: commy health status] [Impact_SS_CBH: timely access] 
No memos

In the past, how many people died because of lack of this treatment and lack of money. Now, as I told you earlier, the insurance has gone and he/she is being treated wonderfully. I mean the present. Even for a headache, the community is not lying on the floor like they used to and dying now. It means that our farmer is going there in the early hours using insurance card and returning to the development in the afternoon. I mean, it's very useful.

P16: KII__HEW_ _ _.docx - 16:14 [ecause when they are sick, the..]  (33:33)   (Super)
Codes:	[Impact_SS_CBH: timely access] [Impact_SS_CBH:Utilization] 
No memos

ecause when they are sick, they have community based health insurance and enjoy quality services when they need it. If you are a member of community based health insurance, you have insurance and you can go to the hospital/health center when you are sick and get the services you need free of charge.

______________________________________________________________________

Code: Impact_SS_CBH: unreliable financial system {2-0}

P18: KII_CBHI focal__ treatment.docx - 18:22 [As I explained earlier, we wer..]  (29:29)   (Super)
Codes:	[Impact_SS_CBH: service quality detrerioration] [Impact_SS_CBH: unreliable financial system] 
No memos

As I explained earlier, we were near to stop services due to inability to pay the complained budget for health facilities. All of our four health center did not get their 2015 payment due to lack of budget. Initially, our plan was to increase the amount of contribution from 410 to 960 birr to pay last year's credit and to support health facilities. 

P20: KII_Kebele manager__treatment.docx - 20:22 [I heard that health centers al..]  (27:27)   (Super)
Codes:	[Impact_SS_CBH: unreliable financial system] 
No memos

I heard that health centers also faced a problem of shortage of budget to purchase enough medicine due to the inadequacy of collected money from the community to buy medicine and other necessary things in the health center. 

______________________________________________________________________

Code: Impact_SS_CBH: Women impowrement {1-0}

P14: KII HEW__2024_.docx - 14:14 [The housewife takes the card t..]  (27:27)   (Super)
Codes:	[Impact_SS_CBH: stable family] [Impact_SS_CBH: Women impowrement] 
No memos

The housewife takes the card to the hospital if her family is sick. She does not ask her husband for money. She has a health insurance card and takes it to the health center. How many people have been divorced because of this before?   Now that the insurance has come, she puts the card in her pocket and goes for care.

______________________________________________________________________

Code: Impact_SS_CBH:Utilization {1-0}

P16: KII__HEW_ _ _.docx - 16:14 [ecause when they are sick, the..]  (33:33)   (Super)
Codes:	[Impact_SS_CBH: timely access] [Impact_SS_CBH:Utilization] 
No memos

ecause when they are sick, they have community based health insurance and enjoy quality services when they need it. If you are a member of community based health insurance, you have insurance and you can go to the hospital/health center when you are sick and get the services you need free of charge.

______________________________________________________________________

Code: Impact_SS_CBHI: close the disparity {1-0}

P12: KII CBHI focal___TREATMENT TW.docx - 12:14 [Now, however, since the introd..]  (33:33)   (Super)
Codes:	[Impact_SS_CBHI: close the disparity] [Impact_SS_CBHI: ensure equal access] 
No memos

Now, however, since the introduction of CBHI, women have equal access to health services, just as men and children get timely treatment when they get sick. Last time, everything was managed by the husband, and they waited for the husband's decision to go to a health facility. Now, if they are a CBHI member, they take their membership card and go to the public health facility without delaying to wait for their husband.

______________________________________________________________________

Code: Impact_SS_CBHI: empowered women {2-0}

P11: KII _Town HO head_ Treatment.docx - 11:28 [The importance of health insur..]  (59:59)   (Super)
Codes:	[Impact_SS_CBHI: empowered women] 
No memos

The importance of health insurance for women and children is very highly important. The country's economy depends on males but today if she observes something odd she looks for a health facility without delay. She immediately goes to the health facility without delay. And it possibly decreased maternal and child-related problems.

P20: KII_Kebele manager__treatment.docx - 20:26 [As you know, it is difficult f..]  (33:33)   (Super)
Codes:	[Impact_SS_CBHI: empowered women] [Impact_SS_CBHI: women self reliance] 
No memos

As you know, it is difficult for poor households to seek health services by payment; especially when it comes to women or children, the burden is not easy. Men, most of the time seek health service by selling something like grain or sheep or by borrowing from friends; however, women cannot do this especially when there is limited resource in the home.

______________________________________________________________________

Code: Impact_SS_CBHI: empowered women and chidren {6-0}

P13: KII _ _HC-Head _ Ttreatment.docx - 13:16 [Children often get sick and ar..]  (35:35)   (Super)
Codes:	[Impact_SS_CBHI: empowered women and chidren] [Impact_SS_CBHI: health service equity] 
No memos

Children often get sick and are taken to the hospital quickly if they are insured, and women are treated quickly if they are insured. Children often get sick and go to the hospital early because they have insurance. So, being CBHI membership promotes women's and children care seeking and access to medical care

P14: KII HEW__2024_.docx - 14:24 [The CBHI membership promotes w..]  (40:40)   (Super)
Codes:	[Impact_SS_CBH: empower family through women empowerement] [Impact_SS_CBHI: empowered women and chidren] 
No memos

The CBHI membership promotes women's and children care seeking and access to medical care, because health insurance is on women, families and women as I just told you. The husband is unaware. Most of the family is accompanied by a woman. It means she doesn't wait for money to search, because she has insurance. In the past, there was already someone who waits until the baby dies due to lack of money. Women are paying for the insurance premium contributions before their husbands.

P15: KII PHCU Leader___TREATMENT TW.docx - 15:13 [The CBHI has benefited many mo..]  (32:32)   (Super)
Codes:	[Impact_SS_CBHI: empowered women and chidren] 
No memos

The CBHI has benefited many mothers and children. Currently, if they get sick, they come with only their CBHI cards without wasting time looking for money.

P16: KII__HEW_ _ _.docx - 16:16 [Without this community based h..]  (35:35)   (Super)
Codes:	[Impact_SS_CBHI: empowered women and chidren] [Impact_SS_CBHI: women self reliance] 
No memos

Without this community based health insurance, they will have difficulty getting treatment/care again when they need it. If there is no insurance, women and children cannot go back to the clinic more than once or twice. If they don't have resources, they will suffer a lot. But if they have insurance, they can go for treatment whenever they want. Therefore, being a CBHI member promotes women's and children's care seeking and access to medical care.

P18: KII_CBHI focal__ treatment.docx - 18:29 [This helps them to seek health..]  (35:35)   (Super)
Codes:	[Impact_SS_CBHI: empowered women and chidren] [Impact_SS_CBHI: removes financial barriers] 
No memos

This helps them to seek health service at the time when they have no money; especially, women and children benefitted from this program, as they have no power to sell properties for the purpose of treatment. They can easily pick their membership ID and go to health facility without worrying for payments. CBHI liberates children and women from dependence to seek healthcare. 

P19: KII_Head of  health Office_ treatment.docx - 19:23 [We have been receiving feedbac..]  (49:49)   (Super)
Codes:	[Impact_SS_CBH: commy health status] [Impact_SS_CBHI: empowered women and chidren] 
No memos

We have been receiving feedback from the community that the health insurance highly increases the health seeking behavior of women and children as most of the time women and children do not seek treatment due to lack of money.

______________________________________________________________________

Code: Impact_SS_CBHI: ensure equal access {3-0}

P12: KII CBHI focal___TREATMENT TW.docx - 12:14 [Now, however, since the introd..]  (33:33)   (Super)
Codes:	[Impact_SS_CBHI: close the disparity] [Impact_SS_CBHI: ensure equal access] 
No memos

Now, however, since the introduction of CBHI, women have equal access to health services, just as men and children get timely treatment when they get sick. Last time, everything was managed by the husband, and they waited for the husband's decision to go to a health facility. Now, if they are a CBHI member, they take their membership card and go to the public health facility without delaying to wait for their husband.

P15: KII PHCU Leader___TREATMENT TW.docx - 15:12 [Yes, it makes a huge differenc..]  (30:30)   (Super)
Codes:	[Impact_SS_CBHI: ensure equal access] [Impact_SS_CBHI: removes financial barriers] 
No memos

Yes, it makes a huge difference. Because by becoming a member of CBHI, even the poor, who have nothing, get health services equally with the rich. It has brought great benefits not only to the poor but also to the rich. In addition, if they need to go to the hospital for treatment, they only need a CBHI and a referral letter to get treatment. Being a member of CBHI therefore makes a huge difference in going to the public health facility.

P19: KII_Head of  health Office_ treatment.docx - 19:31 [Health insurance opened equal ..]  (59:59)   (Super)
Codes:	[Impact_SS_CBHI: ensure equal access] 
No memos

 Health insurance opened equal opportunity for children women and youth to get health services.  Insuransiin fayyaa wal-qixxummaa tajaajilamummaa fayyaa kan mirkaneessedha. 

______________________________________________________________________

Code: Impact_SS_CBHI: health seeking behavior {5-0}

P11: KII _Town HO head_ Treatment.docx - 11:26 [now people come for treatment ..]  (55:55)   (Super)
Codes:	[Impact_SS_CBHI: health seeking behavior] 
No memos

now people come for treatment even for a common cold. Without any fear, he comes. He has an insurance book he come at any time. He can treated here until federal hospital. Today nobody can die due to disease without getting health services. The best try done for him.

P13: KII _ _HC-Head _ Ttreatment.docx - 13:13 [Community goes to health facil..]  (33:33)   (Super)
Codes:	[Impact_SS_CBHI: health seeking behavior] 
No memos

Community goes to health facility for checkup and has good health care seeking practice. One of the reasons why their health care seeking is good is that they receive adequate and better health services. 

P14: KII HEW__2024_.docx - 14:20 [Insurance has a huge impact on..]  (36:36)   (Super)
Codes:	[Challenge_SS_CBHI: false demand] [Impact_SS_CBHI: health seeking behavior] 
No memos

Insurance has a huge impact on health service provision. If we don't use it now, we can't save it for next year. The people are mature now. The people go there and take it from here to the governor. So unless there is enough service there, it will be a big obstacle for us again. 

P19: KII_Head of  health Office_ treatment.docx - 19:30 [. Health seeking behavior of t..]  (59:59)   (Super)
Codes:	[challenge_SS_CBH: high demand] [Impact_SS_CBHI: health seeking behavior] 
No memos

. Health seeking behavior of the community increased after the initiation of health insurance. The community has no worries about medical costs for a year once they paid the premium. But there is a shortage of drugs. To my best understanding as experienced health professional, the shortage of drugs is due to the increment of health seeking/demand, not due to health insurance.

P20: KII_Kebele manager__treatment.docx - 20:23 [especially, those who are CBHI..]  (29:29)   (Super)
Codes:	[Benifits_SS_CBHI: free services] [Impact_SS_CBHI: health seeking behavior] 
No memos

especially, those who are CBHI members do not delay tom seek health service. Even though they [members] may not get all medicines from health center, still they get many services free of charge like card, some medicine, laboratory diagnosis, and bed services, which encourage them to visit health facility whenever they feel illness.  

______________________________________________________________________

Code: Impact_SS_CBHI: health service equity {1-0}

P13: KII _ _HC-Head _ Ttreatment.docx - 13:16 [Children often get sick and ar..]  (35:35)   (Super)
Codes:	[Impact_SS_CBHI: empowered women and chidren] [Impact_SS_CBHI: health service equity] 
No memos

Children often get sick and are taken to the hospital quickly if they are insured, and women are treated quickly if they are insured. Children often get sick and go to the hospital early because they have insurance. So, being CBHI membership promotes women's and children care seeking and access to medical care

______________________________________________________________________

Code: Impact_SS_CBHI: minimize catastrophic expenditure {0-0}
______________________________________________________________________

Code: Impact_SS_CBHI: removes financial barriers {4-0}

P13: KII _ _HC-Head _ Ttreatment.docx - 13:15 [A person who does not member i..]  (33:33)   (Super)
Codes:	[Impact_SS_CBHI: removes financial barriers] 
No memos

A person who does not member insurance may be charged higher costs and may not seek services. Being an insurance member increases the need for services. 

P15: KII PHCU Leader___TREATMENT TW.docx - 15:12 [Yes, it makes a huge differenc..]  (30:30)   (Super)
Codes:	[Impact_SS_CBHI: ensure equal access] [Impact_SS_CBHI: removes financial barriers] 
No memos

Yes, it makes a huge difference. Because by becoming a member of CBHI, even the poor, who have nothing, get health services equally with the rich. It has brought great benefits not only to the poor but also to the rich. In addition, if they need to go to the hospital for treatment, they only need a CBHI and a referral letter to get treatment. Being a member of CBHI therefore makes a huge difference in going to the public health facility.

P18: KII_CBHI focal__ treatment.docx - 18:29 [This helps them to seek health..]  (35:35)   (Super)
Codes:	[Impact_SS_CBHI: empowered women and chidren] [Impact_SS_CBHI: removes financial barriers] 
No memos

This helps them to seek health service at the time when they have no money; especially, women and children benefitted from this program, as they have no power to sell properties for the purpose of treatment. They can easily pick their membership ID and go to health facility without worrying for payments. CBHI liberates children and women from dependence to seek healthcare. 

P19: KII_Head of  health Office_ treatment.docx - 19:24 [Our community knows that CBHI ..]  (49:49)   (Super)
Codes:	[Impact_SS_CBHI: removes financial barriers] [Impact_SS_CBHI: women self reliance] 
No memos

 Our community knows that CBHI help people especially women and children to go to health facilities without worries about payments/medical costs. Previously, women and children had to sell livestock or borrow money to go to health facilities. Nowadays, however, people are saying that health insurance has saved us from such problems. They know very well about the benefits of health insurance.

______________________________________________________________________

Code: Impact_SS_CBHI: self reliance {1-0}

P11: KII _Town HO head_ Treatment.docx - 11:29 [So health insurance breaks the..]  (59:59)   (Super)
Codes:	[Impact_SS_CBHI: self reliance] 
No memos

So health insurance breaks the existing dependent culture. So the importance of health insurance is many to list. Insurance is hugely important. As I mentioned earlier to be a member you have to request rather than enforce it by the agency as it great importance for the family. Health insurance is very vital in avoiding dependency. 

______________________________________________________________________

Code: Impact_SS_CBHI: trends of patient flow {1-0}

P11: KII _Town HO head_ Treatment.docx - 11:27 [The behavioral change in healt..]  (56:56)   (Super)
Codes:	[Impact_SS_CBHI: trends of patient flow] 
No memos

The behavioral change in health facilities is dramatically seen. Previously there 20- 25 thousand were served in our town but now the patient flow is nearly 80- 100 thousand OPD. The income from health insurance is 2.5 -5 million but now health centers can collect money from 10 million and above annually. 

______________________________________________________________________

Code: Impact_SS_CBHI: Unreliable finance {1-0}

P13: KII _ _HC-Head _ Ttreatment.docx - 13:11 [The CBHI has an impact with in..]  (31:31)   (Super)
Codes:	[Impact_SS_CBH: deteriorate quality] [Impact_SS_CBHI: Unreliable finance] 
No memos

The CBHI has an impact with inflation. If the contribution and price of the drug balance each other, it will have a good impact. If the insurance contribution is low, it will have a negative impact. Because if the contribution is small, the price of medicines will be expensive and major procedures can be done in hospitals. In this case the CBHI has negative impact on health service provision.

______________________________________________________________________

Code: Impact_SS_CBHI: women and children in indigent HHs {1-0}

P20: KII_Kebele manager__treatment.docx - 20:25 [With this regard, I would you ..]  (33:33)   (Super)
Codes:	[Impact_SS_CBHI: women and children in indigent HHs] 
No memos

With this regard, I would you say it improved health of children and women somewhat; especially, this CBHI is a huge opportunity for children and women live in indigent households.

______________________________________________________________________

Code: Impact_SS_CBHI: women self reliance {4-0}

P16: KII__HEW_ _ _.docx - 16:16 [Without this community based h..]  (35:35)   (Super)
Codes:	[Impact_SS_CBHI: empowered women and chidren] [Impact_SS_CBHI: women self reliance] 
No memos

Without this community based health insurance, they will have difficulty getting treatment/care again when they need it. If there is no insurance, women and children cannot go back to the clinic more than once or twice. If they don't have resources, they will suffer a lot. But if they have insurance, they can go for treatment whenever they want. Therefore, being a CBHI member promotes women's and children's care seeking and access to medical care.

P18: KII_CBHI focal__ treatment.docx - 18:30 [They do not have to wait other..]  (35:35)   (Super)
Codes:	[Impact_SS_CBHI: women self reliance] 
No memos

They do not have to wait other person's decision to go to health facility to get treatment for their illness; it [CBHI] completely liberate children and women. Women do not expected to call their husband from farm or they do not expected to wait until their husband returned to home to go to health facility for themselves or to take their child to health facility.

P19: KII_Head of  health Office_ treatment.docx - 19:24 [Our community knows that CBHI ..]  (49:49)   (Super)
Codes:	[Impact_SS_CBHI: removes financial barriers] [Impact_SS_CBHI: women self reliance] 
No memos

 Our community knows that CBHI help people especially women and children to go to health facilities without worries about payments/medical costs. Previously, women and children had to sell livestock or borrow money to go to health facilities. Nowadays, however, people are saying that health insurance has saved us from such problems. They know very well about the benefits of health insurance.

P20: KII_Kebele manager__treatment.docx - 20:26 [As you know, it is difficult f..]  (33:33)   (Super)
Codes:	[Impact_SS_CBHI: empowered women] [Impact_SS_CBHI: women self reliance] 
No memos

As you know, it is difficult for poor households to seek health services by payment; especially when it comes to women or children, the burden is not easy. Men, most of the time seek health service by selling something like grain or sheep or by borrowing from friends; however, women cannot do this especially when there is limited resource in the home.

______________________________________________________________________

Code: implementation challenge {2-0}~

P 9: KII_ __Cluster_CBHI Desk KII.docx - 9:14 [According to the plan, when we..]  (38:38)   (Super)
Codes:	[implementation challenge] 
No memos

According to the plan, when we go down there, most of the people will not participate in it. So, it will not be done much with the current situation. But don't leave it because it won't be done, but continue to strengthen this thing in the right way, because the one we started now is a pilot. There may be many problems with pilot. But continue to convince, we need to sit down with commitment and make CBHI on the ground. We need to go down to the ground and filter those people. Its underway in and Deder  in our catchment area.

P 9: KII_ __Cluster_CBHI Desk KII.docx - 9:37 [People's annual income was tak..]  (74:74)   (Super)
Codes:	[implementation challenge] 
No memos

People's annual income was taken into consideration. It was made to work at that level and a lot of resource has been invested on it. But the things we encountered when we got down to the ground, now, as I said before, maybe the program was prepared by dividing people into high, middle and lower levels so that they can contribute according to their ability. But when we come to the top, many people are facing the problem of being able to pay this high price. It can be seasonal or may be the time.

______________________________________________________________________

Code: implementation challenge: budget shortage {1-0}

P 9: KII_ __Cluster_CBHI Desk KII.docx - 9:15 [t's necessary to classify thes..]  (38:38)   (Super)
Codes:	[Attitude SS_CBHI: supportive] [implementation challenge: budget shortage] 
No memos

t's necessary to classify these people before the work is done. Who should on the high, who should on the medium, and who should on the lower. But when I got into the practice after already classified, things were difficult. We don't have money at this stage, so it was a bit of a burden to carry it out. we had a slight problem with it. I think it will be fixed continuously

______________________________________________________________________

Code: implementation challenge: high income family {1-0}~

P 9: KII_ __Cluster_CBHI Desk KII.docx - 9:38 [But when we come to the top, m..]  (74:74)   (Super)
Codes:	[implementation challenge: high income family] 
No memos

 But when we come to the top, many people are facing the problem of being able to pay this high price. It can be seasonal or may be the time. What happened to us, cash crops are now dependent on khat, they have a lot of income. This year their khat market has gone down a lot. It was more than expected. They deserve to be treated with loyalty. One of the reasons why we need to measure is because the cash crop in our area is sold at a high level, because it has fallen because people have not been able take this sliding scale to the ground. Maybe something like that could happen to us, so we should be careful from now on.

______________________________________________________________________

Code: Indigent identification process: {0-0}
______________________________________________________________________

Code: Indigent identification process: not fair and some left out {2-0}

P 5: FGD_Women__treatment.docx - 5:21 [I don't think it is fair and t..]  (142:142)   (Super)
Codes:	[Indigent identification process: not fair and some left out] 
No memos

 I don't think it is fair and transparent. I don't believe that all poor people are identified compared based on some solid criteria and grouped as indigent and payer. Because there are very poor individuals that I know who didn't classified as indigent.

P 5: FGD_Women__treatment.docx - 5:22 [I am among the poor individual..]  (143:143)   (Super)
Codes:	[Indigent identification process: not fair and some left out] [service gap: discrimination of Insurance members] 
No memos

 I am among the poor individuals but I am paying health insurance premium. The health workers are not equally serving those who are member of health insurance and those who are not. The care providers are saying that priority for those who have money (non-member of health insurance). Health workers are despising and disrespecting insured clients.

______________________________________________________________________

Code: indigent selection challenge: {1-0}~

P 8: KII HEW_SCH__TREATMENT_TW.docx - 8:5 [To solve this, sometimes what ..]  (23:23)   (Super)
Codes:	[indigent selection challenge:] 
No memos

To solve this, sometimes what we did was take some from a group with a small number of poor people and add more from a group with many poor people. However, there are still poor people who are not CBHI member. There is another forced abduction of the poor people left over from the kebele quota to the payer. Then they couldn't pay, and they left non-members. There are also a few non-members among those who can afford it.

______________________________________________________________________

Code: indigent selection challenge: Qouta is a problem {1-0}~

P 8: KII HEW_SCH__TREATMENT_TW.docx - 8:4 [As in our village, there is a ..]  (22:22)   (Super)
Codes:	[indigent selection challenge: Qouta is a problem] 
No memos

As in our village, there is a payer who pays independently to become a member. There are also poor people who are members. However, there is a group that does not fall into the poor category and also cannot be a payer. This means that a poor person is a person who has nothing and is based on a daily labor job for his daily life. These community groups that are left hanging in the middle cannot afford to pay for membership or renewal. 

______________________________________________________________________

Code: lack of Awareness: {1-0}

P 2: FGD_Men___TREATMENT.docx - 2:26 [The health extension requested..]  (159:159)   (Super)
Codes:	[lack of Awareness:] 
No memos

The health extension requested every individual going from home to home to bring their photo and renew their insurance on time and utilized but due to personal problems they missed it. We do not have problems with the government and HEW. But personal problems as the proverb says 'wallaalli abbaa, bishaan biratti dheebota' literally 'unawareness makes thirsty in the presence of water'. They paid for insurance but due to personal problems, they did not renew by asking HEW and bringing the required photos.

______________________________________________________________________

Code: Lack of money {1-0}

P 1: _FGD_WOMEN_TREATMENT_TW.docx - 1:11 [Everyone is willing to pay and..]  (103:103)   (Super)
Codes:	[CBHI valued program] [Lack of money] 
No memos

Everyone is willing to pay and become a member. The thing that keeps them from paying voluntarily is the lack of money

______________________________________________________________________

Code: long waiting time {1-0}

P 3: FGD_Men__April 08,2024_.docx - 3:10 [Previously, I was member of he..]  (93:93)   (Super)
Codes:	[Discrulination of insurance members] [long waiting time] [Medicine unavailability in government facility] [PB_CBHI: protect from expensive payment] 
No memos

Previously, I was member of health insurance; however, this year I do not want to be a member, because the service that I could get by this membership is not that much, there is no medicine in the government hospital. Not only medicine; as my friend previously raised, they also not permit X-Ray, and ultrasound services for insurance members. When an insurance member sent to X-Ray or ultrasound room, the individuals work in those room intentionally give extended appointment for the service. Imagine how a person who came from rural area wait three or four days to get these services. They [health workers] give you alternatives; if you can wait for long time, you can get these services free of charge by your insurance service, however, if you want it by the same day the request written, you have to go to the private clinics [mentioned the clinics by name]. If these problems resolved, insurance service is a relief for our community especially our rural community. I have good awareness about insurance service; I have no problem of awareness, I decided to drop out from membership due to inadequate services and mistreatment by health workers for insurance members. Even in my opinion, rural community have good awareness about the benefits of health insurance service. Even I learnt about insurance service from a person who came from rural area to seek health service for his child; fortunately, my child and his child admitted to the same room in one hospital in Addis Ababa, at the time, he was member of health insurance and I was not, and he told me about it in detail. Even he helped me to collect some of expensive medicine for my child using his insurance book. 

______________________________________________________________________

Code: Medicine unavailability {0-0}
______________________________________________________________________

Code: Medicine unavailability in government facility {10-0}

P 3: FGD_Men__April 08,2024_.docx - 3:3 [Insurance program is good to p..]  (89:89)   (Super)
Codes:	[CBHI_SS challenge: high fee for middle group] [Medicine unavailability in government facility] [PB_CBHI: protect from un necessary expense] 
No memos

Insurance program is good to protect our community from unnecessary expenses. However, this year the amount of payment has increased by more than double, which makes it difficult for many people especially poor households cannot pay for it despite they have interest to become member. Another major problem with health insurance, especially exacerbated this year is about unavailability of medicine from pharmacy of government health facilities. People are not getting medicines; they are being obligated to buy from private pharmacy at high cost. Sometimes, they may get from Red Cross pharmacy; however, government must work to improve the availability of medicine to sustain this health insurance

P 3: FGD_Men__April 08,2024_.docx - 3:6 [Our unsolved problem with insu..]  (90:90)   (Super)
Codes:	[Medicine unavailability in government facility] 
No memos

Our unsolved problem with insurance service is the problem with medicine unavailability; there is no medicine in the government hospital, and they send you to outside to private pharmacy or sometimes to Red Cross pharmacy. Some cheap medicines are available in Red Cross pharmacy; however, they [Red Cross] never give you expensive medicine despite you are member of insurance.

P 3: FGD_Men__April 08,2024_.docx - 3:8 [why our community loose intere..]  (92:92)   (Super)
Codes:	[Medicine unavailability in government facility] [Un availability of Laboratory and diagnostic service] 
No memos

why our community loose interest from becoming insurance service is unavailability of medicine and laboratory services inside government hospital. I am very sad when thinking about rural community; imagine they do not seek care until their illness become worse, and when decide to seek care, they are totally depend on their insurance ID card and do not prepare any money for treatment purpose; they only prepare transport expenses. The reality in the health facility is quite different; they may not get medicine, they may sent to outside for laboratory or X-Ray, after which they start begging money from strangers and cry sitting in the hospital. They may have no preparation for unavailable service; all they have for treatment is their insurance membership card. This is why they disappoint, frustrate and cry when they are sent to outside and hear the price of X-Ray or medicine at private pharmacy or clinic

P 3: FGD_Men__April 08,2024_.docx - 3:10 [Previously, I was member of he..]  (93:93)   (Super)
Codes:	[Discrulination of insurance members] [long waiting time] [Medicine unavailability in government facility] [PB_CBHI: protect from expensive payment] 
No memos

Previously, I was member of health insurance; however, this year I do not want to be a member, because the service that I could get by this membership is not that much, there is no medicine in the government hospital. Not only medicine; as my friend previously raised, they also not permit X-Ray, and ultrasound services for insurance members. When an insurance member sent to X-Ray or ultrasound room, the individuals work in those room intentionally give extended appointment for the service. Imagine how a person who came from rural area wait three or four days to get these services. They [health workers] give you alternatives; if you can wait for long time, you can get these services free of charge by your insurance service, however, if you want it by the same day the request written, you have to go to the private clinics [mentioned the clinics by name]. If these problems resolved, insurance service is a relief for our community especially our rural community. I have good awareness about insurance service; I have no problem of awareness, I decided to drop out from membership due to inadequate services and mistreatment by health workers for insurance members. Even in my opinion, rural community have good awareness about the benefits of health insurance service. Even I learnt about insurance service from a person who came from rural area to seek health service for his child; fortunately, my child and his child admitted to the same room in one hospital in Addis Ababa, at the time, he was member of health insurance and I was not, and he told me about it in detail. Even he helped me to collect some of expensive medicine for my child using his insurance book. 

P 3: FGD_Men__April 08,2024_.docx - 3:11 [the problems like unavailabili..]  (95:95)   (Super)
Codes:	[Medicine unavailability in government facility] [Un availability of Laboratory and diagnostic service] 
No memos

the problems like unavailability of medicine and X-Ray or ultrasound service are reasons for me to decide not to renew my membership. Additionally, the payment for insurance membership increase from time to time; why I pay this much money when I cannot get medicines from hospital? I also have no health problem and I do not need insurance that much; last year, I was member but I did not seek health care even one day. Insurance is good for those who have diseases like hypertension and diabetes mellitus. For instance, my mother is hypertensive and getting advantage from insurance service as her illness need lifelong treatment.

P 3: FGD_Men__April 08,2024_.docx - 3:15 [While individuals with suffici..]  (97:97)   (Super)
Codes:	[Medicine unavailability in government facility] 
No memos

While individuals with sufficient property can easily afford insurance payments, those who are economically poor face significant challenges. It is crucial for the government to pay attention to poor segment of our community when making decisions, particularly concerning financial obligations such as insurance payments. What I want to tell you again is that the availability of medicines need to be improved. The main factor disappoint our community and push the community from insurance service.

P 3: FGD_Men__April 08,2024_.docx - 3:16 [The experiences shared by my f..]  (98:98)   (Super)
Codes:	[Medicine unavailability in government facility] [Service gap: Poor reception of health workers: it is painful] 
No memos

The experiences shared by my friends are all true! What we can fully get from hospital free of charge using insurance service is only bed service. Otherwise, we all buy medicine from private pharmacy, especially when they see insurance membership card with us, they automatically order us to buy the medicine from private pharmacy. We paid 1300 to 1900 birr for the insurance service, why they mistreat us and deny us the medicines? Do government health facilities have management? Is there a responsible body who checks where the medicines purchased for us go? Rich individuals have alternatives; they can buy the medicine from private pharmacy or even they can afford to seek health service from private clinic. We, the poor individuals have however do not have alternative, we have no choice than going to government health facility. It is painful when one poor person paid 1300 birr for insurance service and denied medicine from government hospital.

P 4: FGD_MEN_ treatment.docx - 4:12 [Sometimes there was a problem ..]  (126:126)   (Super)
Codes:	[Medicine unavailability in government facility] [Poor reception of health workers:] 
No memos

 Sometimes there was a problem of good reception by the professionals. People are concerned that there may be a shortage of drugs supplies. Some people may have no money and concerned the cash contribution required by the community based health insurance member. 

P 4: FGD_MEN_ treatment.docx - 4:13 [The community will pay but the..]  (128:128)   (Super)
Codes:	[Medicine unavailability in government facility] 
No memos

 The community will pay but there was a shortage of medicines and that is a community concern.

P 4: FGD_MEN_ treatment.docx - 4:25 [We were complaining that the s..]  (150:150)   (Super)
Codes:	[Medicine unavailability in government facility] [Un availability of Laboratory and diagnostic service] 
No memos

We were complaining that the supply of medicines was a problem. There wasn't even a laboratory service but now it has improved. Before that, the community had complained about the lack of laboratory services and medicine supply. It is good to improve these services.

______________________________________________________________________

Code: Medicine unavailability in government facility: {1-0}

P 3: FGD_Men__April 08,2024_.docx - 3:26 [There is no doubt regarding th..]  (111:111)   (Super)
Codes:	[Medicine unavailability in government facility:] [PB_CBHI: increased health seeking behavior by community] [Un availability of Laboratory and diagnostic service] 
No memos

There is no doubt regarding the improvement of health seeking practices, especially for women and our children; so many changes are there, now everyone can go to health facility without worrying about payment. The problem is that you cannot get enough medicine, and other services like X-Ray, and ultrasound. Even you cannot get specialist doctors; they sit in their own clinic, they are not available in government hospital even for an hour. If you want to discuss about your health with specialist, you have to go to their private clinic. They collect up to 5000 birr from single person. Visiting health facility has no value if you cannot get medicine for your disease. Another problem is that they never reimburse your expenses that you paid to buy medicine from private clinic or they amount you paid for X-Ray service at private clinic. They informed us that the government ordered them not to reimburse any expenses except for the receipt provided from Red Cross pharmacy.

______________________________________________________________________

Code: Medicine unavailability in government facility: may be fraud {1-0}

P 3: FGD_Men__April 08,2024_.docx - 3:13 [How one medicine is available ..]  (96:96)   (Super)
Codes:	[Medicine unavailability in government facility: may be fraud] 
No memos

 How one medicine is available in private pharmacy and not available in government hospital? In my opinion, those medicine which are not available in public health facilities and readily available from private pharmacies are stolen from public health facilities. This time, it is difficult to praise insurance service, it is full of problem and people do not want to stay as a mem

______________________________________________________________________

Code: members increasing {2-0}

P 9: KII_ __Cluster_CBHI Desk KII.docx - 9:16 [Now it is reaching seventy nin..]  (20:20)   (Super)
Codes:	[members increasing] 
No memos

 Now it is reaching seventy nine percent. This shows us the progress that shows us the progress that the society's understanding and becoming more prominent and it is becoming more and more aware of the situation.

P 9: KII_ __Cluster_CBHI Desk KII.docx - 9:21 [it has been improving from tim..]  (48:48)   (Super)
Codes:	[members increasing] 
No memos

it has been improving from time to time, especially in two thousand sixteen, it was better in the community than in two thousand fifteen. There are some people come for treatment without taking out CBHI IDs and don't use them. The most important thing about CBHI is the situation in which it is being used and served, still now.

______________________________________________________________________

Code: MEMBERSHIP GAP: challenging for new couples {2-0}

P 6: FGdD_Female_ treatment.docx - 6:14 [a gap has been seen among the ..]  (136:136)   (Super)
Codes:	[MEMBERSHIP GAP: challenging for new couples] 
No memos

 a gap has been seen among the new householders. Those recently separated from their parent due to marriage and new to health insurance. So they are not engaged in health insurance and are treated by their money.  As health insurance has its own deadline for new taking or renewing, if they do not come with a time frame they cannot get while getting diseases.

P 6: FGdD_Female_ treatment.docx - 6:17 [Sometimes newly married couple..]  (139:139)   (Super)
Codes:	[MEMBERSHIP GAP: challenging for new couples] 
No memos

Sometimes newly married couples may not think or forget to engage in insurance because sometimes they think as they received by their parents

______________________________________________________________________

Code: MEMBERSHIP GAP: Forced membership {1-0}

P 7: FGDs_Men___ treatment.docx - 7:29 [The people go and come back cr..]  (170:170)   (Super)
Codes:	[MEMBERSHIP GAP: Forced membership] [Service gap: Medicine unavailability] 
No memos

The people go and come back crying. You were taking insurance forcing us with force. You were telling us to go to health facility and this health insurance is profitable for you. When we go to health centers, we don't get any service. They give us only tablet, they don't give us a single injection. They gave us antipain pills and told us to go

______________________________________________________________________

Code: MEMBERSHIP GAP: poor left out {0-0}
______________________________________________________________________

Code: MEMBERSHIP GAP: some poor left out {0-0}
______________________________________________________________________

Code: membership is mandatory {0-0}
______________________________________________________________________

Code: Membership: majority of the community are members {2-0}

P 6: FGdD_Female_ treatment.docx - 6:7 [even though, we cannot say 100..]  (133:133)   (Super)
Codes:	[Membership: majority of the community are members] 
No memos

even though, we cannot say 100% are members of health insurance, the majority of individuals are members of health insurance. In rare cases may some be missed but most of the individuals are utilized. This missed is not the result of unknowing of the advantages of insurance but rather resulted from the incompleteness of membership like not timely bringing the photos' on time. For example, if one family has 10 members it needs 10 individual photos made which to delay in benefiting from insurance. 

P 6: FGdD_Female_ treatment.docx - 6:9 [Except for those who resisted ..]  (134:134)   (Super)
Codes:	[Membership: majority of the community are members] 
No memos

 Except for those who resisted participating in the meeting, most of the residents were educated, learning from neighbors of experiences by health extension and some were repeatedly informed and then became a membership. 

______________________________________________________________________

Code: Newborn challenge: Cannot use the service until the next tound {1-0}

P 5: FGD_Women__treatment.docx - 5:20 [a newborn baby who born after ..]  (138:138)   (Super)
Codes:	[Denial of service : the new kids or family photo is not on the card] [Newborn challenge: Cannot use the service until the next tound] 
No memos

a newborn baby who born after the premium collection can't be member of health insurance until the next round of premium collection schedule. This prone newborns to death. It should be allowed for newborns to be member of health insurance at any time regardless of the fixed premium collection period. Thus, such issue need to be modified.

______________________________________________________________________

Code: No discrimination between cbhi members and non CBHI member {3-0}

P 1: _FGD_WOMEN_TREATMENT_TW.docx - 1:16 [In my opinion, it is the chara..]  (108:108)   (Super)
Codes:	[No discrimination between cbhi members and non CBHI member] 
No memos

 In my opinion, it is the characteristics of the health professionals that are not homogeneous, but the CBHI is always useful. This means that some welcome and treat, while others leave even when someone is sick and suffering. So our complaint is against the health professionals. As in this kebele, there is no discrimination between CBHI members and non-CBHI members in reception or service delivery. Another concern is the lack of medicines in the health center.

P 6: FGdD_Female_ treatment.docx - 6:41 [To tell the truth all are equa..]  (180:180)   (Super)
Codes:	[No discrimination between cbhi members and non CBHI member] 
No memos

To tell the truth all are equally treated regardless of being a member of insurance. I did not find discrimination between insurance users and non-users.  The difference was only paying out of pocket or not.

P 6: FGdD_Female_ treatment.docx - 6:42 [As P1 said I have no different..]  (181:181)   (Super)
Codes:	[No discrimination between cbhi members and non CBHI member] 
No memos

As P1 said I have no different idea., we have registered for medical records based on our queue and also wait for examinations based on our queue unless emergency cases come in between. They served us equally. 


______________________________________________________________________

Code: no one can be fully confident in insurance without having money in his pocket because finally he will sent to private {1-0}

P 2: FGD_Men___TREATMENT.docx - 2:32 [Regarding health-seeking behav..]  (173:173)   (Super)
Codes:	[Health seeking behavior: poor for two reasons:] [no one can be fully confident in insurance without having money in his pocket because finally he will sent to private] 
No memos

 Regarding health-seeking behavior, there is a difference between the previous trend and the current. The difference, in the previous people took traditional drugs. For example, if someone is ill, He may drink called the so-called 'Bokkolluu'. In the past, if someone drank this Bokkollu cured immediatily. But now if someone drinks it he cannot cured because everything/climate has changed. But it is not working now. For example, my father let him rest in peace, when sick he drank two cups of Bokkollu and then cured immediately and took a bath. Some who were exposed to pneumonia (dhukkuba michii) drank it and saw results immediately. But now if you drink several cups, no improvement due to climate change rather than looking for a health facility. For this reason, every individual must look for health facilities because if he drinks the bokkolluu no change. He cures when he goes to a health facility. b/c of this they go for health facility. However, their health-seeking behavior is not immediate after ill. They stayed from two to three days before health seeking. The reasons are related to two things. First,  Perception toward health facilities. He says 'If I go for  healthcare, they provide me drugs simply by asking about my problems orally without further investigation and result in not addressing my disease.' So he delayed to health seek as he assumed no value in going there. The second for the delay is the perception that even if  I go to a public health facility using health insurance I can't get anything and back to the private clinic so He may be delayed in for searching money used to be treated at a private clinic. So he delayed the until he got money for treatment. I tell you the truth if someone is sick,  no one can be fully confident in insurance without having money in his pocket because finally he will sent to private.

______________________________________________________________________

Code: No Willingness to join again: {0-0}~
______________________________________________________________________

Code: Non members suffering {1-0}~

P 7: FGDs_Men___ treatment.docx - 7:32 [The body that is not a member ..]  (174:174)   (Super)
Codes:	[Non members suffering] 
No memos

The body that is not a member here is in a serious trouble. A private clinic is asking for anything as they want. He may be affected by his economic resources and health; he does not get prompt services. The non-member is at a disadvantage, at a great disadvantage.

______________________________________________________________________

Code: OOP expense: still big problem {1-0}

P 3: FGD_Men__April 08,2024_.docx - 3:9 [In some cases, they may find t..]  (92:92)   (Super)
Codes:	[OOP expense: still big problem] 
No memos

In some cases, they may find themselves compelled to borrow money from relatives without any prior preparation, leading them to unexpectedly sell their cattle. I, myself lend money for my three relatives who came from rural area to seek treatment and faced the same problem.

______________________________________________________________________

Code: Opportunity_SSCBHI: Community understanding of CBHI {1-0}

P19: KII_Head of  health Office_ treatment.docx - 19:22 [The community is well aware th..]  (49:49)   (Super)
Codes:	[Opportunity_SSCBHI: Community understanding of CBHI] 
No memos

The community is well aware that health insurance is the health guarantee of their families which is literally means “Hawaasni insuransiin fayyaa wabii fayyaa maatii isaanii ta'uu sirritti beeku”.

______________________________________________________________________

Code: Over expectation {1-0}~

P 1: _FGD_WOMEN_TREATMENT_TW.docx - 1:9 [People like me who have seen t..]  (99:99)   (Super)
Codes:	[Over expectation] [PB_ss_CBHI: good package of service] [PB_SS_CBHI: tax based] [Poor reception of health workers:] 
No memos

People like me who have seen the benefits at various times will not hesitate to become members, even if they lose one day's service. However, clients, especially those who come for emergencies and lose services, may become frustrated and withdraw from membership. Some clients think that they should never lose service after paying for CBHI due to a lack of understanding or advice and are disappointed when they lose a single service. Additionally, those who have repeatedly lost treatment and are disappointed with the reception of professionals may also leave the membership. In our kebele, to prevent the loss of members, HEWs provide advice, and there is a compulsory payment of CBHI with taxes.

______________________________________________________________________

Code: payment method {1-0}

P 9: KII_ __Cluster_CBHI Desk KII.docx - 9:33 [Now, even more so, if health f..]  (62:62)   (Super)
Codes:	[payment method] 
No memos

Now, even more so, if health facilities are a member of CBHI all those people's money will be added to the health facilities there. It will deposit at once not turn by turn. We will pay them every quarter. Therefore, if the money that comes is not wasted, if it is directed to what they want, what they need to buy, and what they have, it has a great contribution to ensure that the quality of the service is maintained. But it needs a plan, and proper management.

______________________________________________________________________

Code: PB: research may address our complain to government {1-0}~

P 2: FGD_Men___TREATMENT.docx - 2:18 [I was treated and cured at a p..]  (140:141)   (Super)
Codes:	[PB: research may address our complain to government] 
No memos

I was treated and cured at a private clinic. So a private clinic is better treat me.” said. For example, if you go to interview every individual those not a member of this insurance they reply for you this answer.  It is not only lack of money that prevents one from being enrolled in health insurance but those problems are reasons for not being members. Those are the complaints. 
As already discussed,  the community raised 100% of their problems but when it was not forwarded for the woreda, I don't think 50% of the community complaints were forwarded. Only 0% percent forwarded for higher-level leaders. It was never presented to the government. If it is presented to the government, I hope the government can handle it.  It was never presented to the government. So I am very happy for such kind of research conducted here. Most of the time we hear such types of studies from the media. But not been done here before. 

______________________________________________________________________

Code: PB_CBHI:  guarantee ffor community and facilities {0-0}
______________________________________________________________________

Code: PB_CBHI:  inclussion of all family memebers for the service {2-0}

P 6: FGdD_Female_ treatment.docx - 6:1 [In the beginning, we heard tha..]  (125:125)   (Super)
Codes:	[Attitude SS_CBHI: Supportive and recomend] [PB_CBHI:  inclussion of all family memebers for the service] [PB_CBHI: No out of pocket payment] [Premium contribution: fair: equivalent with  one time service at private] 
No memos

In the beginning,  we heard that health insurance be served to get health care without payment. They told us once we paid it would be servi for ourselves and our family. we paid for it and we received receipts based on our payment. And that individual who cannot able to pay can get a free membership. There are three different categories of payment schemes. So the health insurance is very important. The amount we pay for a membership that is served for a year for us and our children may be equivalent to the payment we pay at one visit of service at a private clinic.

P 7: FGDs_Men___ treatment.docx - 7:9 [I looked at it again and now w..]  (124:124)   (Super)
Codes:	[PB_CBHI:  inclussion of all family memebers for the service] 
No memos

I looked at it again and now we have seen the results. It has beautiful results and we are using it very well. It will serve the family if you have it, and if you come again and again. It's a beautiful thing.

______________________________________________________________________

Code: PB_CBHI:  insurance  is the strength of the poor {1-0}~

P 5: FGD_Women__treatment.docx - 5:3 [Insuransiin fayyaa humna rakka..]  (114:114)   (Super)
Codes:	[PB_CBHI:  insurance  is the strength of the poor] 
No memos

Insuransiin fayyaa humna rakkataa qarshii isaan yaalamuu hin dandenyeti. The problem is regarding health services. Health workers are not willing to serve insurance members. The attitude of health worker for clients are very poor. Most of the time, it takes days or weeks to contact doctors. People are vulnerable to unnecessary costs such as cost of transportation to contact health care providers at public health facilities. Due to this, those who can able to pay go to private health facilities. But if you have no money, you have no option that begging to get services from public health facilities. In general, health insurance has a number of benefits including referral services up to Addis Ababa black lion. But the health services provided at public health institutions need to be improve.

______________________________________________________________________

Code: PB_CBHI:  referal service {4-0}

P 5: FGD_Women__treatment.docx - 5:4 [Health workers are not willing..]  (114:114)   (Super)
Codes:	[PB_CBHI:  referal service] [Service gap: Poor attitude of health workers toward insurance members] 
No memos

Health workers are not willing to serve insurance members. The attitude of health worker for clients are very poor. Most of the time, it takes days or weeks to contact doctors. People are vulnerable to unnecessary costs such as cost of transportation to contact health care providers at public health facilities. Due to this, those who can able to pay go to private health facilities. But if you have no money, you have no option that begging to get services from public health facilities. In general, health insurance has a number of benefits including referral services up to Addis Ababa black lion. But the health services provided at public health institutions need to be improve. 

P 6: FGdD_Female_ treatment.docx - 6:5 [We cannot finshed the insuranc..]  (127:127)   (Super)
Codes:	[Attitude toward CBHI: good and eager to renew] [PB_CBHI:  referal service] [PB_CBHI: good pakage of service (admission)] [PB_CBHI: No out of pocket expenditure] 
No memos

We cannot finshed the insurance benefit by listing it within this limited time. At the start of insurance in the nation, most people fear to engage in insurance by saying our money would treat us. We do not want this membership card and call it a 'carton'.we can treat ourselves with no need from the government. However, after the community understood the advantages of health insurance, they immediately paid the annual premium and renewed the card within a given deadline and utilizing. Like fertilizer in its beginning most people take it forcibly we also had taken insurance. But now that we the farmers searching for health insurance like sugar because they are well aware of the importance of health insurance. We have been treated here but if it is beyond the health center we are referred to Dodola Hospital. During this referral service, we only paid for transportation and there is no anything we paid for the Dodola hospital. We never pay for admission, cards, and drugs. So the importance of health insurance is so many to list.  So we would like to thank the government for providing such services for us.

P 6: FGdD_Female_ treatment.docx - 6:15 [. It is very important for the..]  (139:139)   (Super)
Codes:	[PB_CBHI:  referal service] [PB_ss_CBHI: good package of service] 
No memos

. It is very important for the laboring mother, to perform surgery through referral of a patient to another far health facility. So it has many benefits. B

P 7: FGDs_Men___ treatment.docx - 7:3 [We have a pretty good experien..]  (110:110)   (Super)
Codes:	[PB_CBHI:  referal service] [PB_CBHI: better health service] [Renewal: yes] 
No memos

 We have a pretty good experience in health insurance. As ourselves and as a community of this , whether the lower, higher or middle class, we are benefited from the huge profits. We have received beautiful services in a short time. Different people who went the referrals have gained great experience by becoming a member of this insurance. We will continue in the future it is very good for us, very profitable for us. This is what I have.

______________________________________________________________________

Code: PB_CBHI: adequate service {0-0}
______________________________________________________________________

Code: PB_CBHI: adequate service with respect {0-0}
______________________________________________________________________

Code: PB_CBHI: better access to medicine and laboratory {1-0}

P 4: FGD_MEN_ treatment.docx - 4:23 [People who are members of comm..]  (148:148)   (Super)
Codes:	[PB_CBHI: better access to medicine and laboratory] 
No memos

People who are members of community based health insurance are also getting better access to services such as medicines, laboratories and professional advice.

______________________________________________________________________

Code: PB_CBHI: better child treatment {1-0}

P 1: _FGD_WOMEN_TREATMENT_TW.docx - 1:36 [CBHI revolutionized our childr..]  (137:137)   (Super)
Codes:	[PB_CBHI: better child treatment] [PB_CBHI: early treatment seeking] 
No memos

CBHI revolutionized our children's treatment. Because in the past, if a child got sick, we would go to the health center after the children were sick for a long time because of lack of money. Now that CBHI has arrived, we go for treatment on time when our child is sick. In general, a person without CBHI is usually treated delayed, while a person with CBHI is treated early

______________________________________________________________________

Code: PB_CBHI: better health {1-0}

P 4: FGD_MEN_ treatment.docx - 4:8 [we are getting our health. Bef..]  (117:117)   (Super)
Codes:	[PB_CBHI: better health] [PB_CBHI: No selling cattle for health expense] 
No memos

we are getting our health. Before this insurance started, we used to sell our cattle and get treatment, but now there is insurance and we are using it properly.

______________________________________________________________________

Code: PB_CBHI: better health service {3-0}

P 7: FGDs_Men___ treatment.docx - 7:3 [We have a pretty good experien..]  (110:110)   (Super)
Codes:	[PB_CBHI:  referal service] [PB_CBHI: better health service] [Renewal: yes] 
No memos

 We have a pretty good experience in health insurance. As ourselves and as a community of this , whether the lower, higher or middle class, we are benefited from the huge profits. We have received beautiful services in a short time. Different people who went the referrals have gained great experience by becoming a member of this insurance. We will continue in the future it is very good for us, very profitable for us. This is what I have.

P 7: FGDs_Men___ treatment.docx - 7:11 [People have nothing to afraid ..]  (128:128)   (Super)
Codes:	[PB_CBHI: better health service] 
No memos

 People have nothing to afraid if they have understanding. We have not had this understanding of before and we are not using it a lot. We pay for it and just put it down. One person from my village was sick and referred from here. They haven't taken anything from us starting from bed and blood. I've seen this results for myself. I even told to other not to take this easy. It is not easy. I told my neighbors to go and get it

P 7: FGDs_Men___ treatment.docx - 7:27 [People learned from each other..]  (162:162)   (Super)
Codes:	[PB_CBHI: better health service] 
No memos

 People learned from each other and new members are joining. Anyone who has seen this service and looked at this will not even miss it. I say with great pleasure.

______________________________________________________________________

Code: PB_CBHI: better service for women {1-0}

P 6: FGdD_Female_ treatment.docx - 6:16 [It is very important for the l..]  (139:139)   (Super)
Codes:	[PB_CBHI: better service for women] 
No memos

It is very important for the laboring mother, to perform surgery through referral of a patient to another far health facility. So it has many benefits. 

______________________________________________________________________

Code: PB_CBHI: better service for women and children {3-0}

P 4: FGD_MEN_ treatment.docx - 4:27 [Women and children need health..]  (153:153)   (Super)
Codes:	[PB_CBHI: better service for women and children] 
No memos

Women and children need health services and if they have insurance, they get the necessary services at any time. Insurance is properly useful especially for pregnant women even during childbirth and also very useful for sick children. So it means it will help women and children get the treatment they need when they need it. Health insurance has good benefits for pregnant women and children.

P 4: FGD_MEN_ treatment.docx - 4:28 [Before insurance was introduce..]  (154:154)   (Super)
Codes:	[PB_CBHI: better service for women and children] 
No memos

Before insurance was introduced, our women were dying in childbirth. Now, however, there is insurance and they are saving women's lives because they are being taken to the hospital/health center by ambulance on time and receiving adequate treatment.

P 6: FGdD_Female_ treatment.docx - 6:43 [n the case of a newborn and th..]  (184:184)   (Super)
Codes:	[PB_CBHI: better service for women and children] 
No memos

n the case of a newborn and the case of the custodian, we have to notify the agency and get service by insurance so we do not have any problems with children and women in utilizing. 

______________________________________________________________________

Code: PB_CBHI: covering indigient fee by government {1-0}

P 2: FGD_Men___TREATMENT.docx - 2:11 [In the case of the benefit of ..]  (127:127)   (Super)
Codes:	[PB_CBHI: covering indigient fee by government] 
No memos

In the case of the benefit of health insurance, for example, if someone has no capacity to pay the insurance premium, the government will buy it for him. Then he enrolled in health insurance with us which is a good job. Due to the support of Ganda, he benefited by getting health services and was saved from dying due to lack of health services. I thank Ganda for their support and good work. Now everybody understands the benefits of insurance and ambulance service and engaging in health insurance

______________________________________________________________________

Code: PB_CBHI: developed passion {1-0}

P 1: _FGD_WOMEN_TREATMENT_TW.docx - 1:7 [his means that anyone who has ..]  (97:97)   (Super)
Codes:	[CBHI _challenge: lack of understanding] [CBHI_challenge: not only professionals] [PB_CBHI: developed passion] [PB_CBHI: eager to renew] [Perceived benefits of CBHI: free service] 
No memos

his means that anyone who has seen and heard of its benefits is eager to renew their membership. For example, in the last year, a child of a relative of mine who is a member of CBHI was lying in bed for surgery at Tulu Bolo. The child was treated free of all service fees, including food. In the same ward, there was a man without CBHI being treated, and he was treated and discharged at great expense for similar services as that of a child of my relative. Since then, I have developed a passion for the CBHI. I also told people I knew about the benefits and made them interested. So in my opinion, it is a lack of understanding, but the problem of professionals alone does not prevent renewing the membership.

______________________________________________________________________

Code: PB_CBHI: eager to renew {1-0}

P 1: _FGD_WOMEN_TREATMENT_TW.docx - 1:7 [his means that anyone who has ..]  (97:97)   (Super)
Codes:	[CBHI _challenge: lack of understanding] [CBHI_challenge: not only professionals] [PB_CBHI: developed passion] [PB_CBHI: eager to renew] [Perceived benefits of CBHI: free service] 
No memos

his means that anyone who has seen and heard of its benefits is eager to renew their membership. For example, in the last year, a child of a relative of mine who is a member of CBHI was lying in bed for surgery at Tulu Bolo. The child was treated free of all service fees, including food. In the same ward, there was a man without CBHI being treated, and he was treated and discharged at great expense for similar services as that of a child of my relative. Since then, I have developed a passion for the CBHI. I also told people I knew about the benefits and made them interested. So in my opinion, it is a lack of understanding, but the problem of professionals alone does not prevent renewing the membership.

______________________________________________________________________

Code: PB_CBHI: eager to renew membership {1-0}

P 1: _FGD_WOMEN_TREATMENT_TW.docx - 1:38 [We have a family and we want t..]  (140:140)   (Super)
Codes:	[PB_CBHI: eager to renew membership] 
No memos

We have a family and we want to continue this CBHI to renew our membership and use.

______________________________________________________________________

Code: PB_CBHI: ealy health seeking compared to not members {1-0}

P 2: FGD_Men___TREATMENT.docx - 2:33 [if a person that has insurance..]  (174:174)   (Super)
Codes:	[PB_CBHI: ealy health seeking compared to not members] 
No memos

 if a person that has insurance will go for health care regardless of the amount of money in his hand but those are not members of the insurance may take time to search for money. He may delay for 5-6 days by assuming it may improve by itself. So there is a little bit of difference between those insurance and noninsurance. However, the person with insurance does not have full confidence in the health facility. He did not get all the drugs from health facility. He worries about getting it. because heard from he has experience in not getting drugs from his neighbor in the 2-3 days. The main problems and the main investigation have to be done in this area. This is my idea. 

______________________________________________________________________

Code: PB_CBHI: early health seeking compared to not members {1-0}

P 6: FGdD_Female_ treatment.docx - 6:38 [I think those who do not have ..]  (174:174)   (Super)
Codes:	[PB_CBHI: early health seeking compared to not members] 
No memos

I think those who do not have insurance may delayed until they search for money for the treatment. So those who have insurance cards better practice health care.

______________________________________________________________________

Code: PB_CBHI: early treatment seeking {1-0}

P 1: _FGD_WOMEN_TREATMENT_TW.docx - 1:36 [CBHI revolutionized our childr..]  (137:137)   (Super)
Codes:	[PB_CBHI: better child treatment] [PB_CBHI: early treatment seeking] 
No memos

CBHI revolutionized our children's treatment. Because in the past, if a child got sick, we would go to the health center after the children were sick for a long time because of lack of money. Now that CBHI has arrived, we go for treatment on time when our child is sick. In general, a person without CBHI is usually treated delayed, while a person with CBHI is treated early

______________________________________________________________________

Code: PB_CBHI: eraly health seeking compared to not members {1-0}

P 5: FGD_Women__treatment.docx - 5:29 [Those who have health insuranc..]  (156:156)   (Super)
Codes:	[PB_CBHI: eraly health seeking compared to not members] [Premium contribution: better if  breakdown to monthly contributed] 
No memos

Those who have health insurance run to health facilities when get sick even if they have no money at their hand.  Individuals who do not visit health facility for their sickness are either those who do not have money from bank or not member of health insurance. Such people ride at home and wait day of their death. Please take our recommendations to those who are working on health insurance, we need to save our money monthly or every some months by opening our account at their office or somewhere they prefer  to contribute health insurance premium.

______________________________________________________________________

Code: PB_CBHI: free health service {5-0}

P 2: FGD_Men___TREATMENT.docx - 2:1 [we see its benefits and proces..]  (109:109)   (Super)
Codes:	[PB_CBHI: free health service] 
No memos

we see its benefits and processes, once the insurance payment takes place, it will be serviced for one year. All family members listed and their photos attached on the health insurance card have served free when they got a disease. For one year they can repeatedly get health service without any additional payment. 

P 2: FGD_Men___TREATMENT.docx - 2:2 [Being a member of health insur..]  (111:112)   (Super)
Codes:	[PB_CBHI: free health service] 
No memos

Being a member of health insurance in advance helps me get treated without spending money when I get sick and back to my health and family.
 So its objectives and process are very good.  I understand this and accept it. I started using insurance when it was customized to our Kebele/ganda by paying the required amount from me and then they took my photo and my family photos, since then I utilizing it.

P 4: FGD_MEN_ treatment.docx - 4:5 [f you go to a private clinic, ..]  (113:113)   (Super)
Codes:	[PB_CBHI: free health service] 
No memos

f you go to a private clinic, it will cost more, but if you have insurance, you will be treated for free. So everyone is a member.

P 4: FGD_MEN_ treatment.docx - 4:24 [The insured person is treated ..]  (149:149)   (Super)
Codes:	[PB_CBHI: free health service] 
No memos

The insured person is treated free of charge and receives essential services such as medicine, laboratory and even professional advice. But people who are not insurance members will have difficulty accessing these services unless they have enough money

P 4: FGD_MEN_ treatment.docx - 4:26 [think a person without insuran..]  (151:151)   (Super)
Codes:	[PB_CBHI: free health service] 
No memos

 think a person without insurance will have difficulty getting services from other hospitals if he/she needs those services. If you have insurance, however, you get free services up to the biggest hospitals.

______________________________________________________________________

Code: PB_CBHI: Free service for poor {3-0}

P 3: FGD_Men__April 08,2024_.docx - 3:5 [As my friends said, insurance ..]  (90:90)   (Super)
Codes:	[CBHI_SS challenge: the fee for insurance is low compared to the expected service] [PB_CBHI: Free service for poor] 
No memos

As my friends said, insurance service benefitted us; especially poor individuals who cannot immediately go to health facility at the time of illness by their own payment. You can get so many services free of charge once you become member of health insurance; you will not pay for bed service, you will not pay for laboratory services, and medicines if available inside that health facility. In my opinion, the payment for health insurance membership is very low when compare it with the advantages we are getting free of charge.

P 4: FGD_MEN_ treatment.docx - 4:34 [I am poor and the government c..]  (162:162)   (Super)
Codes:	[PB_CBHI: Free service for poor] 
No memos

I am poor and the government covered my insurance payments. so I say that it will continue for the poor like me in the future and also for me to continue the insurance service.

P 5: FGD_Women__treatment.docx - 5:1 [Health insurance is very impor..]  (111:111)   (Super)
Codes:	[PB_CBHI: Free service for poor] [Service gap: Un availability of Laboratory and diagnostic service] 
No memos

Health insurance is very important. It benefited us well. For instance, there are very poor people who can't able to visit health facilities during their sickness for the lack of money especially during this inflation period. Thus, such kind of people highly benefited from health insurance. But the problem is that health workers do not do deep investigation for insured people. For instance, the do not do laboratory investigation for insured people. They ask oral history and prescribe medications. This was done to save the laboratory supplies and to avoid its shortage

______________________________________________________________________

Code: PB_CBHI: Free service for poor and diasable {1-0}

P 7: FGDs_Men___ treatment.docx - 7:4 [Health insurance does not take..]  (112:112)   (Super)
Codes:	[PB_CBHI: Free service for poor and diasable] 
No memos

Health insurance does not take any money from us, as disabled person. We take the advantage of being included in those who couldn't afford to pay.  We didn't pay any money as unemployed and the disabled person in this , but we are using health insurance well. This thing is good, I'm very happy

______________________________________________________________________

Code: PB_CBHI: Free service for the  poor {1-0}

P 5: FGD_Women__treatment.docx - 5:2 [Health insurance has a great a..]  (112:112)   (Super)
Codes:	[PB_CBHI: Free service for the  poor] [service gap: discrimination of Insurance members] [Service gap: Medicine unavailability] [Service gap: Un availability of Laboratory and diagnostic service] 
No memos

Health insurance has a great advantages for those who have low income. For instance, I am not a health insurance member. But my family are using it. But there are many problems regarding its utilization. For instance, when a doctor prescribe expensive medicines, pharmacists refer us to private pharmacies even though most of the community member do not afford to buy from private pharmacies. Indeed, health workers do not want to admit critical patients if they are member of health insurance. But for those who are not member of health insurance, health workers make favor, provide them priority including admission

______________________________________________________________________

Code: PB_CBHI: FREQUENT health seeking compared to not members {1-0}~

P 6: FGdD_Female_ treatment.docx - 6:40 [I have several visits day and ..]  (177:177)   (Super)
Codes:	[PB_CBHI: FREQUENT health seeking compared to not members] 
No memos

 I have several visits day and evening. In the last three years, I have received different drugs both tablet and injection drugs without discontinuity. Those who do not know the benefits of insurance say 'What is the importance of Insurance?'  “But for me,  no one can benefit as much as the health insurance supports me. Even my mother and father can't support us like the health insurance has supported me. 

______________________________________________________________________

Code: PB_CBHI: good package of service {0-0}
______________________________________________________________________

Code: PB_CBHI: good pakage of service (admission) {2-0}

P 6: FGdD_Female_ treatment.docx - 6:5 [We cannot finshed the insuranc..]  (127:127)   (Super)
Codes:	[Attitude toward CBHI: good and eager to renew] [PB_CBHI:  referal service] [PB_CBHI: good pakage of service (admission)] [PB_CBHI: No out of pocket expenditure] 
No memos

We cannot finshed the insurance benefit by listing it within this limited time. At the start of insurance in the nation, most people fear to engage in insurance by saying our money would treat us. We do not want this membership card and call it a 'carton'.we can treat ourselves with no need from the government. However, after the community understood the advantages of health insurance, they immediately paid the annual premium and renewed the card within a given deadline and utilizing. Like fertilizer in its beginning most people take it forcibly we also had taken insurance. But now that we the farmers searching for health insurance like sugar because they are well aware of the importance of health insurance. We have been treated here but if it is beyond the health center we are referred to Dodola Hospital. During this referral service, we only paid for transportation and there is no anything we paid for the Dodola hospital. We never pay for admission, cards, and drugs. So the importance of health insurance is so many to list.  So we would like to thank the government for providing such services for us.

P 7: FGDs_Men___ treatment.docx - 7:1 [Health insurance is what we ar..]  (108:108)   (Super)
Codes:	[PB_CBHI: good pakage of service (admission)] 
No memos

Health insurance is what we are in, which brings us a lot of benefits as we know. As a community... even as a whole community service, it gives us great benefits for ourselves. Rather than our contributions, the service it gives us is the greatest, I swear to Allah.

______________________________________________________________________

Code: PB_CBHI: good pakage of service (referal service) {1-0}

P 4: FGD_MEN_ treatment.docx - 4:9 [The community is using insuran..]  (119:119)   (Super)
Codes:	[PB_CBHI: good pakage of service (referal service)] [PB_CBHI: Minimal contribution and getting service for a year] 
No memos

The community is using insurance without any payment when they get sick. Our community is well involved in community based health insurance because we will receive services including higher referral services with a small contribution of one time per year. It makes anyone a member of health insurance because it costs a lot of money to go to the health facility for health service by your own expense.

______________________________________________________________________

Code: PB_CBHI: improved health seeking behaviour {0-0}~
______________________________________________________________________

Code: PB_CBHI: imroved health seeking behavior {1-0}

P 9: KII_ __Cluster_CBHI Desk KII.docx - 9:34 [But now, just because they are..]  (66:66)   (Super)
Codes:	[PB_CBHI: imroved health seeking behavior] 
No memos

But now, just because they are a member of CBHI, they will get the flu, no matter what, it's about going to health facilities. Because of this, the health facilities are now overcrowded. In the past, in the area where CBHI was started, they were asked separately to open their own entrance to encourage. It means to make the people come to the health facilities. But now it is not possible to distinguish. Since everyone is a member, for whom do we open? So, everyone is a member. Therefore, if people have any kind of fever or anything, they run to the health facilities with only their ID, and they are very interested.

______________________________________________________________________

Code: PB_CBHI: increased health seeking behavior {2-0}

P 4: FGD_MEN_ treatment.docx - 4:22 [Nowadays care seeking practice..]  (146:146)   (Super)
Codes:	[PB_CBHI: increased health seeking behavior] 
No memos

Nowadays care seeking practice of our community is good. Before insurance, many people would not go to the health facility because they could not afford it. They go to traditional medicine. Now he/she has insurance, so if he/she gets a little sick, he/she goes to the health facility quickly. There are two reasons why our community is good at seeking care. Firstly, he/she has insurance and secondly, he/she has understanding.

P 8: KII HEW_SCH__TREATMENT_TW.docx - 8:7 [Unlike in the past, if our com..]  (30:30)   (Super)
Codes:	[PB_CBHI: increased health seeking behavior] 
No memos

 Unlike in the past, if our community gets even the slightest illness, he has CBHI and goes for treatment in time. In our area, in the past, if someone got sick, they would not go to the health facility immediately to escape spending money. Now, since our communities are members of CBHI, even if they get a headache, they run to the health facility with their CBHI.

______________________________________________________________________

Code: PB_CBHI: increased health seeking behavior by community {1-0}

P 3: FGD_Men__April 08,2024_.docx - 3:26 [There is no doubt regarding th..]  (111:111)   (Super)
Codes:	[Medicine unavailability in government facility:] [PB_CBHI: increased health seeking behavior by community] [Un availability of Laboratory and diagnostic service] 
No memos

There is no doubt regarding the improvement of health seeking practices, especially for women and our children; so many changes are there, now everyone can go to health facility without worrying about payment. The problem is that you cannot get enough medicine, and other services like X-Ray, and ultrasound. Even you cannot get specialist doctors; they sit in their own clinic, they are not available in government hospital even for an hour. If you want to discuss about your health with specialist, you have to go to their private clinic. They collect up to 5000 birr from single person. Visiting health facility has no value if you cannot get medicine for your disease. Another problem is that they never reimburse your expenses that you paid to buy medicine from private clinic or they amount you paid for X-Ray service at private clinic. They informed us that the government ordered them not to reimburse any expenses except for the receipt provided from Red Cross pharmacy.

______________________________________________________________________

Code: PB_CBHI: insurance members more respected treated firs {1-0}

P 2: FGD_Men___TREATMENT.docx - 2:35 [Think those who have insurance..]  (180:180)   (Super)
Codes:	[PB_CBHI: insurance members more respected treated firs] 
No memos

 Think those who have insurance are more respected and get service because they show up with their card and are treated immediately but those who do not have insurance requested to bring cards and other processes and take extra time to wait in a queue

______________________________________________________________________

Code: PB_CBHI: Minimal contribution and getting service for a year {4-0}

P 4: FGD_MEN_ treatment.docx - 4:3 [we are receiving many services..]  (111:111)   (Super)
Codes:	[PB_CBHI: Minimal contribution and getting service for a year] 
No memos

we are receiving many services by contributing a small amount of money. In the past, our poor people were suffering because of lack of money.

P 4: FGD_MEN_ treatment.docx - 4:4 [All households in our kebele h..]  (112:112)   (Super)
Codes:	[PB_CBHI: Minimal contribution and getting service for a year] 
No memos

 All households in our kebele have become members of community based health insurance. Because they said insurance benefits us. Everyone is a member of community health insurance because they are treated throughout the year with a small contribution. All households became a member of community based health insurance because everyone contributes a small amount of money to a health insurance member and gets the best service

P 4: FGD_MEN_ treatment.docx - 4:6 [Being a member of community ba..]  (114:114)   (Super)
Codes:	[PB_CBHI: Minimal contribution and getting service for a year] 
No memos

Being a member of community based health insurance has many benefits. For example, all members of my family pay a one-time fee and are served throughout the year. It is useful, no disadvantage. Because of this insurance, it will help you to save your money.

P 4: FGD_MEN_ treatment.docx - 4:9 [The community is using insuran..]  (119:119)   (Super)
Codes:	[PB_CBHI: good pakage of service (referal service)] [PB_CBHI: Minimal contribution and getting service for a year] 
No memos

The community is using insurance without any payment when they get sick. Our community is well involved in community based health insurance because we will receive services including higher referral services with a small contribution of one time per year. It makes anyone a member of health insurance because it costs a lot of money to go to the health facility for health service by your own expense.

______________________________________________________________________

Code: PB_CBHI: nearby health service and referal {1-0}

P 6: FGdD_Female_ treatment.docx - 6:11 [if someone has insurance can g..]  (135:135)   (Super)
Codes:	[PB_CBHI: nearby health service and referal] 
No memos

if someone has insurance can get service from here (the nearest HC) to referral. It was always told to us members of insurance. But those not considering government calls may miss the opportunity.

______________________________________________________________________

Code: PB_CBHI: nice service {1-0}

P 7: FGDs_Men___ treatment.docx - 7:25 [t is true that the service we ..]  (158:158)   (Super)
Codes:	[PB_CBHI: nice service] [Premium contribution: majority are poor] [premium contribution: not comparable with incomeaffordable] 
No memos

t is true that the service we received from the government is nice. On the other hand, the income we get is not enough for the current money. Ninety percent of these people have nothing. There is no need to add any more. we are depending on straw, selling straw to live. That's why, I don't think the economy we're responsible for now is enough here

______________________________________________________________________

Code: PB_CBHI: No out of pocket expenditure {2-0}

P 2: FGD_Men___TREATMENT.docx - 2:6 [Then we learned the importance..]  (121:121)   (Super)
Codes:	[Attitude SS_CBHI: positive, lestds the government continue it] [PB_CBHI: No out of pocket expenditure] 
No memos

Then we learned the importance of insurance and recommended others to be members of insurance. We all are enrolled in membership. We are happy with the current payment classification that considers the lower income as a low income, the middle income as his middle income, and the higher income as his income. We are happy, that we cannot pay from our pocket when we are sick but using our membership ID. 

P 6: FGdD_Female_ treatment.docx - 6:5 [We cannot finshed the insuranc..]  (127:127)   (Super)
Codes:	[Attitude toward CBHI: good and eager to renew] [PB_CBHI:  referal service] [PB_CBHI: good pakage of service (admission)] [PB_CBHI: No out of pocket expenditure] 
No memos

We cannot finshed the insurance benefit by listing it within this limited time. At the start of insurance in the nation, most people fear to engage in insurance by saying our money would treat us. We do not want this membership card and call it a 'carton'.we can treat ourselves with no need from the government. However, after the community understood the advantages of health insurance, they immediately paid the annual premium and renewed the card within a given deadline and utilizing. Like fertilizer in its beginning most people take it forcibly we also had taken insurance. But now that we the farmers searching for health insurance like sugar because they are well aware of the importance of health insurance. We have been treated here but if it is beyond the health center we are referred to Dodola Hospital. During this referral service, we only paid for transportation and there is no anything we paid for the Dodola hospital. We never pay for admission, cards, and drugs. So the importance of health insurance is so many to list.  So we would like to thank the government for providing such services for us.

______________________________________________________________________

Code: PB_CBHI: No out of pocket payment {5-0}

P 2: FGD_Men___TREATMENT.docx - 2:12 [In the past, we resisted being..]  (128:128)   (Super)
Codes:	[Attitude SS_CBHI: positive, lestds the government continue it] [PB_CBHI: No out of pocket payment] 
No memos

In the past, we resisted being members of health insurance without knowing its importance. For a long time, I did not understand the benefits of it, but last summer I fully understood health insurance benefits because if you are a member of it, you can get services like for registration card, and drugs from health facilities without payment. If the drugs are not available at health facilities there is a community service pharmacy in the town. You can get drugs from there without payment. But if you are not, you may buy drugs and everything from a private clinic with a huge amount of money. The second point is in the previous all individuals paid the same amount of money 410 birr. But now contribution is considered the capacity of individuals to pay which is a very important measurement taken by the government. 

P 2: FGD_Men___TREATMENT.docx - 2:15 [we are very happy about the he..]  (136:136)   (Super)
Codes:	[PB_CBHI: No out of pocket payment] 
No memos

we are very happy about the health insurance that the government implemented for us. After the implementation of health insurance, many of our people get services and benefits. Those who know the importance of insurance benefited more. Because when the insured person has to go to the health facility, he gets the service needed immediately only by showing the insurance card. So it is very important and it was based on our interest to be a member of insurance. They are informed of the importance of health insurance by health professionals and we enrolled by our interest and benefit now.  

P 4: FGD_MEN_ treatment.docx - 4:7 [ou will save money on medicine..]  (116:116)   (Super)
Codes:	[PB_CBHI: No out of pocket payment] 
No memos

ou will save money on medicines when we go to the health facility. So you will also learn how much money you have saved because of this insurance. So it has benefits but, no disadvantage.

P 6: FGdD_Female_ treatment.docx - 6:1 [In the beginning, we heard tha..]  (125:125)   (Super)
Codes:	[Attitude SS_CBHI: Supportive and recomend] [PB_CBHI:  inclussion of all family memebers for the service] [PB_CBHI: No out of pocket payment] [Premium contribution: fair: equivalent with  one time service at private] 
No memos

In the beginning,  we heard that health insurance be served to get health care without payment. They told us once we paid it would be servi for ourselves and our family. we paid for it and we received receipts based on our payment. And that individual who cannot able to pay can get a free membership. There are three different categories of payment schemes. So the health insurance is very important. The amount we pay for a membership that is served for a year for us and our children may be equivalent to the payment we pay at one visit of service at a private clinic.

P 7: FGDs_Men___ treatment.docx - 7:5 [Before now, a person who has n..]  (114:114)   (Super)
Codes:	[PB_CBHI: No out of pocket payment] 
No memos

Before now, a person who has no money at hand, may not go to health facility to get services when getting sick. If you are a member of health insurance, you can go to health facility as you already paid. In the past, even drug will be in short supply. Now that the pharmacy was open, there is no problem. I say they served us well, absolutely beautiful.

______________________________________________________________________

Code: PB_CBHI: No selling cattle for health expense {1-0}

P 4: FGD_MEN_ treatment.docx - 4:8 [we are getting our health. Bef..]  (117:117)   (Super)
Codes:	[PB_CBHI: better health] [PB_CBHI: No selling cattle for health expense] 
No memos

we are getting our health. Before this insurance started, we used to sell our cattle and get treatment, but now there is insurance and we are using it properly.

______________________________________________________________________

Code: PB_CBHI: No worries if money not available {1-0}

P 2: FGD_Men___TREATMENT.docx - 2:3 [So it is paid once and all the..]  (113:113)   (Super)
Codes:	[PB_CBHI: No worries if money not available] 
No memos

 So it is paid once and all the family members can get service when they get ill without worrying about the shortage of money.

______________________________________________________________________

Code: PB_CBHI: promoting the benefit to others {1-0}

P 2: FGD_Men___TREATMENT.docx - 2:14 [we advocate for non-insurance ..]  (133:133)   (Super)
Codes:	[PB_CBHI: promoting the benefit to others] 
No memos

we advocate for non-insurance to be enrolled in insurance by advising the importance of as compared to its annual payment. Kebele helps those poor and all are accepted it because of its benefit. Those has no insurance card may referred to a private hospital but those who have insurance get service from hospitals or health centers and also we are encouraging each other to be members of insurance.

______________________________________________________________________

Code: PB_CBHI: protect from expensive payment {1-0}

P 3: FGD_Men__April 08,2024_.docx - 3:10 [Previously, I was member of he..]  (93:93)   (Super)
Codes:	[Discrulination of insurance members] [long waiting time] [Medicine unavailability in government facility] [PB_CBHI: protect from expensive payment] 
No memos

Previously, I was member of health insurance; however, this year I do not want to be a member, because the service that I could get by this membership is not that much, there is no medicine in the government hospital. Not only medicine; as my friend previously raised, they also not permit X-Ray, and ultrasound services for insurance members. When an insurance member sent to X-Ray or ultrasound room, the individuals work in those room intentionally give extended appointment for the service. Imagine how a person who came from rural area wait three or four days to get these services. They [health workers] give you alternatives; if you can wait for long time, you can get these services free of charge by your insurance service, however, if you want it by the same day the request written, you have to go to the private clinics [mentioned the clinics by name]. If these problems resolved, insurance service is a relief for our community especially our rural community. I have good awareness about insurance service; I have no problem of awareness, I decided to drop out from membership due to inadequate services and mistreatment by health workers for insurance members. Even in my opinion, rural community have good awareness about the benefits of health insurance service. Even I learnt about insurance service from a person who came from rural area to seek health service for his child; fortunately, my child and his child admitted to the same room in one hospital in Addis Ababa, at the time, he was member of health insurance and I was not, and he told me about it in detail. Even he helped me to collect some of expensive medicine for my child using his insurance book. 

______________________________________________________________________

Code: PB_CBHI: protect from un necessary expense {1-0}

P 3: FGD_Men__April 08,2024_.docx - 3:3 [Insurance program is good to p..]  (89:89)   (Super)
Codes:	[CBHI_SS challenge: high fee for middle group] [Medicine unavailability in government facility] [PB_CBHI: protect from un necessary expense] 
No memos

Insurance program is good to protect our community from unnecessary expenses. However, this year the amount of payment has increased by more than double, which makes it difficult for many people especially poor households cannot pay for it despite they have interest to become member. Another major problem with health insurance, especially exacerbated this year is about unavailability of medicine from pharmacy of government health facilities. People are not getting medicines; they are being obligated to buy from private pharmacy at high cost. Sometimes, they may get from Red Cross pharmacy; however, government must work to improve the availability of medicine to sustain this health insurance

______________________________________________________________________

Code: PB_CBHI: reduce out of pocket payment {1-0}

P 5: FGD_Women__treatment.docx - 5:23 [eople pay if they have money b..]  (145:145)   (Super)
Codes:	[PB_CBHI: reduce out of pocket payment] 
No memos

eople pay if they have money because it is about their health. There are people has nothing to eat. They can't afford because of inflation.  To tell you the truth, if we were paying out of pocket, the amount we pay to become a member of health insurance is less than the cost of one visit. For instance, I am Asthmatic patient. Once upon a time, I went health center for follow up. They ordered me to buy a drug from private pharmacy. The cost of that drug was 1600 birr which is higher than the health insurance renewal premium. Thus, it is important to be member of health insurance. But the health workers are not respectful and it should be corrected.

______________________________________________________________________

Code: PB_CBHI: reduced out of pocket expenditure {1-0}

P 4: FGD_MEN_ treatment.docx - 4:1 [We have used community based h..]  (109:109)   (Super)
Codes:	[PB_CBHI: reduced out of pocket expenditure] [PB_CBHI: Rich and poor utilize the service] 
No memos

We have used community based health insurance for a long time. We have been using it since the contribution was 190 birr. When there was no insurance, the poor sold his/her farm and was treated with it. Since insurance came to us, there is no such thing as this rich person or this is poor, everyone uses insurance because they have insurance. Earlier there was a problem with the supply of medicines and now the service has improved. So, now we use insurance when we seek care. Health insurance has great benefits, and we receive great services for a small one-time contribution.

______________________________________________________________________

Code: PB_CBHI: referal services {1-0}~

P 7: FGDs_Men___ treatment.docx - 7:31 [Yes. It has an impact. The imp..]  (174:174)   (Super)
Codes:	[PB_CBHI: referal services] 
No memos

Yes. It has an impact. The impact of this is that the health insurance now passes on the referral even if he is unable to provide services here. The body that is not a member here is in a serious trouble. A private clinic is asking for anything as they want. He may be affected by his economic resources and health; he does not get prompt services. The non-member is at a disadvantage, at a great disadvantage. The members have been liberated. That's what it is.

______________________________________________________________________

Code: PB_CBHI: reimbursement of emergency payements {1-0}

P 6: FGdD_Female_ treatment.docx - 6:19 [in our village, somebody is cr..]  (140:140)   (Super)
Codes:	[PB_CBHI: reimbursement of emergency payements] 
No memos

in our village, somebody is critically ill and admitted to a hospital. In the beginning, he did not have an insurance card but after being exposed to costs he got an insurance card from here, and the money he expence refunded after he got insurance. At first, he was admitted for an emergency and reimbursed. So this is one of the benefits of insurance. 

______________________________________________________________________

Code: PB_CBHI: reimbursement of weekened payment {2-0}~

P 1: _FGD_WOMEN_TREATMENT_TW.docx - 1:18 [people are not aware of many t..]  (111:111)   (Super)
Codes:	[PB_CBHI: reimbursement of weekened payment] [Suggestions to improve the pilot : If fee increased avail drugs] [Weakness and challenges SS-CBHI implementation : poor awareness creation] 
No memos

people are not aware of many things including the increase in the price of medicines, the refund of their money if they receive a stamped receipt when they buy medicines from outside. In our health center, if you buy medicine from the Red Cross in prescribing from the health center, you can get your money back by showing the receipt. In my opinion, the CBHI fees have increased because the drugs have increased. Once the fees increased, medicines should be available to us at the health center.

P 1: _FGD_WOMEN_TREATMENT_TW.docx - 1:19 [y experience with reimbursemen..]  (112:112)   (Super)
Codes:	[PB_CBHI: reimbursement of weekened payment] 
No memos

y experience with reimbursement is that we went to the health center one Sunday for a baby treatment and paid for it while we had CBHI. On Monday they checked all of our money from the receipt and returned it to us.

______________________________________________________________________

Code: PB_CBHI: Relief for chronic illness patients {2-0}

P 3: FGD_Men__April 08,2024_.docx - 3:2 [magine, if those persons would..]  (88:88)   (Super)
Codes:	[PB_CBHI: Relief for chronic illness patients] 
No memos

magine, if those persons would not become insurance member, they could not pay for health services and could not even get treatment or medicine for their disease. After witnessing the benefits that individuals with chronic diseases have gained from enrolling in health insurance, other community members who are not afflicted with any illnesses are also joining health insurance.

P 5: FGD_Women__treatment.docx - 5:5 [Health insurance is highly hel..]  (117:117)   (Super)
Codes:	[PB_CBHI: Relief for chronic illness patients] [Service strength: friendly and respect ful health workers] [service strength: laboratory available] 
No memos

Health insurance is highly helped me. Using my green card, I am taking medication for diabetes mellitus, cholesterol, nerve damage, and hypertension. The problem is that sometimes I do not get all medications from the hospital. Health professionals are friendly and treat us with a great deal of respect. They conduct laboratory tests while we are being monitored. In order to cut down on waiting periods, we occasionally choose not to provide samples for laboratory investigations and instead to pick up our drugs and head back home. However, our doctors persuade us to give a sample for laboratory examination by emphasizin

______________________________________________________________________

Code: PB_CBHI: renewal reduce OOP {1-0}

P 2: FGD_Men___TREATMENT.docx - 2:28 [our colleague paid many birrs ..]  (163:163)   (Super)
Codes:	[PB_CBHI: renewal reduce OOP] 
No memos

our colleague paid many birrs for medical treatment the day before yesterday in Sheno town due to her problems of not having renewed insurance even though she paid for it.

______________________________________________________________________

Code: PB_CBHI: Rich and poor utilize the service {1-0}

P 4: FGD_MEN_ treatment.docx - 4:1 [We have used community based h..]  (109:109)   (Super)
Codes:	[PB_CBHI: reduced out of pocket expenditure] [PB_CBHI: Rich and poor utilize the service] 
No memos

We have used community based health insurance for a long time. We have been using it since the contribution was 190 birr. When there was no insurance, the poor sold his/her farm and was treated with it. Since insurance came to us, there is no such thing as this rich person or this is poor, everyone uses insurance because they have insurance. Earlier there was a problem with the supply of medicines and now the service has improved. So, now we use insurance when we seek care. Health insurance has great benefits, and we receive great services for a small one-time contribution.

______________________________________________________________________

Code: PB_CBHI: saved from out of pocket expense {2-0}

P 3: FGD_Men__April 08,2024_.docx - 3:1 [After this health insurance st..]  (88:88)   (Super)
Codes:	[PB_CBHI: saved from out of pocket expense] 
No memos

After this health insurance started in our area, our community has gotten so many advantages; the advantages of health insurance is very clear; all community members, including poor, ,medium and rich individuals are using health insurance and saved from expenses. Health insurance serves all our community equally and we also getting equal advantages from it. In my opinion, it [CBHI membership] does not reach all individuals; only those who have seen or heard its advantage go to kebele and seek the CBHI membership. A little bit, there is a difference this year; I think this year majority of our community become insurance member

P 6: FGdD_Female_ treatment.docx - 6:30 [here are poor people and newly..]  (155:155)   (Super)
Codes:	[PB_CBHI: saved from out of pocket expense] 
No memos

here are poor people and newly married people: those people better engage in insurance because they do not have money in case of sickness. Now everybody well now the importance of insurance through experience from other users. For example, an elder woman who middle-level categorization was referred to Shashamanne Hospital. At Shashame she was taken to a private clinic and requested to pay 15,000 birr. But she did not have 15,000, even though she could not afford 5 thousand. Then she back here and became a member of the insurance by paying 720 birr. After having insurance she was treated freely and saved 15,000 birr. This happened in this year. So each community learns from such types of cases

______________________________________________________________________

Code: PB_CBHI: timely  health care service {1-0}

P 6: FGdD_Female_ treatment.docx - 6:6 [If we have insurance we can ge..]  (127:127)   (Super)
Codes:	[PB_CBHI: timely  health care service] 
No memos

If we have insurance we can get service within a minute as everything on the insurance cards regardless of hospital or health center. 

______________________________________________________________________

Code: PB_CBHI: Women and children early treatment seeking {0-0}
______________________________________________________________________

Code: PB_CBHI: women and children easily acces to health care service {2-0}

P 5: FGD_Women__treatment.docx - 5:30 [Women and children may not hav..]  (158:158)   (Super)
Codes:	[PB_CBHI: women and children easily acces to health care service] 
No memos

Women and children may not have money to seek health care. But if they are member of health insurance, they can easily access health services without concern about money. Thus, it highly contribute to the health of children and women.

P 9: KII_ __Cluster_CBHI Desk KII.docx - 9:35 [Whether the father is there or..]  (70:70)   (Super)
Codes:	[PB_CBHI: women and children easily acces to health care service] [PB_CBHI: women empowerment] 
No memos

Whether the father is there or not, if the wife is sick, if one of the children in the family is sick, only this person has the ID, because the fee has paid once a year, so she runs to the health facilities. Therefore, children and women have gained a lot of freedom in terms of treatment. But before, it was under the shadow of husband. Even if he had money, she could not go to a medical institution without his permission. But now, it will create a situation where they can go freely with their ID, get treated, get the service and return.

______________________________________________________________________

Code: PB_CBHI: women empowerment {2-0}

P 8: KII HEW_SCH__TREATMENT_TW.docx - 8:8 [When the child is sick, there ..]  (34:34)   (Super)
Codes:	[PB_CBHI: women empowerment] 
No memos

When the child is sick, there is no waiting for the husband but to take the CBHI immediately to the health center for treatment. So the health of mothers and children has been well maintained since the introduction of this CBHI

P 9: KII_ __Cluster_CBHI Desk KII.docx - 9:35 [Whether the father is there or..]  (70:70)   (Super)
Codes:	[PB_CBHI: women and children easily acces to health care service] [PB_CBHI: women empowerment] 
No memos

Whether the father is there or not, if the wife is sick, if one of the children in the family is sick, only this person has the ID, because the fee has paid once a year, so she runs to the health facilities. Therefore, children and women have gained a lot of freedom in terms of treatment. But before, it was under the shadow of husband. Even if he had money, she could not go to a medical institution without his permission. But now, it will create a situation where they can go freely with their ID, get treated, get the service and return.

______________________________________________________________________

Code: PB_CBHI:Service satisfaction {1-0}

P 2: FGD_Men___TREATMENT.docx - 2:9 [currently we have a member of ..]  (122:122)   (Super)
Codes:	[Attitude SS_CBHI: positive, lestds the government continue it] [PB_CBHI:Service satisfaction] 
No memos

 currently we have a member of insurance and we treated using this insurance card when we ailed. The government profoundly supports this in different aspects because health insurance payment is based on an individual's capacity to pay both for lower and higher income. So I am mentally satisfied with it. Indeed, the government does not belong to only one individual but to all the country's individuals. But except for some individuals who ignored themselves, insurance has an important in a different aspect because in the past we carried patients using local beds (qareeza), but now, we can call for an Ambulance using call service numbers, by expressing the case that happened then the patient is immediately taken to the hospital for survive. Specially for laboring mothers, if possible it can be handled here (woreda) or referred to Dabra-birhane hospital. So we are very happy.

______________________________________________________________________

Code: PB_CBHI_: insurance members more respected and treated first {1-0}

P 2: FGD_Men___TREATMENT.docx - 2:36 [The person that has insurance ..]  (181:181)   (Super)
Codes:	[PB_CBHI_: insurance members more respected and treated first] 
No memos

The person that has insurance served first. And those who did not have ordered to bring cards. This is the strongest part of insurance they show. However, if it is just now, we cannot be confident that they treat us only as having insurance without having money in our pockets. What every severity of the case, we cannot go for health care without having money on our hands. Because either drug may not be available or they may refer us to another facility if the case is beyond their capacity.

______________________________________________________________________

Code: PB_ss_CBHI: good package of service {5-0}~

P 1: _FGD_WOMEN_TREATMENT_TW.docx - 1:2 [In my opinion, the CBHI progra..]  (92:92)   (Super)
Codes:	[PB_ss_CBHI: good package of service] [PB_SS_CBHI: profitable] [PB_SS_CBHI: tax based] [Service gap: Medicine unavailability ingovernment facility] [Service gap: Poor reception of health workers: denial for repeat service] 
No memos

In my opinion, the CBHI program is excellent. They even made us pay for it the same way we pay taxes. As a region, we are using CBHI for many services, such as dentistry and kidney disease. So the service we get is much more than the fees we pay. However, occasionally, if not all, some health professionals do not kindly welcome people. In contrast, there are those who are very sympathetic and kindly welcoming. In terms of medications, there is also frequent prescribing of many medications outside of the health facility.

P 1: _FGD_WOMEN_TREATMENT_TW.docx - 1:6 [Later, however, I learned that..]  (95:95)   (Super)
Codes:	[HWB: respect] [PB_ss_CBHI: good package of service] [Service gap: Health worker behaviour; no respect] 
No memos

Later, however, I learned that it was useful when I started using it and saw those who had used it even for the surgical treatment. So this CBHI is very useful. With regard to professional reception, not everyone has the same characteristics. This means that there are those who respect others and those who do not.

P 1: _FGD_WOMEN_TREATMENT_TW.docx - 1:9 [People like me who have seen t..]  (99:99)   (Super)
Codes:	[Over expectation] [PB_ss_CBHI: good package of service] [PB_SS_CBHI: tax based] [Poor reception of health workers:] 
No memos

People like me who have seen the benefits at various times will not hesitate to become members, even if they lose one day's service. However, clients, especially those who come for emergencies and lose services, may become frustrated and withdraw from membership. Some clients think that they should never lose service after paying for CBHI due to a lack of understanding or advice and are disappointed when they lose a single service. Additionally, those who have repeatedly lost treatment and are disappointed with the reception of professionals may also leave the membership. In our kebele, to prevent the loss of members, HEWs provide advice, and there is a compulsory payment of CBHI with taxes.

P 1: _FGD_WOMEN_TREATMENT_TW.docx - 1:14 [I have been using this CBHI fo..]  (107:107)   (Super)
Codes:	[PB_ss_CBHI: good package of service] 
No memos

I have been using this CBHI for 6 years without interruption. I and my family have been using all the services of CBHI and have not had any problems. We were well served and have no complaints.

P 6: FGdD_Female_ treatment.docx - 6:15 [. It is very important for the..]  (139:139)   (Super)
Codes:	[PB_CBHI:  referal service] [PB_ss_CBHI: good package of service] 
No memos

. It is very important for the laboring mother, to perform surgery through referral of a patient to another far health facility. So it has many benefits. B

______________________________________________________________________

Code: PB_SS_CBHI: good package of service(x-ray) {1-0}

P 6: FGdD_Female_ treatment.docx - 6:3 [After the implementation of he..]  (126:126)   (Super)
Codes:	[PB_SS_CBHI: good package of service(x-ray)] 
No memos

After the implementation of health insurance, there is progress. Really the person who was not treated in the previous, currently, has been treated by doing different investigations like x- ray as where he wants. This is the support done by the government. So it has many advantages. It has benefits for us. Many family members have benefited depending on their family size.

______________________________________________________________________

Code: PB_ss_CBHI: good package of service_1 {0-0}
______________________________________________________________________

Code: PB_SS_CBHI: poor package of service {0-0}~
______________________________________________________________________

Code: PB_SS_CBHI: profitable {1-0}

P 1: _FGD_WOMEN_TREATMENT_TW.docx - 1:2 [In my opinion, the CBHI progra..]  (92:92)   (Super)
Codes:	[PB_ss_CBHI: good package of service] [PB_SS_CBHI: profitable] [PB_SS_CBHI: tax based] [Service gap: Medicine unavailability ingovernment facility] [Service gap: Poor reception of health workers: denial for repeat service] 
No memos

In my opinion, the CBHI program is excellent. They even made us pay for it the same way we pay taxes. As a region, we are using CBHI for many services, such as dentistry and kidney disease. So the service we get is much more than the fees we pay. However, occasionally, if not all, some health professionals do not kindly welcome people. In contrast, there are those who are very sympathetic and kindly welcoming. In terms of medications, there is also frequent prescribing of many medications outside of the health facility.

______________________________________________________________________

Code: PB_SS_CBHI: profitable_1 {0-0}
______________________________________________________________________

Code: PB_SS_CBHI: tax based {2-0}

P 1: _FGD_WOMEN_TREATMENT_TW.docx - 1:2 [In my opinion, the CBHI progra..]  (92:92)   (Super)
Codes:	[PB_ss_CBHI: good package of service] [PB_SS_CBHI: profitable] [PB_SS_CBHI: tax based] [Service gap: Medicine unavailability ingovernment facility] [Service gap: Poor reception of health workers: denial for repeat service] 
No memos

In my opinion, the CBHI program is excellent. They even made us pay for it the same way we pay taxes. As a region, we are using CBHI for many services, such as dentistry and kidney disease. So the service we get is much more than the fees we pay. However, occasionally, if not all, some health professionals do not kindly welcome people. In contrast, there are those who are very sympathetic and kindly welcoming. In terms of medications, there is also frequent prescribing of many medications outside of the health facility.

P 1: _FGD_WOMEN_TREATMENT_TW.docx - 1:9 [People like me who have seen t..]  (99:99)   (Super)
Codes:	[Over expectation] [PB_ss_CBHI: good package of service] [PB_SS_CBHI: tax based] [Poor reception of health workers:] 
No memos

People like me who have seen the benefits at various times will not hesitate to become members, even if they lose one day's service. However, clients, especially those who come for emergencies and lose services, may become frustrated and withdraw from membership. Some clients think that they should never lose service after paying for CBHI due to a lack of understanding or advice and are disappointed when they lose a single service. Additionally, those who have repeatedly lost treatment and are disappointed with the reception of professionals may also leave the membership. In our kebele, to prevent the loss of members, HEWs provide advice, and there is a compulsory payment of CBHI with taxes.

______________________________________________________________________

Code: PB_SS_CBHI: tax based_1 {0-0}
______________________________________________________________________

Code: Perceived benefits of CBHI: free service {2-0}~

P 1: _FGD_WOMEN_TREATMENT_TW.docx - 1:7 [his means that anyone who has ..]  (97:97)   (Super)
Codes:	[CBHI _challenge: lack of understanding] [CBHI_challenge: not only professionals] [PB_CBHI: developed passion] [PB_CBHI: eager to renew] [Perceived benefits of CBHI: free service] 
No memos

his means that anyone who has seen and heard of its benefits is eager to renew their membership. For example, in the last year, a child of a relative of mine who is a member of CBHI was lying in bed for surgery at Tulu Bolo. The child was treated free of all service fees, including food. In the same ward, there was a man without CBHI being treated, and he was treated and discharged at great expense for similar services as that of a child of my relative. Since then, I have developed a passion for the CBHI. I also told people I knew about the benefits and made them interested. So in my opinion, it is a lack of understanding, but the problem of professionals alone does not prevent renewing the membership.

P 1: _FGD_WOMEN_TREATMENT_TW.docx - 1:34 [The CBHI helped us a lot becau..]  (135:135)   (Super)
Codes:	[Perceived benefits of CBHI: free service] 
No memos

 The CBHI helped us a lot because even if we don't have the money at the moment we got sick, we go to the health facility and get treatment by CBHI.

______________________________________________________________________

Code: Perceived benefits of CBHI: No borrowed money {1-0}

P 1: _FGD_WOMEN_TREATMENT_TW.docx - 1:35 [CBHI was very helpful to us. I..]  (136:136)   (Super)
Codes:	[Perceived benefits of CBHI: No borrowed money] 
No memos

CBHI was very helpful to us. If we were sick in the past and didn't have money on our hands, we would take alms money and go for treatment. We would give the money we borrowed to the owner along with interest. But now because of CBHI has come, such things have stopped

______________________________________________________________________

Code: Perception_SS_CBHI: Insurance for poor {2-0}

P11: KII _Town HO head_ Treatment.docx - 11:12 [The perception of society on h..]  (30:30)   (Super)
Codes:	[Perception_SS_CBHI: Insurance for poor] 
No memos

The perception of society on health insurance, heterogeneity society, different economic statuses, and the perception of people on health insurance is considered poor people are some of the challenges of health insurance. Some individuals with income are more perceived to be treated at private facilities and consider the public facilities area of vaccination.

P16: KII__HEW_ _ _.docx - 16:12 [A person with little wealth (p..]  (27:27)   (Super)
Codes:	[Perception_SS_CBHI: Insurance for poor] [Perception_SS_CBHI: unaffordable direct payment] 
No memos

A person with little wealth (poor) pays on time. This means that by comparison, poor people pay first because they know that if they get sick, they will be charged a lot of money. Therefore, people with limited resources become members of community based health insurance first.

______________________________________________________________________

Code: Perception_SS_CBHI: unaffordable direct payment {1-0}

P16: KII__HEW_ _ _.docx - 16:12 [A person with little wealth (p..]  (27:27)   (Super)
Codes:	[Perception_SS_CBHI: Insurance for poor] [Perception_SS_CBHI: unaffordable direct payment] 
No memos

A person with little wealth (poor) pays on time. This means that by comparison, poor people pay first because they know that if they get sick, they will be charged a lot of money. Therefore, people with limited resources become members of community based health insurance first.

______________________________________________________________________

Code: Periodic evaluation {1-0}

P 9: KII_ __Cluster_CBHI Desk KII.docx - 9:23 [t is a condition of improving ..]  (50:50)   (Super)
Codes:	[Periodic evaluation] 
No memos

t is a condition of improving the quality of service in order to maintain it. The same will be evaluated and we now have a section called Provider. Periodic audit works, again comprehensive works. So, this is about readiness. How many health institutions are ready is evaluated every time, every six months. And what kind of status is it? These people are providing services to the society by buying the resources they need with the money they earn.

______________________________________________________________________

Code: phase by phase payment method {1-0}

P 9: KII_ __Cluster_CBHI Desk KII.docx - 9:44 [Otherwise, if we are always fo..]  (80:80)   (Super)
Codes:	[phase by phase payment method] 
No memos

Otherwise, if we are always forcing people to pay for them, I think that we are making the health insurance system of the society not to be sustained. The society has to open an account and save five-birr, ten birr or twenty birrs for CBHI and contribute to it until the end of the year. When people suddenly asked at that year they transfer that money without any burden. This will create a situation where you will transfer to that place and become a member.

______________________________________________________________________

Code: Poor people : excluded due to high fee {1-0}

P 3: FGD_Men__April 08,2024_.docx - 3:18 [During my role as a community ..]  (99:99)   (Super)
Codes:	[Poor people : excluded due to high fee] 
No memos

During my role as a community mobilizer for insurance payment, I had the opportunity to hear numerous complaints from the community, particularly regarding their inability to afford insurance premiums and their poor interest due to the lack of availability of medicines from government health facility. Many of our community are poor; especially those who live in the town have numerous expenses like home rent, water and electric bill, school payment, and expenses on food. Therefore, I practically heard and also know many poor individuals who have interest to become insurance member and could not pay the premium.

______________________________________________________________________

Code: Poor people : in ability to pay {1-0}

P 3: FGD_Men__April 08,2024_.docx - 3:19 [When we come to the payment am..]  (101:101)   (Super)
Codes:	[Poor people : in ability to pay] 
No memos

When we come to the payment amount, it increases by two fold from the previous year amount. May poor individuals are excluded from insurance service due to inability to pay despite the fact that they want to be member. 

______________________________________________________________________

Code: Poor people : unabile to pay {1-0}

P 7: FGDs_Men___ treatment.docx - 7:22 [There is a little bit limitati..]  (152:152)   (Super)
Codes:	[Poor people : unabile to pay] [Premium contribution challenge: famine] 
No memos

There is a little bit limitation in our society. In the context of the time of the, large percentage to people who has nothing in his hands. There is the poor, who have nothing to eat overnight. There are those who depend on straw, depend on trees and sell straw. There are those who don't have a single goat at home. There is one who doesn't have a single hen at home, to speak true. They are the ones who are helped by the neighbors. There is a great fa_re of the family upon them. So, the government has to think about this. We said there is low, there is extreme low. It would be nice if the lowest of the lowest could not pay seven hundred and the government should support in the future. We are a kind of in a famine area, there is not enough rainfall in this area. Majority of the society, ninety percent are poor, have nothing on their hands. If you enter this city, there is a dependency in this city style of ours. There is someone who doesn't have a home. There is a man who does have a hen at home with four, three families under them. Some took the leftover of chats and as they can't find it. If we told him to pay six hundred and seven hundred and we took him here and there, he might be worried about this. Even from time to time, awareness should be given to the community. After they got ideas from each other, they are getting understanding. I think it would be nice if it was conducted this way. Our economy and the payments there are not equal.

______________________________________________________________________

Code: Poor quality of service {2-0}~

P 2: FGD_Men___TREATMENT.docx - 2:4 [However, two things can hinder..]  (116:117)   (Super)
Codes:	[Poor quality of service] [Service gap: Medicine unavailability ingovernment facility] 
No memos

 However, two things can hinder being an insurance member: the first is the poor quality of service, and the second is the absence of drugs hinders the whole people from being enrolled in insurance. 
They complain “Why do I pay my money if I can't get anything from the government health facilities?” So they choose a private clinic for health care by user pay/directly paying.  

P 2: FGD_Men___TREATMENT.docx - 2:17 [Why some people are not engage..]  (140:140)   (Super)
Codes:	[Poor quality of service] [Poor reception of health workers: they play ball at working time] [Service gap: Medicine unavailability ingovernment facility] 
No memos

Why some people are not engaged in health insurance are:  first due to the quality of health service example, there was some improvement after the hospital commenced in the area, but if you go health center it's difficult to say you are looking for service rather than playing. First, the way they welcome is not good, as the issues already mentioned in this discussion, when you are looking for the health center, health workers are playing football, and they do not care about you. They sit in one place either busy with their mobile or doing some other playing. The way they welcomed me was not good. The second problem is the way they treat is not appropriate. They ask about your problems orally but they don't use devices for further investigation. Only Based on your complaint you report prescribe if and only if the drug is available in their facility. Moreover, after taking the medicine that was prescribed, there was no improvement for the patient because this disease was not well addressed then the patient decided to not visit the facility again and go to a private clinic. At a private clinic, he was well investigated and treated and become improved. So he said “Why do I pay for insurance?

______________________________________________________________________

Code: poor quality service {0-0}
______________________________________________________________________

Code: Poor reception of health workers {0-0}
______________________________________________________________________

Code: Poor reception of health workers: {9-0}

P 1: _FGD_WOMEN_TREATMENT_TW.docx - 1:9 [People like me who have seen t..]  (99:99)   (Super)
Codes:	[Over expectation] [PB_ss_CBHI: good package of service] [PB_SS_CBHI: tax based] [Poor reception of health workers:] 
No memos

People like me who have seen the benefits at various times will not hesitate to become members, even if they lose one day's service. However, clients, especially those who come for emergencies and lose services, may become frustrated and withdraw from membership. Some clients think that they should never lose service after paying for CBHI due to a lack of understanding or advice and are disappointed when they lose a single service. Additionally, those who have repeatedly lost treatment and are disappointed with the reception of professionals may also leave the membership. In our kebele, to prevent the loss of members, HEWs provide advice, and there is a compulsory payment of CBHI with taxes.

P 1: _FGD_WOMEN_TREATMENT_TW.docx - 1:10 [verybody wants to become a mem..]  (101:101)   (Super)
Codes:	[CBHI valued program] [Poor reception of health workers:] [Service gap: Medicine unavailability ingovernment facility] 
No memos

verybody wants to become a member of CBHI regardless of whether they are poor or rich. Although there is often a shortage of medicines and problems with professional's reception, many people in our kebele need this CBHI.

P 2: FGD_Men___TREATMENT.docx - 2:21 [As we mentioned in our discuss..]  (145:145)   (Super)
Codes:	[Poor reception of health workers:] [Service gap: Medicine unavailability ingovernment facility] 
No memos

As we mentioned in our discussion before, the things that are barriers to health insurance are for example the day before yesterday my mother had a monthly follow-up. No one can evaluate you when you go there. She came for her regular follow-up in the morning time. The health professionals told to her to wait until the afternoon 1:30 to 2: pm to get the service and she waited until that. Finally did not receive medicine and were told to buy drugs from private clinics which makes people decide not to be a member of insurance as already stated by another. And also my father bought hypertensive drugs from a private clinic because of unavailable drugs from a public health facility. If he wait for public facility drugs, he may not be alive today.  I swear to god, People said 'private clinic is better than a public facility.' How drugs are available in private and absent from the government? So they said 'How we can pay there (for insurance in the situation of the absence of drugs? This makes many people restricted from being members of health insurance. 

P 2: FGD_Men___TREATMENT.docx - 2:22 [Regarding the provision of ser..]  (147:147)   (Super)
Codes:	[delay referal] [Poor reception of health workers:] [Service gap: Medicine unavailability ingovernment facility] 
No memos

Regarding the provision of service, there are a lot of problems. There are many problems, many…many. For example in the month of September of this year, a pregnant mother bleeding like sheep on the floor and they allowed this lady to go by standing simply by hugging her hand when she transferred from one room to another room. It's better if they ask for assistance from others to carry this mother. Really, I cover her body with clothes ashamed of their job.  Complaints are always raised by people. But no one can pay attention to the complaints. Even though there is appraised work done on insurance, but there are many and always problems in service provisions. The community always...always… always... Raised service problems. The major community complaint on the government is that always raised in every meeting is service providers. Is that the government can't has no capacity to avail medicine? Let alone, he (the government) can't afford and is short of medicine, why workers don't properly and timely perform their job? Why they did not refer patients timely where the medicine was available rather than waiting for them the whole day and finally telling us about the unavailability of medicine? The 100% of community complaints remained without a solution to kebele level as already mentioned. 

P 2: FGD_Men___TREATMENT.docx - 2:24 [Really, health insurance has a..]  (149:149)   (Super)
Codes:	[Attitude toward CBHI: good] [Poor reception of health workers:] [Service gap: delay referal process] 
No memos

Really, health insurance has a lot of benefits. In another way, my complaint was with health professionals. For example, we took to laboring mother to a health facility and they told us to wait until midnight and back again. But mother suffered and was near to death. The person on duty was already asleep and after many knocks on his door, he woke up. After many repeated requests to evaluate the mother, he came and tried to evaluate but never considered the mother's pain and screamed at me by saying 'get out from me' We also requested them to refer to other facilities if it is beyond their capacity. Finally, after many struggles, they referred it to Debrabihan Hospital. When we arrived at the hospital, the Debrabiran doctors shouting me “Why are you delayed until such kind the mother suffered?” they condescended to me. finally, they saved that mother. So insurance is very essential but what shall we do? Are the health workers abusing us? This is our major probl

P 2: FGD_Men___TREATMENT.docx - 2:25 [In general, they (workers at a..]  (158:158)   (Super)
Codes:	[Poor reception of health workers:] 
No memos

In general, they (workers at a facility) do not properly serve us. The insurance supports us but the health workers at the facility never support us. They did not know what they doing. They missed some information on our medical records. Better if they are properly evaluated. 

P 2: FGD_Men___TREATMENT.docx - 2:37 [As we already said, the proble..]  (182:182)   (Super)
Codes:	[Poor reception of health workers:] [Service gap: Medicine unavailability ingovernment facility] 
No memos

As we already said, the problem was with workers not with the government. What does it mean to keep patients for several times without giving service? the patient may passed away while waiting for service which is very difficult for me to accept. Insurance is important for us but the problems are with workers who work at health facilities. For example, I went for health care many times but no one could give me any injectable medicine. I was ill many times but no one has given me injectable medicine so far. I am surprised that God has kept me by my existence regardless of being exposed to disease many times.  No one can be ill than me as my neighbor evident. As the frequent of disease, I may die. Even I referred to Debrabirhan. We know the health insurance is very important. But what made me angry was how private clinics are more avail drugs than government facilities. Is the government less than the private? Where does the private get drugs that they order us bought from private?  Really We are very angry about it. better such kind is improved. The way workers treat, drugs have to be improved for us. This is our major complaint. 

P 3: FGD_Men__April 08,2024_.docx - 3:25 [his is defamation; no one go t..]  (109:109)   (Super)
Codes:	[discrimination of insurance members: Professional hate us] [Poor reception of health workers:] 
No memos

his is defamation; no one go to health facility without illness. Do you believe we go to health facility without illness to face that all challenges? Let me tell you the truth, Insurance members and the beggars sitting in front of church are the same [kadhataan bataskaana fuuldura taa'u fi miseensi insuraansii fayyaa tokkodha]. Once they see that insurance card in your hand, they extremely hate you, they never greet you, and they ignore you at all. No one go to health facility without experiencing some illness or discomfort. Even the problems and experiences we shared with you on this discussion is not enough, you have to go to the rural community and ask them; I am sure many of them will cry while telling you the problems they faced in the health facilities. Rich individuals may not see what we are talking as problem because they have enough money to seek health service from private clinics. I know the problem in detail because I have experienced many challenges before becoming to insurance member and after I became insurance member. I have a child with chronic illness and faced all problems shared here by my friends

P 4: FGD_MEN_ treatment.docx - 4:12 [Sometimes there was a problem ..]  (126:126)   (Super)
Codes:	[Medicine unavailability in government facility] [Poor reception of health workers:] 
No memos

 Sometimes there was a problem of good reception by the professionals. People are concerned that there may be a shortage of drugs supplies. Some people may have no money and concerned the cash contribution required by the community based health insurance member. 

______________________________________________________________________

Code: Poor reception of health workers: denial of repeated client {1-0}

P 1: _FGD_WOMEN_TREATMENT_TW.docx - 1:30 [I have experience with health ..]  (129:129)   (Super)
Codes:	[Poor reception of health workers: denial of repeated client] 
No memos

I have experience with health professionals refusing to treat people when they go to the health facility frequently due to frequent health problems. For instance, last year, I got sick and went to the health center three times. But he said, “Did you come again?” And he refused to treat me by saying, “I wouldn't waste my time trying to treat you again and again.” I did go back and forth because I had chronic diseases like liver disease.

______________________________________________________________________

Code: Poor reception of health workers: no respect {1-0}

P 1: _FGD_WOMEN_TREATMENT_TW.docx - 1:32 [Lack of medical equipment and ..]  (132:132)   (Super)
Codes:	[Poor reception of health workers: no respect] 
No memos

Lack of medical equipment and lack of respect by health professionals prevents people from going to the health facility. In my opinion, I prefer to go to a private clinic because it gives you enough respect and care.

______________________________________________________________________

Code: Poor reception of health workers: they play ball at working time {2-0}

P 2: FGD_Men___TREATMENT.docx - 2:13 [in our previous meeting, the i..]  (129:129)   (Super)
Codes:	[Poor reception of health workers: they play ball at working time] 
No memos

in our previous meeting, the issue discussed is that “When someone suffers from an illness, the workers of health facility playing ball” was raised aggressively by many people many many... The Ganda cabine well noted the complaints of the community that were raised during the meeting but still, there is no solution taken. If someone gets an education expected to be progressed not retarded. If corrective action is taken against the wrongdoer, it would lessen for the others and the community will get services. This is what I think. There are always complaints but no answer to them

P 2: FGD_Men___TREATMENT.docx - 2:17 [Why some people are not engage..]  (140:140)   (Super)
Codes:	[Poor quality of service] [Poor reception of health workers: they play ball at working time] [Service gap: Medicine unavailability ingovernment facility] 
No memos

Why some people are not engaged in health insurance are:  first due to the quality of health service example, there was some improvement after the hospital commenced in the area, but if you go health center it's difficult to say you are looking for service rather than playing. First, the way they welcome is not good, as the issues already mentioned in this discussion, when you are looking for the health center, health workers are playing football, and they do not care about you. They sit in one place either busy with their mobile or doing some other playing. The way they welcomed me was not good. The second problem is the way they treat is not appropriate. They ask about your problems orally but they don't use devices for further investigation. Only Based on your complaint you report prescribe if and only if the drug is available in their facility. Moreover, after taking the medicine that was prescribed, there was no improvement for the patient because this disease was not well addressed then the patient decided to not visit the facility again and go to a private clinic. At a private clinic, he was well investigated and treated and become improved. So he said “Why do I pay for insurance?

______________________________________________________________________

Code: premium : difficult for some {0-0}
______________________________________________________________________

Code: Premium : need revision {0-0}
______________________________________________________________________

Code: Premium : requiere revision {1-0}

P 9: KII_ __Cluster_CBHI Desk KII.docx - 9:2 [I am concerned that the insura..]  (22:22)   (Super)
Codes:	[Concer: CBHI may fail due to lower poremium] [Premium : requiere revision] 
No memos

I am concerned that the insurance may be canceled tomorrow, so perhaps this matter should be carefully studied in the next revision of the price, and I do not think it is enough.

______________________________________________________________________

Code: Premium : require revision {1-0}~

P 9: KII_ __Cluster_CBHI Desk KII.docx - 9:3 [Even the society themselves as..]  (24:24)   (Super)
Codes:	[Premium : require revision] 
No memos

Even the society themselves ask is it enough to cover medical cost? May be when it is accumulated by the individual, the society knows. When someone is sick, he goes as far as selling the ox. How much is the price of ox today?  May be at least fifty thousand, sixty thousand.  Even it may not improve with this much expenses. They know the problem with this. Therefore, now I don't think they have much problem with the money.

______________________________________________________________________

Code: Premium collection challenge: similar time with tax collection {2-0}~

P 2: FGD_Men___TREATMENT.docx - 2:20 [This is the barriers community..]  (143:144)   (Super)
Codes:	[Attitude SS_CBHI: positive, lestds the government continue it] [Premium collection challenge: similar time with tax collection] [Weakness and challenges SS-CBHI implementation : missclasification] 
No memos

This is the barriers community and farmers' barriers to membership. In another way, the classification of payment that was categorized by the government is very good in classifying to lower, middle, and higher. It is based on their income, the presence of land for irrigation or not, the types of their house condition, and others that are very nice.  However, during classification, there was a mistake in classification. For example, those have to be categorized under poor/lower level classified under middle level and vs verses. But there were no problems with upper-class categorization. So it is better to correct those misclassification. 
The other suggestion that will be improved is the price of annual contribution. It is better if it reconsiders the paying capacity of individuals.  Since the health insurance contribution is paid with other expenses like taxation, the total it costs is above the price of a cattle (notify).   So it is difficult to pay in this situation. So I suggest some adjustments to the classification payment contribution.

P 6: FGdD_Female_ treatment.docx - 6:29 [During the payment of insuranc..]  (154:154)   (Super)
Codes:	[Premium collection challenge: similar time with tax collection] 
No memos

During the payment of insurance, other different types of payment packages come together like land taxation. For example, if someone came for land taxation he was also commanded to pay for insurance simultaneously. So it was a must to pay at that time. So those who do not have land taxation, there is no chance to be influenced to pay, unlike taxpayers. This and others make them too late to engage in insurance.

______________________________________________________________________

Code: Premium collection time: it is difficult it ovelaps with other payments {1-0}

P 5: FGD_Women__treatment.docx - 5:19 [Most of the time different con..]  (138:138)   (Super)
Codes:	[Premium collection time: it is difficult it ovelaps with other payments] [Premium contribution challenge: Age>16 asked independently to contribute] 
No memos

Most of the time different contributions overlap. Because of this, people may not afford the health insurance premium. The premium collectors say that how you can be indigent every year, you have to pay this year. The amount of contribution is really small. But people may not get the money at hand when requested to pay. The other problem is that if there are family members aged greater than sixteen years old, they request to pay the premium independently which is not right. Only the head of household (husband/wife) may have money and other family members may not have even though their age are greater than sixteen years old.  Those individuals may be students and may not have any source of income to pay health insurance. It is very difficult to afford the premium especially for youths of poor family.

______________________________________________________________________

Code: Premium contribution challenge: Age>16 asked independently to contribute {1-0}

P 5: FGD_Women__treatment.docx - 5:19 [Most of the time different con..]  (138:138)   (Super)
Codes:	[Premium collection time: it is difficult it ovelaps with other payments] [Premium contribution challenge: Age>16 asked independently to contribute] 
No memos

Most of the time different contributions overlap. Because of this, people may not afford the health insurance premium. The premium collectors say that how you can be indigent every year, you have to pay this year. The amount of contribution is really small. But people may not get the money at hand when requested to pay. The other problem is that if there are family members aged greater than sixteen years old, they request to pay the premium independently which is not right. Only the head of household (husband/wife) may have money and other family members may not have even though their age are greater than sixteen years old.  Those individuals may be students and may not have any source of income to pay health insurance. It is very difficult to afford the premium especially for youths of poor family.

______________________________________________________________________

Code: Premium contribution challenge: droped chat price {3-0}

P 4: FGD_MEN_ treatment.docx - 4:15 [Our community's income is main..]  (132:132)   (Super)
Codes:	[Attitude SS_CBHI: positive and beneficial] [Premium contribution challenge: droped chat price] 
No memos

Our community's income is mainly based on chat and now the price of chat has dropped and it is become challenge to pay. That's why some people say it's hard for us to contribute the insurance membership fee. How do I become the high payer when the committee ranks to high, medium and low payer households? Few people mention that I have not reached this level. However, the community knows that health insurance is beneficial and believes in it and becomes an insurance member.

P 4: FGD_MEN_ treatment.docx - 4:16 [ur community has willingness t..]  (134:134)   (Super)
Codes:	[Attitude SS_CBHI: positive and happy to contribute] [Premium contribution challenge: droped chat price] 
No memos

ur community has willingness to pay but it is challenging to pay due to undervalued chat price. So, the community's economy depends on chat revenue. However, we are being treated in many rounds with a one-time contribution. So it means our community is happy to contribute.

P 4: FGD_MEN_ treatment.docx - 4:21 [Most of our community has rene..]  (143:143)   (Super)
Codes:	[Premium contribution challenge: droped chat price] 
No memos

Most of our community has renewed community based health insurance membership. It will be improved by conducting community consultations to improve their renewal rate. This year the problem was that the price of chat had dropped and the community faces difficulty to pay. On the other hand, it is necessary to create public awareness.

______________________________________________________________________

Code: Premium contribution challenge: famine {1-0}

P 7: FGDs_Men___ treatment.docx - 7:22 [There is a little bit limitati..]  (152:152)   (Super)
Codes:	[Poor people : unabile to pay] [Premium contribution challenge: famine] 
No memos

There is a little bit limitation in our society. In the context of the time of the, large percentage to people who has nothing in his hands. There is the poor, who have nothing to eat overnight. There are those who depend on straw, depend on trees and sell straw. There are those who don't have a single goat at home. There is one who doesn't have a single hen at home, to speak true. They are the ones who are helped by the neighbors. There is a great fa_re of the family upon them. So, the government has to think about this. We said there is low, there is extreme low. It would be nice if the lowest of the lowest could not pay seven hundred and the government should support in the future. We are a kind of in a famine area, there is not enough rainfall in this area. Majority of the society, ninety percent are poor, have nothing on their hands. If you enter this city, there is a dependency in this city style of ours. There is someone who doesn't have a home. There is a man who does have a hen at home with four, three families under them. Some took the leftover of chats and as they can't find it. If we told him to pay six hundred and seven hundred and we took him here and there, he might be worried about this. Even from time to time, awareness should be given to the community. After they got ideas from each other, they are getting understanding. I think it would be nice if it was conducted this way. Our economy and the payments there are not equal.

______________________________________________________________________

Code: Premium contribution challenge: not by willingness {1-0}~

P 5: FGD_Women__treatment.docx - 5:16 [The contribution is not based ..]  (133:133)   (Super)
Codes:	[Premium contribution challenge: not by willingness] 
No memos

The contribution is not based on willingness. Rather it is forced payment. If we are able to contribute, we are really happy to participate. But we may not have money at hand during their contribution request. There are people who can't afford the premium. Some of the people afford but do not want to pay because they perceive that health is at the hand of God/Allah. Some people say that health insurance is for the poorest. If I get sick, I can be treated at anywhere by paying out of my pocket.

______________________________________________________________________

Code: Premium contribution sytem: better if bank system used {1-0}

P 5: FGD_Women__treatment.docx - 5:27 [I agree with amount of contrib..]  (152:152)   (Super)
Codes:	[Premium contribution sytem: better if bank system used] [Suggestions to improve the cbhi :  improve attitude of health workers on CBHI members] 
No memos

I agree with amount of contribution as said by other participants. But the payment system need to be improved. For instance, if there is back account for health insurance and I save some birr each month, I can finally able to pay the premium easily, even possible to pay more than the current contribution amount. In addition, I recommend that the health services at health centers and hospitals as well as the attitude of health workers towards clients need to be improved. 

______________________________________________________________________

Code: Premium contribution sytem: need improvement(digitalization) {1-0}

P 5: FGD_Women__treatment.docx - 5:26 [As P10 said it is good if the ..]  (151:151)   (Super)
Codes:	[Premium contribution sytem: need improvement(digitalization)] [Premium contribution: better if  breakdown to monthly contributed] [Suggestions to improve the cbhi :  HCWs attitude toward members] [Suggestions to improve the cbhi :  improve service quality] 
No memos

As P10 said it is good if the premium contribution system is based on breakdown to some rounds. We are paying health insurance by reducing from what we eat. Thus, it is goof there is a saving system for health insurance as raised by P10. For me the amount of money contributed for health insurance is not large because nowadays all things including drugs are expensive. But the payment system need to be improved.  Indeed, the equality of health services and health workers attitude towards insured and non-insured clients need improvement. 

______________________________________________________________________

Code: Premium contribution: better if  breakdown to monthly contributed {2-0}

P 5: FGD_Women__treatment.docx - 5:26 [As P10 said it is good if the ..]  (151:151)   (Super)
Codes:	[Premium contribution sytem: need improvement(digitalization)] [Premium contribution: better if  breakdown to monthly contributed] [Suggestions to improve the cbhi :  HCWs attitude toward members] [Suggestions to improve the cbhi :  improve service quality] 
No memos

As P10 said it is good if the premium contribution system is based on breakdown to some rounds. We are paying health insurance by reducing from what we eat. Thus, it is goof there is a saving system for health insurance as raised by P10. For me the amount of money contributed for health insurance is not large because nowadays all things including drugs are expensive. But the payment system need to be improved.  Indeed, the equality of health services and health workers attitude towards insured and non-insured clients need improvement. 

P 5: FGD_Women__treatment.docx - 5:29 [Those who have health insuranc..]  (156:156)   (Super)
Codes:	[PB_CBHI: eraly health seeking compared to not members] [Premium contribution: better if  breakdown to monthly contributed] 
No memos

Those who have health insurance run to health facilities when get sick even if they have no money at their hand.  Individuals who do not visit health facility for their sickness are either those who do not have money from bank or not member of health insurance. Such people ride at home and wait day of their death. Please take our recommendations to those who are working on health insurance, we need to save our money monthly or every some months by opening our account at their office or somewhere they prefer  to contribute health insurance premium.

______________________________________________________________________

Code: Premium contribution: better if monthly contributed {1-0}

P 5: FGD_Women__treatment.docx - 5:25 [Rather than asking people to p..]  (149:149)   (Super)
Codes:	[Premium contribution: better if monthly contributed] 
No memos

Rather than asking people to pay all money at once, it is good if people pay the money every months for some months like Ikub, Idir or in the form of aksiyon. This is my personal suggestion. I prefer paying every month than asking the total money at once. 

______________________________________________________________________

Code: Premium contribution: fair compared to private facility service {2-0}

P 4: FGD_MEN_ treatment.docx - 4:14 [Some communities complain that..]  (131:131)   (Super)
Codes:	[CBHI_SS challenge: why fee Increased] [Premium contribution: fair compared to private facility service] 
No memos

 Some communities complain that the insurance membership fees have increased. This year, the community complains about why health insurance contribution has increased. However, when they compare with private health facilities, they voluntarily become health insurance members because the health insurance contribution is small.

P 7: FGDs_Men___ treatment.docx - 7:12 [So, our people do not have eno..]  (132:132)   (Super)
Codes:	[Premium contribution: fair compared to private facility service] [Reason for not member: lack of awareness] 
No memos

So, our people do not have enough understanding. We have started from two hundred and ninety. We weren't using this before. We don't even have an understanding, except to pay. Now the community is learning. if someone who went here for service and paid in the health facility, when he comes back here, it will be reimbursed. They want to be given enough understanding.  Because if you take one child to the private clinic, the fees you will pay are far than the payment of the insurance for the whole family. So, I think it's beautiful.

______________________________________________________________________

Code: Premium contribution: fair: equivalent with  one time service at private {1-0}

P 6: FGdD_Female_ treatment.docx - 6:1 [In the beginning, we heard tha..]  (125:125)   (Super)
Codes:	[Attitude SS_CBHI: Supportive and recomend] [PB_CBHI:  inclussion of all family memebers for the service] [PB_CBHI: No out of pocket payment] [Premium contribution: fair: equivalent with  one time service at private] 
No memos

In the beginning,  we heard that health insurance be served to get health care without payment. They told us once we paid it would be servi for ourselves and our family. we paid for it and we received receipts based on our payment. And that individual who cannot able to pay can get a free membership. There are three different categories of payment schemes. So the health insurance is very important. The amount we pay for a membership that is served for a year for us and our children may be equivalent to the payment we pay at one visit of service at a private clinic.

______________________________________________________________________

Code: Premium contribution: if we contribute more the service will be improved more {1-0}

P 4: FGD_MEN_ treatment.docx - 4:18 [The amount of contribution dep..]  (138:138)   (Super)
Codes:	[Premium contribution: if we contribute more the service will be improved more] 
No memos

The amount of contribution depends on the supply of medicines and the quality of health services. For example, if the contribution is better, the service we receive is also better. The supply of medicines has also improved. Although we do not know how to calculate, the services available in hospitals and health centers vary. Services have improved this year compared to last year because health insurance contributions have increased and health services have improved.

______________________________________________________________________

Code: premium contribution: It is equal with card cost of private health facilities. {1-0}

P 5: FGD_Women__treatment.docx - 5:17 [But when we compare the health..]  (136:136)   (Super)
Codes:	[premium contribution: It is equal with card cost of private health facilities.] [Premium contribution: too small compared to private facility service] 
No memos

But when we compare the health services with the amount of premium contributed, the contribution is too small. The amount of premium is equal to the amount money that one pay for card at private health facilities. Hiyyessatti kanfaluun yoo cimeeyyuu kafalttin insuransii fayyaa haaromfachuuf kanfalamu kun osoo dhaabbata fayyaa dhuunfaa deemanii fakkaltii kaardii hin caalu. I recommend if the premium contribution is based on their economic status. The three level classification you said is merchant, resident and indigent. Merchant can get money and it is easy for them to contribute.

______________________________________________________________________

Code: Premium contribution: majority are poor {3-0}

P 6: FGdD_Female_ treatment.docx - 6:36 [As we compare with the service..]  (167:167)   (Super)
Codes:	[Premium contribution: majority are poor] [premium contribution: not affordable for some] 
No memos

As we compare with the service we received the payment for the insurance is not as exaggerated but as we compare with our life of standard it was expensive. For example this year, many people challenged to pay. It is very high with our life expenses. If someone has wealth it's not a worry if paid as you want but if you do not have income it is very difficult to pay even though the payment is not much.

P 7: FGDs_Men___ treatment.docx - 7:14 [It is fair.]  (136:136)   (Super)
Codes:	[Premium contribution: majority are poor] 
No memos

 It is fair.

P 7: FGDs_Men___ treatment.docx - 7:25 [t is true that the service we ..]  (158:158)   (Super)
Codes:	[PB_CBHI: nice service] [Premium contribution: majority are poor] [premium contribution: not comparable with incomeaffordable] 
No memos

t is true that the service we received from the government is nice. On the other hand, the income we get is not enough for the current money. Ninety percent of these people have nothing. There is no need to add any more. we are depending on straw, selling straw to live. That's why, I don't think the economy we're responsible for now is enough here

______________________________________________________________________

Code: premium contribution: not affordable for some {4-0}

P 5: FGD_Women__treatment.docx - 5:10 [e main problem is due to lack ..]  (123:123)   (Super)
Codes:	[premium contribution: not affordable for some] 
No memos

e main problem is due to lack of affordability of the premium. There are people suffering from different illness such as diabetic mellitus, HIV, hypertension, etc. There are also elders who need support. These people do not afford the health insurance payment. If these people are considered as indigent group, they are really very happy to be member of health insurance. 

P 5: FGD_Women__treatment.docx - 5:15 [Health insurance can save huge..]  (131:131)   (Super)
Codes:	[premium contribution: not affordable for some] [Premium contribution: too small compared to private facility cost] 
No memos

Health insurance can save huge amount of money for the users. If you go to private clinic, you may pay large amount of money even by a single visit. Indeed, people pay premium when they have money and utilize it when they get sick. Compared to the health services, the amount of premium is too small. But there are many people who are unable afford the premium. Many people are living hand to mouth. Thus, for some people it is affordable, for others such as older people who are unable to work it may not be affordable.

P 6: FGdD_Female_ treatment.docx - 6:33 [Those who are interested can e..]  (162:162)   (Super)
Codes:	[premium contribution: not affordable for some] 
No memos

Those who are interested can engage in insurance and those not can leave it. for some, it may not be fair to pay. For example, if somebody requests insurance payment, he may not have the amount of money. 

P 6: FGdD_Female_ treatment.docx - 6:36 [As we compare with the service..]  (167:167)   (Super)
Codes:	[Premium contribution: majority are poor] [premium contribution: not affordable for some] 
No memos

As we compare with the service we received the payment for the insurance is not as exaggerated but as we compare with our life of standard it was expensive. For example this year, many people challenged to pay. It is very high with our life expenses. If someone has wealth it's not a worry if paid as you want but if you do not have income it is very difficult to pay even though the payment is not much.

______________________________________________________________________

Code: premium contribution: not comparable with incomeaffordable {1-0}

P 7: FGDs_Men___ treatment.docx - 7:25 [t is true that the service we ..]  (158:158)   (Super)
Codes:	[PB_CBHI: nice service] [Premium contribution: majority are poor] [premium contribution: not comparable with incomeaffordable] 
No memos

t is true that the service we received from the government is nice. On the other hand, the income we get is not enough for the current money. Ninety percent of these people have nothing. There is no need to add any more. we are depending on straw, selling straw to live. That's why, I don't think the economy we're responsible for now is enough here

______________________________________________________________________

Code: Premium contribution: Not enough to cover health service {2-0}~

P 4: FGD_MEN_ treatment.docx - 4:17 [The contribution of CBHI is no..]  (137:137)   (Super)
Codes:	[Premium contribution: Not enough to cover health service] 
No memos

The contribution of CBHI is not adequate. Because even medicines can be bought from abroad, it costs a lot of money. Therefore, the contribution for community based health insurance is not enough. Even services are given throughout the year with one time contribution.

P 7: FGDs_Men___ treatment.docx - 7:17 [The households' payment for th..]  (142:142)   (Super)
Codes:	[Premium contribution: Not enough to cover health service] 
No memos

The households' payment for the membership of health insurance is not called a payment. It is less than the service. It is the service which is greater, not the payment. For example, my family, takes on this insurance are eight people. This doesn't give someone a day's service, not refer them or can't give a private clinic, to give a year service for the whole family. This is not called payment.

______________________________________________________________________

Code: Premium contribution: too small compared to private facility cost {1-0}

P 5: FGD_Women__treatment.docx - 5:15 [Health insurance can save huge..]  (131:131)   (Super)
Codes:	[premium contribution: not affordable for some] [Premium contribution: too small compared to private facility cost] 
No memos

Health insurance can save huge amount of money for the users. If you go to private clinic, you may pay large amount of money even by a single visit. Indeed, people pay premium when they have money and utilize it when they get sick. Compared to the health services, the amount of premium is too small. But there are many people who are unable afford the premium. Many people are living hand to mouth. Thus, for some people it is affordable, for others such as older people who are unable to work it may not be affordable.

______________________________________________________________________

Code: Premium contribution: too small compared to private facility service {5-0}

P 5: FGD_Women__treatment.docx - 5:13 [The other reason is due to poo..]  (129:129)   (Super)
Codes:	[high category not meber:  Poor quality of service] [Premium contribution: too small compared to private facility service] 
No memos

The other reason is due to poor quality of health services at public health facilities. For instance, last year, I visited a public health facility after I got sick. They didn't assessed my illness very well. They gave me drugs with wrong diagnosis. I slept my home for about two months. Finally, I went to private clinic, I paid eight thousand birr. The private clinic told me that my illness was related to pregnancy. But the public facility even didn't tell me as I was pregnant. Thus, due to poor quality services at public facilities, people despair for health insurance and refuse to renew the health insurance card. I used to be member of health insurance. This year, however, I refused to renew. I had money but I refused to pay for the health insurance because the health services they provide has no quality. People pay premium but do not get health services. Because of this they decide to dropout from the membership. For those who are not member of health insurance, the health workers provide the all necessary services including laboratory investigation because the health facility need money in cash. 

P 5: FGD_Women__treatment.docx - 5:14 [The amount of premium is too s..]  (129:129)   (Super)
Codes:	[Premium contribution: too small compared to private facility service] 
No memos

The amount of premium is too small but if you do not get services why people spend their money for no use. If I am not able to use it, why I need health insurance card, as it is not book of bank account. Itti hinfayyadamne taanaan suuraa qofa irratti kakaafnee akka dabtara baankii kaardii inshuransii fayyaa baanee jooruun maal nuf godha. 

P 5: FGD_Women__treatment.docx - 5:17 [But when we compare the health..]  (136:136)   (Super)
Codes:	[premium contribution: It is equal with card cost of private health facilities.] [Premium contribution: too small compared to private facility service] 
No memos

But when we compare the health services with the amount of premium contributed, the contribution is too small. The amount of premium is equal to the amount money that one pay for card at private health facilities. Hiyyessatti kanfaluun yoo cimeeyyuu kafalttin insuransii fayyaa haaromfachuuf kanfalamu kun osoo dhaabbata fayyaa dhuunfaa deemanii fakkaltii kaardii hin caalu. I recommend if the premium contribution is based on their economic status. The three level classification you said is merchant, resident and indigent. Merchant can get money and it is easy for them to contribute.

P 7: FGDs_Men___ treatment.docx - 7:23 [Right. This amount is not enou..]  (154:154)   (Super)
Codes:	[Premium contribution: too small compared to private facility service] 
No memos

Right. This amount is not enough for services. The service is the bigger than the payment and the service is beautiful.

P 7: FGDs_Men___ treatment.docx - 7:24 [If I use it as myself, my mone..]  (156:156)   (Super)
Codes:	[Premium contribution: too small compared to private facility service] 
No memos

If I use it as myself, my money is not enough for my family, or not enough for me. I would say that the benefits from the government are more.

______________________________________________________________________

Code: Premium for indigent challenge {1-0}~

P 8: KII HEW_SCH__TREATMENT_TW.docx - 8:11 [According to our region, the r..]  (40:40)   (Super)
Codes:	[Premium for indigent challenge] 
No memos

According to our region, the rich pay to become members of CBHI but are treated privately. At this time, the money they pay to buy medicines benefits the poor. There were also times when we went out in a campaign manner and begged government employees, schools, and landlords to pay for the poor.

______________________________________________________________________

Code: premium is not high {0-0}
______________________________________________________________________

Code: premium not much high {0-0}~
______________________________________________________________________

Code: reason for dropout: ilegaly registered members fear after digitalization {1-0}

P 5: FGD_Women__treatment.docx - 5:24 [The dropout from health insura..]  (147:147)   (Super)
Codes:	[reason for dropout: ilegaly registered members fear after digitalization] [Reason for not member: poor quality of service] 
No memos

The dropout from health insurance membership is due to updated registration system which means initially the members are recorded on hard copy. During that period non-illegible people such as governmental employees, and those retired were member of health insurance as it is difficult to identify them from hardcopy. But currently the registration system is computerized. Due to this non-illegible individuals are dropout because they fear that the computer can catch them as it is illegal. The other reason for dropout is due to poor quality of health services as it is mentioned by other participants. 

______________________________________________________________________

Code: Reason for full coverage_SS_CBHI: medicine availability {1-0}

P13: KII _ _HC-Head _ Ttreatment.docx - 13:1 [The performance of health insu..]  (11:11)   (Super)
Codes:	[Reason for full coverage_SS_CBHI: medicine availability] [Reason for full coverage_SS_CBHI: planning probblem] [Reason for full coverage_SS_CBHI: respectful and compassionate care] 
No memos

The performance of health insurance as per our catchment is good, which is more 100 per cent. The community has enough awareness because it has been using it for a long time. Among the factors that contribute to good performance are providing good receptions during health services, ensuring the availability necessary supplies including medicines, and making the community voluntary members of community based health insurance.

______________________________________________________________________

Code: Reason for full coverage_SS_CBHI: planning probblem {1-0}

P13: KII _ _HC-Head _ Ttreatment.docx - 13:1 [The performance of health insu..]  (11:11)   (Super)
Codes:	[Reason for full coverage_SS_CBHI: medicine availability] [Reason for full coverage_SS_CBHI: planning probblem] [Reason for full coverage_SS_CBHI: respectful and compassionate care] 
No memos

The performance of health insurance as per our catchment is good, which is more 100 per cent. The community has enough awareness because it has been using it for a long time. Among the factors that contribute to good performance are providing good receptions during health services, ensuring the availability necessary supplies including medicines, and making the community voluntary members of community based health insurance.

______________________________________________________________________

Code: Reason for full coverage_SS_CBHI: political commitment {1-0}

P14: KII HEW__2024_.docx - 14:1 [We have now reached 100 percen..]  (10:10)   (Super)
Codes:	[Reason for full coverage_SS_CBHI: political commitment] [Reason for full coverage_SS_CBHI: positive reinforcement] 
No memos

We have now reached 100 percent coverage. How we were able to reach 100%, there is a cabinet deployed from the . And the kebele leader has taken this as a priority. It means we even consulted the zone/gote owners. We had a competition. It meant that we were ranking each other as to which of the three zone owners collected the most.   For these reasons, we have been successful with health insurance.

______________________________________________________________________

Code: Reason for full coverage_SS_CBHI: positive reinforcement {1-0}

P14: KII HEW__2024_.docx - 14:1 [We have now reached 100 percen..]  (10:10)   (Super)
Codes:	[Reason for full coverage_SS_CBHI: political commitment] [Reason for full coverage_SS_CBHI: positive reinforcement] 
No memos

We have now reached 100 percent coverage. How we were able to reach 100%, there is a cabinet deployed from the . And the kebele leader has taken this as a priority. It means we even consulted the zone/gote owners. We had a competition. It meant that we were ranking each other as to which of the three zone owners collected the most.   For these reasons, we have been successful with health insurance.

______________________________________________________________________

Code: Reason for full coverage_SS_CBHI: respectful and compassionate care {1-0}

P13: KII _ _HC-Head _ Ttreatment.docx - 13:1 [The performance of health insu..]  (11:11)   (Super)
Codes:	[Reason for full coverage_SS_CBHI: medicine availability] [Reason for full coverage_SS_CBHI: planning probblem] [Reason for full coverage_SS_CBHI: respectful and compassionate care] 
No memos

The performance of health insurance as per our catchment is good, which is more 100 per cent. The community has enough awareness because it has been using it for a long time. Among the factors that contribute to good performance are providing good receptions during health services, ensuring the availability necessary supplies including medicines, and making the community voluntary members of community based health insurance.

______________________________________________________________________

Code: Reason for full coverage_SS_CBHI: service quality {0-0}
______________________________________________________________________

Code: Reason for not member: lack of awareness {3-0}

P 7: FGDs_Men___ treatment.docx - 7:8 [there is a lack of awareness a..]  (124:124)   (Super)
Codes:	[Reason for not member: lack of awareness] 
No memos

there is a lack of awareness and lack of understanding. Even I myself thought it was useless before

P 7: FGDs_Men___ treatment.docx - 7:10 [There is nothing to hinder it ..]  (126:126)   (Super)
Codes:	[Reason for not member: lack of awareness] 
No memos

There is nothing to hinder it except community awareness. It is very nice and the health facilities are very nice. The people have now understood, and everyone is now asking for this insurance.

P 7: FGDs_Men___ treatment.docx - 7:12 [So, our people do not have eno..]  (132:132)   (Super)
Codes:	[Premium contribution: fair compared to private facility service] [Reason for not member: lack of awareness] 
No memos

So, our people do not have enough understanding. We have started from two hundred and ninety. We weren't using this before. We don't even have an understanding, except to pay. Now the community is learning. if someone who went here for service and paid in the health facility, when he comes back here, it will be reimbursed. They want to be given enough understanding.  Because if you take one child to the private clinic, the fees you will pay are far than the payment of the insurance for the whole family. So, I think it's beautiful.

______________________________________________________________________

Code: Reason for not member: lack of knowledge {1-0}

P 6: FGdD_Female_ treatment.docx - 6:12 [A person who does not particip..]  (135:136)   (Super)
Codes:	[Reason for not member: lack of knowledge] 
No memos

A person who does not participate in meetings, a person who does not know his rights and responsibilities. Those individuals are those who do not know what benefits or not. If they know the importance of it they may become the member of the insurance. 
Even those has wealth are using health insurance

______________________________________________________________________

Code: Reason for not member: lack of trust {1-0}

P 7: FGDs_Men___ treatment.docx - 7:7 [New insurance members and even..]  (122:122)   (Super)
Codes:	[Reason for not member: lack of trust] 
No memos

New insurance members and even the whole community need pressure and motivation to be members. People were running away as if the government was taking the money from them and spending it on something else.

______________________________________________________________________

Code: Reason for not member: Negative attitude {1-0}

P 4: FGD_MEN_ treatment.docx - 4:11 [There were attitude problems t..]  (124:124)   (Super)
Codes:	[Reason for not member: Negative attitude] [Reason for not member: poor reception of health workers] 
No memos

There were attitude problems that prevented some people from becoming insurance members. In addition, the lack of good reception by health professionals can be a barrier.

______________________________________________________________________

Code: Reason for not member: no medicine {1-0}

P 5: FGD_Women__treatment.docx - 5:11 [But due to lack of supplies es..]  (125:125)   (Super)
Codes:	[Reason for not member: no medicine] 
No memos

But due to lack of supplies especially medicines, some people dropout from health insurance membership or refuse to renew their health insurance card.

______________________________________________________________________

Code: Reason for not member: poor quality of service {1-0}

P 5: FGD_Women__treatment.docx - 5:24 [The dropout from health insura..]  (147:147)   (Super)
Codes:	[reason for dropout: ilegaly registered members fear after digitalization] [Reason for not member: poor quality of service] 
No memos

The dropout from health insurance membership is due to updated registration system which means initially the members are recorded on hard copy. During that period non-illegible people such as governmental employees, and those retired were member of health insurance as it is difficult to identify them from hardcopy. But currently the registration system is computerized. Due to this non-illegible individuals are dropout because they fear that the computer can catch them as it is illegal. The other reason for dropout is due to poor quality of health services as it is mentioned by other participants. 

______________________________________________________________________

Code: Reason for not member: poor reception of health workers {1-0}

P 4: FGD_MEN_ treatment.docx - 4:11 [There were attitude problems t..]  (124:124)   (Super)
Codes:	[Reason for not member: Negative attitude] [Reason for not member: poor reception of health workers] 
No memos

There were attitude problems that prevented some people from becoming insurance members. In addition, the lack of good reception by health professionals can be a barrier.

______________________________________________________________________

Code: Reason for not member: some lack of awareness {1-0}

P 7: FGDs_Men___ treatment.docx - 7:6 [Thank you. Which community gro..]  (119:120)   (Super)
Codes:	[Reason for not member: some lack of awareness] 
No memos

Thank you. Which community groups are often involved and which are not? Which community groups are most involved in this health insurance membership, and which are not?
P6: This depends on their level of understanding. Even I myself used think this health insurance doesn't have benefits. This thing will become a regret for you later, I told to those who don't have it now. He says he's seen the results of it and even someone I've seen the results of it myself.

______________________________________________________________________

Code: Reason for not member: there are some individuals unable to pay {1-0}

P 4: FGD_MEN_ treatment.docx - 4:10 [Some households may not partic..]  (123:123)   (Super)
Codes:	[Reason for not member: there are some individuals unable to pay] 
No memos

Some households may not participate in insurance due to not able to pay. Other than that, there is nothing that is prevented them to be a community based health insurance member

______________________________________________________________________

Code: Reasons for not becoming member_SS_CBHI: absence of reimbursement for clients {1-0}

P19: KII_Head of  health Office_ treatment.docx - 19:25 [The other reason may be due to..]  (51:51)   (Super)
Codes:	[Reasons for not becoming member_SS_CBHI: absence of reimbursement for clients] 
No memos

The other reason may be due to absence of reimbursement for individuals who buy drugs from private pharmacies which have no agreement with our office. 

______________________________________________________________________

Code: Reasons for not becoming member_SS_CBHI: awareness {4-0}

P13: KII _ _HC-Head _ Ttreatment.docx - 13:9 [Due to lack of understanding, ..]  (27:27)   (Super)
Codes:	[Reasons for not becoming member_SS_CBHI: awareness] [Reasons for not becoming member_SS_CBHI: inability to pay] [Reasons for not becoming member_SS_CBHI: service quality] 
No memos

Due to lack of understanding, they may not be members of community based health insurance. Everyone has needs. There may be those who are not members of health insurance due to lack of money. Some household may be not enrolled to community based health insurance due to lack of adequate services.

P13: KII _ _HC-Head _ Ttreatment.docx - 13:10 [They also do not renew due to ..]  (29:29)   (Super)
Codes:	[Reasons for not becoming member_SS_CBHI: awareness] [Reasons for not becoming member_SS_CBHI: health status] 
No memos

They also do not renew due to lack of adequate services and lack of understanding. There are some people who say that I should not pay this year because I paid last year and I am not sick. 

P15: KII PHCU Leader___TREATMENT TW.docx - 15:9 [The first obstacle is the lack..]  (24:24)   (Super)
Codes:	[Reasons for not becoming member_SS_CBHI: awareness] [Reasons for not becoming member_SS_CBHI: medicine unavailablity] [Service gap_SS_CBHI: medicine availability] 
No memos

The first obstacle is the lack of awareness in the community. Another is that medicines are often scarce in the health center, so they think that they would have bought medicines from outside, and what would membership do for them? 

P16: KII__HEW_ _ _.docx - 16:11 [Some households do not get enr..]  (27:27)   (Super)
Codes:	[Reasons for not becoming member_SS_CBHI: awareness] [Reasons for not becoming member_SS_CBHI: poor service] 
No memos

Some households do not get enrolled into community based health insurance because of lack of understanding. A few households (rich) will remain non-members because they want to be treated at their own expense. 

______________________________________________________________________

Code: Reasons for not becoming member_SS_CBHI: fairness on identifying indigent HHs {0-0}
______________________________________________________________________

Code: Reasons for not becoming member_SS_CBHI: family health status {2-0}

P16: KII__HEW_ _ _.docx - 16:7 [There are some barriers for pa..]  (23:23)   (Super)
Codes:	[Reasons for not becoming member_SS_CBHI: family health status] 
No memos

There are some barriers for participation such as lack of understanding. For example, there are some people who say that when we get sick we will be treated with our own money instead of contributing now. However, if you make that clear, they will pay/participate.

P16: KII__HEW_ _ _.docx - 16:13 [I paid for community based hea..]  (29:29)   (Super)
Codes:	[Barriers_SS_CBHI: attitude and awareness] [Reasons for not becoming member_SS_CBHI: family health status] 
No memos

I paid for community based health insurance last year, but I didn't go to the health facility even for a single day last year because I didn't get sick. So there are people who say that I should not pay insurance this year because I paid it last year but not served.

______________________________________________________________________

Code: Reasons for not becoming member_SS_CBHI: health status {1-0}

P13: KII _ _HC-Head _ Ttreatment.docx - 13:10 [They also do not renew due to ..]  (29:29)   (Super)
Codes:	[Reasons for not becoming member_SS_CBHI: awareness] [Reasons for not becoming member_SS_CBHI: health status] 
No memos

They also do not renew due to lack of adequate services and lack of understanding. There are some people who say that I should not pay this year because I paid last year and I am not sick. 

______________________________________________________________________

Code: Reasons for not becoming member_SS_CBHI: health workers behaviour {3-0}

P17: KII_CBHI Focal__ treatment.docx - 17:31 [In 2009 in _ , CB..]  (45:45)   (Super)
Codes:	[Reasons for not becoming member_SS_CBHI: health workers behaviour] [Service gap_SS_CBHI: health workers behaviour] 
No memos

In 2009 in _ , CBHI was 50%. Then, decline to 21%, and 23%. Then, rise up to 70%, and 66%.  In 2015 and 2016, we achieved 100%. One of the reason for non-membership was due to health workers perception towards CBHI. Previously, health workers were less interested to serve those who are insurance member.

P17: KII_CBHI Focal__ treatment.docx - 17:32 [They were giving priority for ..]  (45:45)   (Super)
Codes:	[Reasons for not becoming member_SS_CBHI: health workers behaviour] [Service gap_SS_CBHI: health workers behaviour] 
No memos

They were giving priority for those who are not CBHI member. This was due to the need for internal income generation. Health workers were perceiving that CBHI can affect the internal revenue of the health centers

P19: KII_Head of  health Office_ treatment.docx - 19:26 [Thus, lack of accessibility of..]  (51:51)   (Super)
Codes:	[Reasons for not becoming member_SS_CBHI: health workers behaviour] [Reasons for not becoming member_SS_CBHI: medicine unavailablity] [Service gap_SS_CBHI: health workers behaviour] [Service gap_SS_CBHI: medicine availability] 
No memos

Thus, lack of accessibility of drugs are one of the reasons for not membership or defaulters. The other reason may be due to lack of compassionate services of the community. Some health workers disrespect the clients. This annoy the community and make the people not become a CBHI member. Some of the community member also complain that health workers give favor for those are not member of CBHI.

______________________________________________________________________

Code: Reasons for not becoming member_SS_CBHI: human and financial resource {1-0}

P12: KII CBHI focal___TREATMENT TW.docx - 12:10 [When we talk about the quality..]  (27:27)   (Super)
Codes:	[Challenge_SS_CBHI: resources] [Reasons for not becoming member_SS_CBHI: human and financial resource] [Reasons for not becoming member_SS_CBHI: poor quality] 
No memos

When we talk about the quality of health care, we mean giving someone who has come in need what they want in a complete way. This may not happen due to problems such as a lack of medical equipment and human resources. Therefore, there are still deficiencies in health quality that need to be improved. This decline in health quality, along with other problems, can cause problems in the recruitment and retention of CBHI members.

______________________________________________________________________

Code: Reasons for not becoming member_SS_CBHI: ignorance of HWs CBHI users {1-0}

P17: KII_CBHI Focal__ treatment.docx - 17:35 [The other reason for non-membe..]  (45:45)   (Super)
Codes:	[challenge_SS_CBH: priority for payers] [Reasons for not becoming member_SS_CBHI: ignorance of HWs CBHI users] 
No memos

The other reason for non-membership was due to lack of compassionate health workers. In _ , we made two windows at card room: one for insurance member and the other for MCH/exempted services. This is to serve all clients equally as we had been hearing complain from the clients about the existence of delayed services for MCH/exempted services.  

______________________________________________________________________

Code: Reasons for not becoming member_SS_CBHI: inability to pay {1-0}

P13: KII _ _HC-Head _ Ttreatment.docx - 13:9 [Due to lack of understanding, ..]  (27:27)   (Super)
Codes:	[Reasons for not becoming member_SS_CBHI: awareness] [Reasons for not becoming member_SS_CBHI: inability to pay] [Reasons for not becoming member_SS_CBHI: service quality] 
No memos

Due to lack of understanding, they may not be members of community based health insurance. Everyone has needs. There may be those who are not members of health insurance due to lack of money. Some household may be not enrolled to community based health insurance due to lack of adequate services.

______________________________________________________________________

Code: Reasons for not becoming member_SS_CBHI: medicine unavailablity {7-0}

P11: KII _Town HO head_ Treatment.docx - 11:16 [As it knows our country is a d..]  (40:40)   (Super)
Codes:	[Reasons for not becoming member_SS_CBHI: medicine unavailablity] 
No memos

As it knows our country is a developing country and above 93% of medicines are imported from abroad.  It is very rare the drugs to be produced in the country. Due to problems with currency exchange, and other problems, the drug supply by the government has its own problems.

P14: KII HEW__2024_.docx - 14:17 [Some households say that they ..]  (31:31)   (Super)
Codes:	[Reasons for not becoming member_SS_CBHI: medicine unavailablity] 
No memos

Some households say that they do not get enough medical services at the health center and do not get medicines. 

P15: KII PHCU Leader___TREATMENT TW.docx - 15:9 [The first obstacle is the lack..]  (24:24)   (Super)
Codes:	[Reasons for not becoming member_SS_CBHI: awareness] [Reasons for not becoming member_SS_CBHI: medicine unavailablity] [Service gap_SS_CBHI: medicine availability] 
No memos

The first obstacle is the lack of awareness in the community. Another is that medicines are often scarce in the health center, so they think that they would have bought medicines from outside, and what would membership do for them? 

P17: KII_CBHI Focal__ treatment.docx - 17:33 [The other reason was due to la..]  (45:45)   (Super)
Codes:	[Reasons for not becoming member_SS_CBHI: medicine unavailablity] 
No memos

The other reason was due to lack of availability of drugs. Nowadays, we are inform the communities about the reasons for unavailability of drugs. 

P18: KII_CBHI focal__ treatment.docx - 18:19 [You know it is impossible to a..]  (25:25)   (Super)
Codes:	[Barriers_SS_CBHI: medicine unavailable] [Reasons for not becoming member_SS_CBHI: medicine unavailablity] 
No memos

You know it is impossible to avail all medicines in all health facilities; our community my expect that they can get all the services they need at any time. When this expectation does not meet, they may dissatisfy with their membership and may decide not to renew their membership. Therefore, in my opinion, major factor that affect the level of community's participation in CBHI is affected by medicine unavailability. Even sometimes, you cannot get some types of medicine from EPSS and on the private market; at this time our client cannot find the medicine even from private pharmacies. 

P19: KII_Head of  health Office_ treatment.docx - 19:26 [Thus, lack of accessibility of..]  (51:51)   (Super)
Codes:	[Reasons for not becoming member_SS_CBHI: health workers behaviour] [Reasons for not becoming member_SS_CBHI: medicine unavailablity] [Service gap_SS_CBHI: health workers behaviour] [Service gap_SS_CBHI: medicine availability] 
No memos

Thus, lack of accessibility of drugs are one of the reasons for not membership or defaulters. The other reason may be due to lack of compassionate services of the community. Some health workers disrespect the clients. This annoy the community and make the people not become a CBHI member. Some of the community member also complain that health workers give favor for those are not member of CBHI.

P20: KII_Kebele manager__treatment.docx - 20:20 [As I told you earlier, the mai..]  (25:25)   (Super)
Codes:	[Reasons for not becoming member_SS_CBHI: medicine unavailablity] 
No memos

As I told you earlier, the main reason is unavailability of services like medicines; you know our community lives together and shares their experiences about healthcare services. When one client did not get enough services from health center, like when they bought medicine from outside, or if they disrespected due to their membership status, they share it with their community after returned to their village, which strongly demoralize others and push some individuals from this CBHI membership. 

______________________________________________________________________

Code: Reasons for not becoming member_SS_CBHI: percieve wrong medicine {1-0}

P14: KII HEW__2024_.docx - 14:18 [There are some people who refu..]  (31:31)   (Super)
Codes:	[Reasons for not becoming member_SS_CBHI: percieve wrong medicine] 
No memos

There are some people who refuse to pay for the increase of premium contribution of this year and when we go to the health center, we are prescribed same kind of medicine and therefore we will not pay.

______________________________________________________________________

Code: Reasons for not becoming member_SS_CBHI: poor quality {1-0}

P12: KII CBHI focal___TREATMENT TW.docx - 12:10 [When we talk about the quality..]  (27:27)   (Super)
Codes:	[Challenge_SS_CBHI: resources] [Reasons for not becoming member_SS_CBHI: human and financial resource] [Reasons for not becoming member_SS_CBHI: poor quality] 
No memos

When we talk about the quality of health care, we mean giving someone who has come in need what they want in a complete way. This may not happen due to problems such as a lack of medical equipment and human resources. Therefore, there are still deficiencies in health quality that need to be improved. This decline in health quality, along with other problems, can cause problems in the recruitment and retention of CBHI members.

______________________________________________________________________

Code: Reasons for not becoming member_SS_CBHI: poor service {3-0}

P11: KII _Town HO head_ Treatment.docx - 11:13 [Also, a perceived shortage of ..]  (30:30)   (Super)
Codes:	[Reasons for not becoming member_SS_CBHI: poor service] [Service gap_SS_CBHI: medicine, human resource unavailable] 
No memos

Also, a perceived shortage of logistics and supply is considered that the private facilities are better in human work, supply, and equipment. So the perception of insurance service the service the poor people derived from this issue. So it is not striking the health insurance but there is the perception that there are not enough supplies in government facilities. That is many people did not want to the members of insurance. 

P12: KII CBHI focal___TREATMENT TW.docx - 12:9 [Initially, the CBHI membership..]  (25:25)   (Super)
Codes:	[Reasons for not becoming member_SS_CBHI: poor service] [Reasons for not becoming member_SS_CBHI: short time] 
No memos

Initially, the CBHI membership recruitment period is limited, and the community may lack money during that period. Next, even staff may not want to give time and repeatedly try to convince and make members. Another is the withdrawal from membership due to a few shortcomings in the health services. Generally, the main reasons are complaints about a lack of health services and funds.

P16: KII__HEW_ _ _.docx - 16:11 [Some households do not get enr..]  (27:27)   (Super)
Codes:	[Reasons for not becoming member_SS_CBHI: awareness] [Reasons for not becoming member_SS_CBHI: poor service] 
No memos

Some households do not get enrolled into community based health insurance because of lack of understanding. A few households (rich) will remain non-members because they want to be treated at their own expense. 

______________________________________________________________________

Code: Reasons for not becoming member_SS_CBHI: premium collection process {0-0}
______________________________________________________________________

Code: Reasons for not becoming member_SS_CBHI: security {1-0}

P15: KII PHCU Leader___TREATMENT TW.docx - 15:10 [Third, it was difficult to ent..]  (24:24)   (Super)
Codes:	[Reasons for not becoming member_SS_CBHI: security] 
No memos

Third, it was difficult to enter the community to provide information and collect premium contributions due to security problems.

______________________________________________________________________

Code: Reasons for not becoming member_SS_CBHI: service quality {1-0}

P13: KII _ _HC-Head _ Ttreatment.docx - 13:9 [Due to lack of understanding, ..]  (27:27)   (Super)
Codes:	[Reasons for not becoming member_SS_CBHI: awareness] [Reasons for not becoming member_SS_CBHI: inability to pay] [Reasons for not becoming member_SS_CBHI: service quality] 
No memos

Due to lack of understanding, they may not be members of community based health insurance. Everyone has needs. There may be those who are not members of health insurance due to lack of money. Some household may be not enrolled to community based health insurance due to lack of adequate services.

______________________________________________________________________

Code: Reasons for not becoming member_SS_CBHI: short time {1-0}

P12: KII CBHI focal___TREATMENT TW.docx - 12:9 [Initially, the CBHI membership..]  (25:25)   (Super)
Codes:	[Reasons for not becoming member_SS_CBHI: poor service] [Reasons for not becoming member_SS_CBHI: short time] 
No memos

Initially, the CBHI membership recruitment period is limited, and the community may lack money during that period. Next, even staff may not want to give time and repeatedly try to convince and make members. Another is the withdrawal from membership due to a few shortcomings in the health services. Generally, the main reasons are complaints about a lack of health services and funds.

______________________________________________________________________

Code: Recommendation_SS_CBHI: Advance paymment for facilities {1-0}

P11: KII _Town HO head_ Treatment.docx - 11:21 [So the insurance agency has to..]  (43:43)   (Super)
Codes:	[Recommendation_SS_CBHI: Advance paymment for facilities] 
No memos

So the insurance agency has to borrow the collected money for health facilities to strengthen services.  In a developed country, a person becomes an insurance member for his property which is inconsistent with our case in which people resist membership health insurance. 


______________________________________________________________________

Code: Recommendation_SS_CBHI: awareness {1-0}

P15: KII PHCU Leader___TREATMENT TW.docx - 15:15 [In my opinion, if the necessar..]  (36:36)   (Super)
Codes:	[Recommendation_SS_CBHI: awareness] [Recommendation_SS_CBHI: service quality] 
No memos

 In my opinion, if the necessary items, such as medical equipment and medicines, are provided to the health facility in time, the community can participate in this program without any problems. Furthermore, if information about the CBHI is made available to health professionals, the community, and all relevant stakeholders in advance, it is very good for timely preparation.

______________________________________________________________________
[truncated: 262,496 more chars]
