# Supplementary material for: Early community reactions and acceptance of the Sliding-Scale Community Based Health Insurance Scheme in Ethiopia: Qualitative Findings from the treatment arm
Source: PLoS One. 2026 Jul 22;21(7):e0353876. doi: 10.1371/journal.pone.0353876 (PMC13390935; doi:10.1371/journal.pone.0353876)
Supplement: S4 Appendix — (DOCX) [file pone.0353876.s004.docx]

Appendix S4_Resources mobilized in 2016 compared to 2015.

| **District** | **2023 Flat-Rate (ETB)** | **2023 Flat-Rate (USD)** | **2024 Sliding-Scale (ETB)** | **2024 Sliding-Scale (USD)** |
| --- | --- | --- | --- | --- |
| Adaba | 13,500,000 | 251,866 | 32,200,000 | 575,000 |
| Dawo | 4,800,000 | 89,552 | 16,100,000 | 287,500 |
| Elu | 3,000,000 | 55,970 | 6,500,000 | 116,071 |
| Ambo town | 3,600,000 | 67,164 | 10,700,000 | 191,071 |
| Deder | 11,100,000 | 207,090 | 52,100,000 | 930,357 |
| Gorogutu | 14,800,000 | 276,119 | 33,100,000 | 591,071 |
| Borga Dintu | 4,200,000 | 78,358 | 13,600,000 | 242,857 |
| Asela Town | 6,800,000 | 126,866 | 10,100,000 | 180,357 |
| Kimblibit | 7,000,000 | 130,597 | 12,300,000 | 219,643 |
| Dara | 2,500,000 | 46,642 | 3,200,000 | 57,143 |
| Wondo Genet Town | 1,100,000 | 20,522 | 2,600,000 | 46,429 |
| Aleta Wondo | 3,800,000 | 70,896 | 9,200,000 | 164,286 |
| Aleta Chuko | 3,000,000 | 55,970 | 3,900,000 | 69,643 |
| **Overall** | **86,800,000** | **1,619,403** | **204,100,000** | **3,644,643** |

**Source: EHIS report [Administrative data]**
